# Linking prostate cancer cell AR heterogeneity to distinct castration and enzalutamide responses

**DOI:** 10.1038/s41467-018-06067-7

**Published:** 2018-09-06

**Authors:** Qiuhui Li, Qu Deng, Hsueh-Ping Chao, Xin Liu, Yue Lu, Kevin Lin, Bigang Liu, Gregory W. Tang, Dingxiao Zhang, Amanda Tracz, Collene Jeter, Kiera Rycaj, Tammy Calhoun-Davis, Jiaoti Huang, Mark A. Rubin, Himisha Beltran, Jianjun Shen, Gurkamal Chatta, Igor Puzanov, James L. Mohler, Jianmin Wang, Ruizhe Zhao, Jason Kirk, Xin Chen, Dean G. Tang

**Affiliations:** 1 Department of Pharmacology and Therapeutics, Roswell Park Comprehensive Cancer Center, Buffalo, NY 14263 USA; 2 Department of Epigenetics and Molecular Carcinogenesis, University of Texas M.D. Anderson Cancer Center, Science Park, Smithville, TX 78957 USA; 3 State Key Laboratory Breeding Base of Basic Science of Stomatology (Hubei-MOST) and Key Laboratory for Oral Biomedicine of Ministry of Education (KLOBM), School and Hospital of Stomatology,, Wuhan University, 430079 Wuhan, China; 4 Program in Molecular Carcinogenesis, University of Texas Graduate School for Biomedical Sciences (GSBS), Houston, TX 77030 USA; 5 Department of Pathology, Duke University of School of Medicine, Durham, NC 27710 USA; 6 Caryl and Israel Englander Institute for Precision Medicine, New York-Presbyterian Hospital, Weill Cornell Medical College, New York, NY 10065 USA; 7 Sandra and Edward Meyer Cancer Center at Weill Cornell Medicine, New York, NY 10021 USA; 8 Department of Medicine, Roswell Park Comprehensive Cancer Center, Buffalo, NY 14263 USA; 9 Department of Urology, Roswell Park Comprehensive Cancer Center, Buffalo, NY 14263 USA; 10 Department of Biostatistics and Bioinformatics, Roswell Park Comprehensive Cancer Center, Buffalo, NY 14263 USA; 11 Department of Oncology, Tongji Hospital, Tongji Medical College, Huazhong University of Science and Technology, 430030 Wuhan, China; 12 Cancer Stem Cell Institute, Research Center for Translational Medicine, East Hospital, Tongji University School of Medicine, 200120 Shanghai, China

## Abstract

Expression of androgen receptor (AR) in prostate cancer (PCa) is heterogeneous but the functional significance of AR heterogeneity remains unclear. Screening ~200 castration-resistant PCa (CRPC) cores and whole-mount sections (from 89 patients) reveals 3 AR expression patterns: nuclear (nuc-AR), mixed nuclear/cytoplasmic (nuc/cyto-AR), and low/no expression (AR^−/lo^). Xenograft modeling demonstrates that AR^+^ CRPC is enzalutamide-sensitive but AR^−/lo^ CRPC is resistant. Genome editing-derived AR^+^ and AR-knockout LNCaP cell clones exhibit distinct biological and tumorigenic properties and contrasting responses to enzalutamide. RNA-Seq and biochemical analyses, coupled with experimental combinatorial therapy, identify BCL-2 as a critical therapeutic target and provide proof-of-concept therapeutic regimens for both AR^+/hi^ and AR^−/lo^ CRPC. Our study links AR expression heterogeneity to distinct castration/enzalutamide responses and has important implications in understanding the cellular basis of prostate tumor responses to AR-targeting therapies and in facilitating development of novel therapeutics to target AR^−/lo^ PCa cells/clones.

## Abstract (web summary)

The functional significance of the observed heterogeneity of androgen receptor (AR) expression in prostate cancer is unknown. Here the authors show AR expression heterogeneity is associated with distinct castration/enzalutamide responses and identify BCL-2 as a potential therapeutic target in castration-resistant prostate cancer.

## Introduction

Androgen receptor (AR), a steroid hormone receptor normally activated by androgens, plays an essential role in prostate cancer (PCa) development, progression, and therapy response^1^. Most PCa patients are first treated by radical prostatectomy and/or radiation therapy. When post treatment serum PSA (prostate-specific antigen) levels rise, the patient is treated by first-line androgen deprivation therapy (ADT) using GnRH analogs, which suppress gonadal production of testosterone (T), and PCa cells at this stage are castration sensitive (Supplementary Fig. 1a). Increasing PSA levels indicate the recurrence of primary castration-resistant PCa (CRPC) and the patient is then put on second-line regimens to suppress AR function (using enzalutamide; Enza) and/or block adrenal androgen biosynthesis (using abiraterone). Patients will eventually experience Enza-resistant secondary CRPC with a shorter interval due to acquired resistance (Supplementary Fig. 1a). Molecular mechanisms underlying (primary) castration and (secondary) Enza resistance are incompletely understood.

Both chemical castration (using ADT and abiraterone) and antiandrogens (Enza and early-generation drugs such as bicalutamide) target AR signaling. However, human PCa is heterogeneous containing both AR-expressing (AR^+^), as well as AR low-expressing or non-expressing (AR^−/lo^) cells and this AR heterogeneity is accentuated in advanced metastatic and relapsed PCa^2–14^. Whether the heterogeneity in AR expression levels impacts PCa biology and therapy response remains unclear. This project is undertaken to address this important question and to fill a critical gap in our knowledge. Through extensive xenograft modeling, development of AR-tagged (AR^+^) and AR-knockout (KO) LNCaP cell clones, and performing in vitro biological and in vivo tumor regeneration assays, RNA-Seq, and multiple combinatorial therapeutic experiments, we link the AR expression status to distinct tumorigenic behavior and castration/Enza responses. Critically, our studies uncover signaling molecules and pathways underlying the development of, and also establish proof-of-principle therapeutic regimens targeting, the two distinct castration resistance modes mediated by AR^+/hi^ and AR^−/lo^ PCa cells, respectively.

## Results

### Three distinct expression patterns of AR in CRPC

**Fig. 1:**
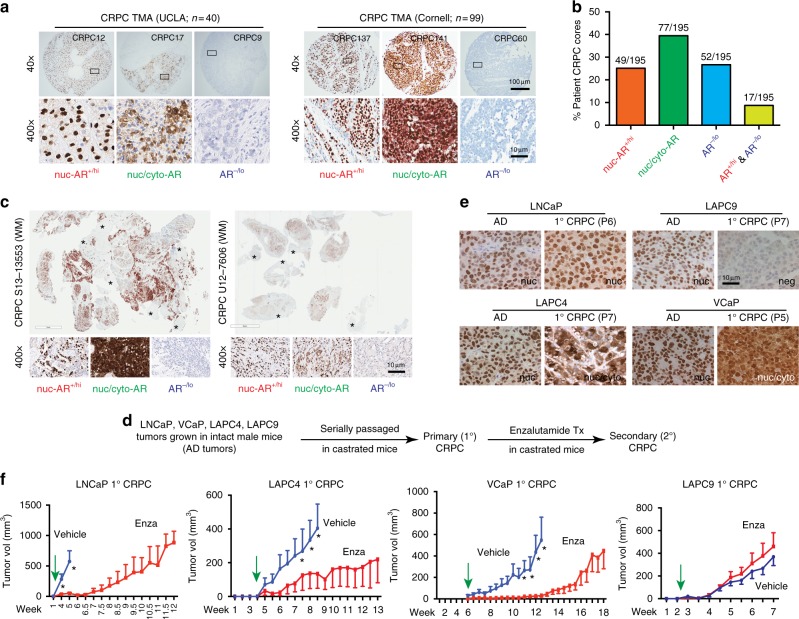
Linking AR heterogeneity in CRPC to distinct Enza response. **a**, **b** AR IHC images (**a**) and quantification of AR expression patterns (**b**) in 195 CRPC cores from three TMAs. Original magnifications and scale bars are indicated. **c** AR IHC in WM sections from two CRPC patients (i.e.# S13-13553 and U12-7606). Shown below are 3 AR patterns (original magnifications and scale bar indicated). *AR^−/lo^ areas. **d** Experimental scheme to generate primary (1°) and secondary (2°) xenograft CRPC. Tx, treatment. **e** AR IHC in 4 AD/1° CRPC (P, passage number) xenografts showing 3 AR expression patterns (nuc-AR^+/hi^, nuc/cyto-AR, or AR^−/lo^) (all 400×; scale bar indicated in one panel). Note that although LAPC4 CRPC is classified as nuc/cyto-AR, the main AR expression pattern is cytoplasmic. **f** Therapeutic responses of 4 xenograft CRPCs to Enza. Presented are tumor volumes (mm^3^; note that only half of the error bars were shown to avoid crowdedness in the line graphs) as a function of treatment time. Green arrows indicate the starting point of Enza treatment. *P < 0.01 (Student’s *t*-test; *n* = 10 for the vehicle and Enza groups, respectively, for each tumor model)

We first assess AR expression levels and distribution patterns in sections from 3 tissue microarrays (TMAs) that contain 195 CRPC cores derived from 81 patient CRPC samples (Fig. 1a–c; Supplementary Fig. 1b-d), most of which are the prostates treated in the pre-Enza era (Supplementary Data 1). Immuno-histochemical (IHC) staining of AR using an N-terminally directed monoclonal antibody (ab74272; Supplementary Table 1), which would recognize full-length AR and all C-terminal truncated variants, reveals 3 distinct patterns of AR expression (Fig. 1a, b; Supplementary Fig. 1b, c): (1) primarily nuclear AR (nuc-AR^+/hi^; 49 cores, or 25% of the total); (2) both nuclear and cytoplasmic AR (nuc/cyto-AR; 77 cores or 39% of the total), and (3) lack of appreciable AR expression (AR^−/lo^; 52 cores, ~27% of the total). The remaining 17 cores (9%) contain both AR^+^ and AR^−/lo^ cells (Fig. 1b; Supplementary Fig 1c). Similar IHC analysis of AR in 8 whole-mount (WM) CRPC sections (Supplementary Data 1) shows that 7 samples display the 3 AR patterns in the same specimen (Fig. 1c; Supplementary Fig. 1d) whereas 1 sample is largely AR^−/lo^. *These results reveal the AR heterogeneity in both expression levels and subcellular distribution patterns in patient CRPC*.

### Four xenograft CRPC models recapitulate the three AR patterns

Our laboratory has been using 4 AR^+^ PCa xenograft models, i.e., LAPC9, LAPC4, LNCaP, and VCaP^11,14–17^. Interestingly, when these 4 models transition from androgen-dependent (AD, i.e., androgen-sensitive) to a castration-resistant (CR; androgen-independent or AI) state during serial propagation in castrated mice (Fig. 1d; Methods), CR tumors display the same 3 AR immunostaining patterns observed in the clinical CRPC specimens (Fig. 1e; Supplementary Fig. 2). Specifically, IHC analysis using two antibodies against the N-terminus of AR reveals that CR LNCaP tumors show mainly nuc-AR^+/hi^, LAPC4 and VCaP nuc/cyto-AR, and LAPC9 AR^−/lo^ phenotypes (Fig. 1e; Supplementary Fig. 2). All 4 CR tumors stain negative for neuroendocrine (NE) markers synaptophysin and chromogranin A using monoclonal antibodies (Supplementary Table 1). As our AD→CR xenograft models induced by surgical castration and serial propagation in castrated mice mimic first-line clinical ADT using GnRH agonists^18,19^, we term the resultant CR tumors primary (1°) CRPC (Fig. 1d). As we shall present below (Fig. 1f), the 1° AR^+/hi^ and AR^−/lo^ CRPC xenografts display distinct responses to Enza.

**Fig. 2:**
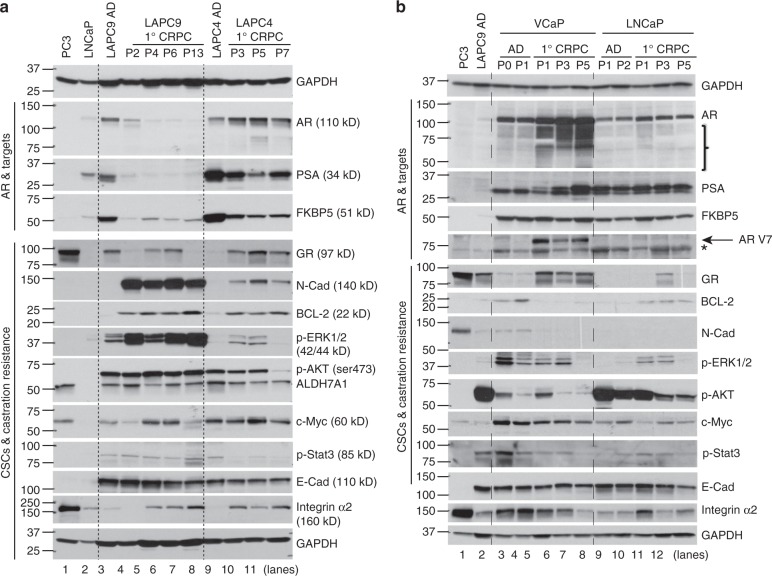
Four primary (1°) CRPC exhibit distinct changes in AR and other molecules. Four xenograft models were serially passaged in castrated male NOD/SCID (for LAPC9 and LAPC4; **a** or NOD/SCID-γ (NSG; for LNCaP and VCaP; **b** mice. The CRPC (passages indicated) and their corresponding AD tumors were harvested for WB analysis of indicated “AR and targets” and “CSCs and castration resistance” molecules (for AR, the clone 441 mouse mAb was used; Supplementary Table 1). PC3 cells were used as negative controls for AR. For each sample, whole cell lysate (60 μg) was loaded each lane, and GAPDH served as loading controls (top and bottom lanes). In a, no AR-V7 was detected in either LAPC9 or LAPC4 models. In b, the bands below AR indicated by a bracket are likely AR variants. *a non-specific band

Characterization of multiple proteins involved in AR signaling, cancer stem cells (CSC), and castration resistance in 1° CRPC reveal changes unique to each model (Fig. 2; Supplementary Table 2). In *LAPC9 CRPC*, castration leads to a prominent decrease/loss of AR and its two targets PSA and FKBP5 (Fig. 2a), and no AR splice variants including AR-V7 are observed in either AD or CR tumors. Among the “CSC and castration resistance” molecules examined, the LAPC9 CRPC upregulates N-Cadherin, BCL-2, p-ERK1/2, c-Myc, and integrin α2 (Fig. 2a; Supplementary Table 2). In contrast, GR (glucocorticoid receptor), p-AKT and ALDH7A1, p-Stat3 and E-Cadherin are similar or slightly reduced between AD and CR tumors (Fig. 2a; Supplementary Table 2). In the *LAPC4 model*, castration results in a slight increase in AR but decreases in PSA and FKBP5 (Fig. 2a). The LAPC4 1° CRPC upregulates GR, N-Cadherin, BCL-2, and integrin α2 but p-AKT, ALDH7A1, and p-Stat3 are decreased whereas p-ERK1/2 demonstrates transient increases (Fig. 2a; Supplementary Table 2). *VCaP 1° CRPC* shows increased AR, AR-V7, PSA, and GR but decreased BCL-2, N-Cadherin, p-ERK1/2, c-Myc and p-Stat3, whereas p-AKT and E-cadherin levels remain unchanged (Fig. 2b; Supplementary Table 2). In the *LNCaP model*, castration leads to increased AR but not AR splice variants including AR-V7 (Fig. 2b). N-Cadherin is not detected, GR shows only transient changes, and p-AKT and E-cadherin decrease slightly (Fig. 2b). pERK1/2 and integrin α2 increase then decline whereas BCL-2 and p-Stat3 demonstrate sustained increases (Fig. 2b; Supplementary Table 2). All 4 models of 1° CRPC do not express synaptophysin and chromogranin A. *Collectively, LNCaP, LAPC4, and VCaP 1° CRPC remain AR*^*+*^*and manifest changes associated with steroid receptor (AR, GR) signaling whereas the LAPC9 1° CRPC becomes AR*^−*/lo*^*and shows a prominent upregulation of CSC molecules*.

### The AR^−/lo^ LAPC9 CRPC resists Enza treatment de novo

When the 4 distinct 1° CRPC models are treated with the second-line antiandrogen Enza (Supplementary Fig. 1a), they show strikingly different Enza responses unique to each model (Fig. 1f). In the nuc-AR^+/hi^ LNCaP model, 1° CRPC is sensitive to Enza for up to ~7 weeks after which Enza-resistant tumors emerge (Fig. 1f). The nuc/cyto-AR LAPC4 and VCaP 1° CRPC, both of which grow more slowly than LNCaP 1° CRPC, initially respond to Enza but become Enza-resistant around 6.5 and 13 weeks, respectively (Fig. 1f). These therapeutic outcomes indicate that the AR^+^ LNCaP, VCaP, and LAPC4 1° CRPC models remain AR-dependent and Enza-sensitive but rapidly become Enza-resistant (i.e., secondary/2° CRPC). In stark contrast, the AR^−/lo^ LAPC9 1° CRPC (Fig. 1e, Fig. 2a; Supplementary Fig. 2) is Enza-resistant de novo (Fig. 1f). Notably, the Enza resistance observed in LAPC9 1° CRPC is not due to ‘intrinsic’ resistance of the LAPC9 model to ADT as the LAPC9 AD tumors are exquisitely castration-sensitive^11,20^. *These therapeutic experiments in CRPC models directly link AR expression levels to Enza sensitivity and predict that AR*^*−/lo*^*PCa cells present in patient tumors may also be Enza-resistant*.

### Establishing AR^+^ and AR-knockout LNCaP cell clones

As the above AR^+^ and AR^−/lo^ PCa xenograft models have different genetic backgrounds, we employ genome-editing technologies to generate genetically matched AR-tagged (AR^+^) and AR knockout (AR-KO) LNCaP clonal cells to compare their intrinsic biological and tumorigenic properties, as well as responses to castration and Enza. Briefly, we adopt the zinc-finger nuclease mediated integration to insert an RFP reporter into the endogenous *AR* locus and have generated AR^+^ (AR-RFP^+^) LNCaP clones (Supplementary Figs. 3-4; Supplementary Note 1; Methods). Meanwhile, we utilize the CRISPR-cas9 system to generate AR-KO LNCaP clones (Supplementary Fig. 5; Supplementary Note 1; Methods). The AR^+^ clones are positive for RFP (Supplementary Fig. 3e) and express high levels of nuclear AR protein in all cells (Supplementary Fig. 6a). siRNA-mediated AR knockdown leads to dramatically reduced RFP^+^ cells (Supplementary Fig. 4a), suggesting that RFP is co-expressed with endogenous AR and that the RFP signal reports AR expression. In contrast, the AR-KO LNCaP clones lack detectable AR expression by immunofluorescence (IF) (Supplementary Fig. 6b) and the ~110 kD full-length AR protein by western blot analysis (Supplementary Fig. 5d; Supplementary Fig. 6c). Consistently, quantitative RT-PCR (qPCR) analysis fails to detect *AR* mRNA expression in AR-KO LNCaP cells (Supplementary Fig. 6d). Two methods, i.e., AR target gene expression (Supplementary Fig. 6c-d) and luciferase reporter assays (Supplementary Fig. 6e-f) are utilized to compare AR activities in AR^+^ and AR-KO clones. qPCR analysis reveals no *PSA* mRNA expression in AR-KO clones (Supplementary Fig. 6c), and western blotting analysis shows undetectable levels of PSA protein and much reduced levels of NKX3.1 and TMPRSS2 in AR-KO compared to AR-RFP^+^ LNCaP cells (Supplementary Fig. 6d). To test the canonical ligand-activated AR activity, *ARE* and *PSA* luciferase reporter assays are performed in AR-KO and AR^+^ cells with and without 10 nM DHT (dihydrotestosterone) treatment. Lower baseline (i.e., in the absence of DHT), as well as DHT-stimulated reporter activities are observed in the AR-KO cells compared to AR^+^ cells (Supplementary Fig. 6e-f; Supplementary Note 1). Taken together, these results indicate that *AR-KO* LNCaP cells lack full-length AR protein expression and demonstrate much reduced target gene expression and blunted androgen responses.

### Differences in tumorigenicity in AR^+^ vs. AR-KO LNCaP cells

**Fig. 3:**
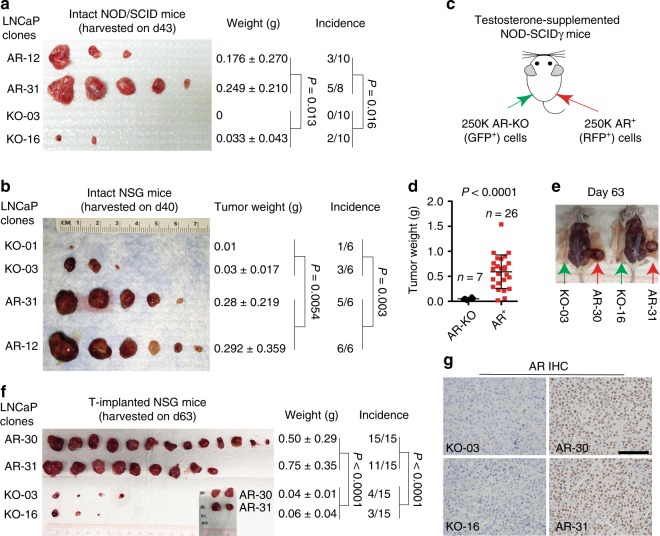
AR^+^ LNCaP cells possess higher tumor-regenerating capacities than AR-KO LNCaP cells in androgen-proficient hosts. **a**, **b** AR^+^ LNCaP cells demonstrate higher tumor-regenerating capabilities than AR-KO cells when implanted in intact NOD/SCID (**a**) or NSG (**b**) mice. Shown are clone numbers (500,000 cells for each clone per injection), endpoint tumor images, tumor weight and incidence, and *P* values for the pooled comparisons of tumor weight (Student’s *t*-test) and incidence (*χ*^2^ test). **c**–**f** AR^+^ LNCaP cells manifest much higher tumor-regenerating activities than AR-KO cells when implanted in the same T-supplemented male NSG mouse. Shown are the experimental scheme (**c**), comparison of tumor weights (**d**; Student’s *t*-test), images of two representative tumor-bearing mice (**e**), and endpoint tumor images (inset, 4 tumors from AR-30 and AR-31 clones harvested on d56 due to large tumor sizes) with clone numbers, tumor weight and incidence, and *P* values indicated for the pooled comparisons of tumor weight (Student’s *t*-test) and incidence (*χ*^2^ test) (**f**). **g** Representative AR IHC images of the end-point tumors. Scale bar: 250 μm. *P*-values for tumor weight and incidence comparisons were determined using unpaired Student’s *t*-test and Chi-squared test, respectively

Comparing the intrinsic biological properties, as well as tumor-regenerating potential in AR^+^ and AR-KO cells in different androgen environments, we find that AR^+^ LNCaP clones display higher clonal and sphere-forming potential in DHT-containing media compared to AR-KO clones (Supplementary Fig. 7a, b), which is associated with higher proliferation in AR^+^ cells (Supplementary Fig. 7c). Consequently, AR^+^ clones demonstrate higher tumorigenicity (i.e., generating more and larger tumors) than AR-KO clones in intact NOD/SCID (Fig. 3a) and NOD/SCID-γ (NSG) (Fig. 3b) mice. When AR^+^ and AR-KO clones are implanted side-by-side in the same NSG recipient mouse supplemented with exogenous T, AR^+^ LNCaP cells, strikingly, regenerate many more and significantly larger tumors than AR-KO cells (Fig. 3c–f; Student’s *t*-test for weight comparisons). AR^+^ cell-derived tumors all retain an AR^+^ phenotype (Fig. 3g). These side-by-side comparative studies in androgen-proficient conditions and hosts indicate that, as expected, AR^+^, but not AR-KO, PCa cells respond to androgen stimulation in vitro and in vivo. The results also imply that androgens (DHT) may preferentially suppress the clonogenic and tumorigenic capacities of AR-KO cells.

**Fig. 4:**
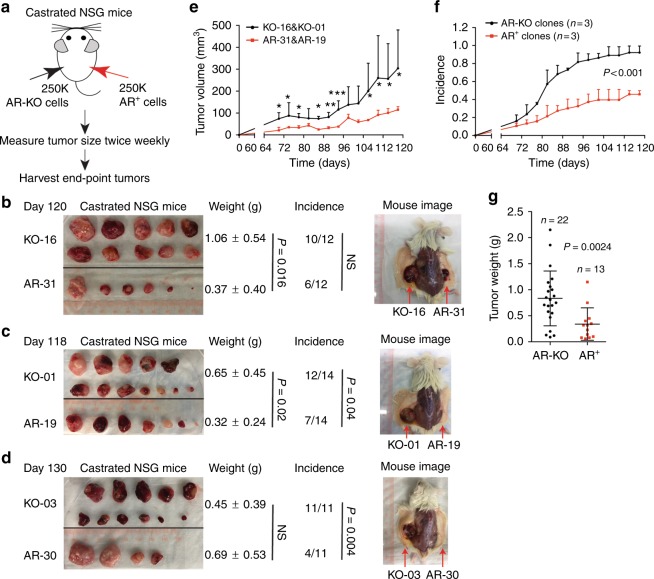
AR-KO PCa cells are highly tumorigenic in castrated mice. **a** Experimental design. Three ‘pairs’ of LNCaP clonal cells were used in tumor regeneration experiments: KO-16 and AR-31 (*n* = 12 mice; **b**); KO-01 and AR-19 (*n* = 14 mice; **c**); and KO-03 and AR-30 (*n* = 11 mice; **d**). **b**–**d** Images of endpoint tumors and tumor-bearing mice, and tumor weight and incidence with corresponding *P*-values. *P*-values for tumor weight and incidence comparisons were determined using unpaired Student’s *t*-test and *χ*^2^ test, respectively. NS not significant. **e** Higher growth rate of AR-KO tumors in castrated mice. Tumor volumes were measured twice weekly in castrated NSG mice bearing AR-KO (KO-16 and KO-01) and AR^+^ (AR-31 and AR-19) injections, starting from day 64 post implantation (mean ± SD). *P < 0.05; **P < 0.01; ***P < 0.001 Student’s *t*-test). **f** Tumor incidence in all 3 groups of animals starting from day 64 post implantation (mean ± SD). *P* < 0.01 for all time-point comparisons (Student’s *t*-test). **g** Graphical presentation of endpoint tumor weights in **b** and **c**. *P* value was determined by Student’s *t*-test (mean ± SD)

In sharp contrast, AR-KO LNCaP clones demonstrate higher tumor-regenerating capabilities than AR^+^ cells when implanted in castrated mice (Supplementary Fig. 8). Remarkably, when 3 clones each of AR^+^ and AR-KO LNCaP cells are implanted side-by-side in castrated NSG mice, AR-KO cells develop more and, frequently, larger tumors than AR^+^ cells (Fig. 4). These results corroborate that, as expected, the tumorigenic growth of AR^+^ PCa cells requires androgen. Importantly, these tumor experiments provide direct evidence that *the AR-KO PCa cells possess intrinsically high tumor-regenerating capabilities in androgen-ablated hosts*. As the AR^+^ and AR-KO clonal cells are implanted in the same castrated host, the results (Fig. 4) also provide the first hint that AR-KO cells possess a competitive advantage over AR^+^ tumor cells in regenerating tumors in the absence of androgen.

### AR-KO LNCaP cells resist Enza in vitro and in vivo

**Fig. 5:**
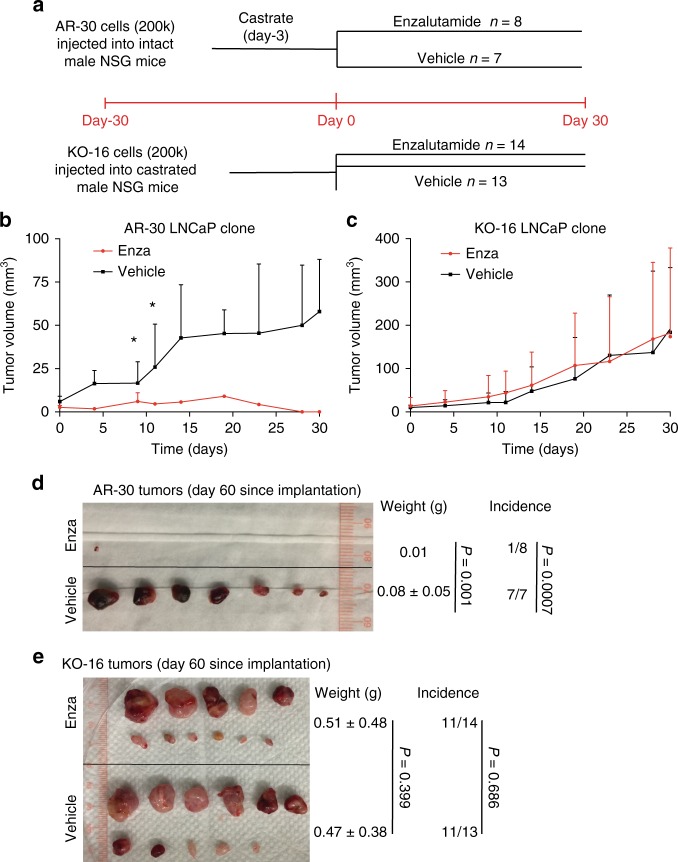
AR-KO LNCaP resist Enza in vivo. **a** Experimental scheme for in vivo Enza treatment (also see METHODS). Top: scheme for experiments using AR-30 LNCaP clones, in which animals were castrated 3 days before the start of Enza treatment (30 mg/kg/week, i.p). Bottom: scheme for experiments in which KO-16 cells were implanted in castrated NSG mice and Enza treatment was commenced 30 days thereafter. Both experiments were terminated on day 30 since the start of Enza treatment (i.e., day 60 since the tumor cell implantations). **b**, **c** Growth curves (by measuring tumor volumes) showing that AR^+^ LNCaP clones were sensitive (**b**) whereas AR-KO clones were resistant (**c**) to Enza. In b, *P < 0.01 (Student’s *t*-test; note that starting from the 4 time point, i.e., day 15, there was only one tumor remaining (see d) and therefore statistics could not be performed). **d**, **e** Endpoint tumor images showing that AR^+^ LNCaP cells are sensitive (**d**) whereas AR-KO cells are refractory (**e**) to Enza treatment in vivo. Also shown are tumor weight, incidence and *P* values (determined using unpaired Student’s *t*-test and *χ*^2^ test for tumor weight and incidence, respectively)

**Fig. 6:**
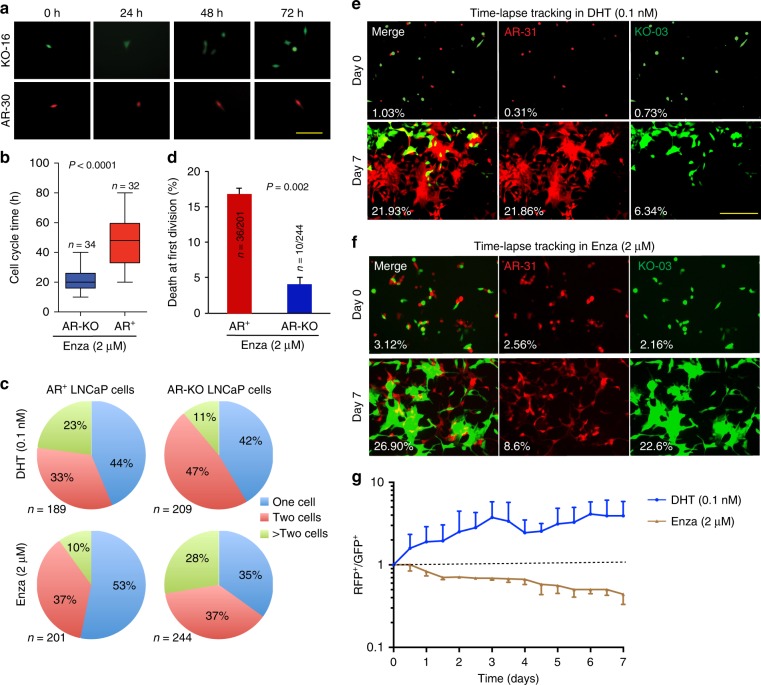
Unique biological properties and competitive advantages of AR-KO LNCaP cells in Enza-containing media. **a** Single AR-KO LNCaP cells divide faster and have shorter cell-cycle transit times than AR^+^ LNCaP cells in the presence of Enza as determined by time-lapse videomicroscopy. Representative images of a KO-16 cell (labeled with GFP, upper), which divided several times within 72 h and an AR-30 cell (lower), which did not divide within the same time interval. Scale bar: 250 μm. Corresponding movies were shown in Supplementary movies 1 and 2. **b** Graphical presentation of cell-cycle transition times in AR-KO vs. AR + LNCaP cells based on the time-lapse tracking of the 2 AR^+^ (AR-30 and AR-31) and 2 AR-KO (KO-03 and KO-16) LNCaP clones. The total number of cells analyzed and *P* value (Student’s *t*-test; mean ± SD) are indicated. Scale bar: 250 μm. **c** Single AR-KO LNCaP cells undergo more cell divisions than AR + clonal LNCaP cells in the presence of Enza (2 μM). Single AR^+^ (AR-30 and AR-31) and AR-KO (KO-03 and KO-16) cells in either DHT-containing (top) or Enza-containing (bottom) media were put under the JuLi Stage time-lapse videomicroscope and tracked for 4-5 continuous days with images taken every 2 h. Shown are pie chart presentations of endpoint clones that contained 1 cell, 2 cells, or >2 cells (3–6 cells) in each clone. Clones that contained >2 cells indicate that the starting single cell divided more than once during the recording period. The total number of single cells analyzed is indicated below (*n*). See Text for details. **d** High propensity of AR^+^ LNCaP cells undergoing death at the first cell division in the presence of Enza. As indicated, ~18% (36/201) of single AR^+^ LNCaP cells, compared to 4% (10/244) single AR-KO cells, were dead (*P* = 0.002, Student’s *t*-test, mean ± SD) during the first cell division when cultured in Enza. **e**, **f** Competitive advantage of AR^+^ cells in DHT-containing media and of AR-KO cells in Enza-containing media. RFP^+^ AR-31 LNCaP cells were mixed (1:1) with GFP-labeled KO-03 cells and plated in either DHT-containing (**a**) or Enza-containing (**b**) media at clonal density on day 0 and then tracked for 7 days. Shown are representative images. The percentages refer to relative confluence (fluorescence-positive area divided by the total area) determined by the JuLi Stage fluorescence time-lapse microscope. **g** Graphical presentation of competitive advantages of AR^+^ and AR-KO LNCaP cells in DHT-containing and Enza-containing media, respectively. Shown is the ratio of RFP^+^ (AR^+^) over GFP^+^ (AR-KO) LNCaP cells

The preceding comparative tumor studies have exposed striking *intrinsic* differences in tumor-regenerating properties in AR-expressing vs. AR-deficient PCa cells in relation to different androgen levels. We subsequently investigate the Enza response in the two cell types. AR-KO LNCaP cells display higher sphere-formation, clonal, and proliferative capacities than AR^+^ cells in Enza-containing media (Supplementary Fig. 9). As expected, AR^+^ LNCaP cell-derived tumors exhibit exquisite sensitivity to Enza whereas, in stark contrast, AR-KO cells develop tumors in castrated mice that are completely refractory to Enza treatment (Fig. 5). We perform single-cell tracking experiments by time-lapse videomicroscopy^11,14^ to further explore cell-intrinsic differences between AR^+^ and AR-KO PCa cells in response to Enza. The results reveal that AR-KO cells have shorter cell-cycle transit time, greater proliferative capacity, and lower apoptotic rate than AR^+^ LNCaP cells in Enza-containing media (Fig. 6a–d; Supplementary movies 1-4). Quantitative analysis of endpoint clones that contain 1 cell, 2 cells, or >2 cells (3–6 cells) in each clone (clones containing >2 cells indicate that the starting single cell has divided more than once during the recording period) demonstrates that in DHT media, 23% of the 189 single AR^+^ LNCaP cell-derived clones contain >2 cells compared to 11% of the 209 single AR-KO cells tracked (Fig. 6c), suggesting that AR^+^ LNCaP cells have a tendency to proliferate faster than AR-KO cells in androgen-stimulated conditions (consistent with earlier observations; Supplementary Fig. 7c). In contrast, in Enza-containing media, 28% (67/244) of the single AR-KO cells have divided more than once compared to 10% (20/201) of the AR^+^ cells (Fig. 6c; *P* = 0.05). Importantly, in Enza medium, 53% (107/201) of single AR^+^ LNCaP cells stay quiescent with no cell division (i.e., containing 1 cell in the endpoint clones) compared to 35% (85/244) of AR-KO cells in the same media (Fig. 6c; *P* = 0.01). Also, AR^+^ LNCaP cells show a high propensity to undergo death at the first cell division in the presence of Enza (Fig. 6d). These results indicate that AR^+^ LNCaP cells, at the single-cell level, are sensitive whereas AR-null cells are *intrinsically* resistant to Enza.

Of interest, when we compare the cell division profiles of AR-KO LNCaP cells in DHT vs. Enza, 28% cells undergo cell-cycle progression in Enza-containing media, which is significantly higher than 11% of the AR-KO cells in the presence of DHT (Fig. 6c; *P* < 0.01, *χ*^2^ test). These results again suggest that androgens may exert inhibitory effects on AR-KO cells.

### Distinct competitive advantages of AR^+^ vs. AR-KO PCa cells

Since most patient CRPC specimens contain both AR^+^ and AR^−/lo^ PCa cells (Fig. 1 a–c; Supplementary Fig. 1), we wonder whether these two PCa cell subpopulations may possess different competitiveness in different androgen/AR signaling environments. We perform competition experiments to test this possibility by mixing AR-RFP^+^ and GFP-labeled AR-KO LNCaP cells (1:1) and the results reveal a prominent competitive advantage for AR^+^ LNCaP cells in DHT and for AR-KO cells in Enza-containing media, respectively (Fig. 6e–g). Similar results are obtained using in vivo competition experiments (Supplementary Fig. 10). In intact (androgen-proficient) male mice the AR-RFP^+^ tumor cells predominate but in castrated mice the AR-KO (RFP^−^) tumor cells represent the majority in the tumors (Supplementary Fig. 10b). Notably, when several T-supplemented animals bearing mixed tumors are removed of T pellets and also castrated at week 4, AR-RFP^+^ LNCaP cells are reduced whereas AR-KO cells increased by week 9 (Supplementary Fig. 10b). These results corroborate apparent competitive survival and growth advantages of AR-KO PCa cells in androgen-depleted conditions.

### RNA-Seq identifies signaling pathways in AR^+/hi^ LNCaP CRPC

**Fig. 7:**
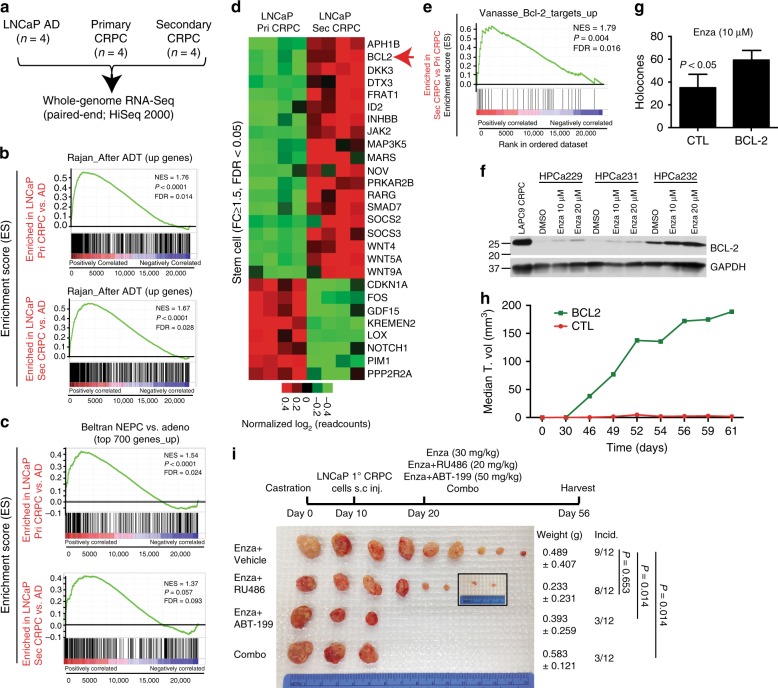
BCL-2 inhibitor prevents AR^+/hi^ LNCaP 2° CRPC. **a** Experimental scheme of RNA-Seq in LNCaP models. **b** GSEA showing that both primary (pri) and secondary (sec) LNCaP CRPC are enriched in gene sets upregulated in ADT-resistant patient CRPC^22^. All genes differentially expressed in 1° and 2° CRPC vs. AD tumors were used in GSEA against the upregulated genes in the Rajan data set^22^. **c** Both LNCaP 1° CRPC (vs. AD) and LNCaP 2° CRPC (vs. AD) DEGs positively associate with the CRPC-NE^24^ gene expression profile. All genes differentially expressed in 1° and 2° CRPC vs. AD tumors were used in GSEA against the entire list of genes expressed in the Beltran data set^24^. **d** Heat map of the stem cell associated genes altered in Enza-resistant LNCaP 2° (Sec) CRPC compared with LNCaP 1° (Pri) CRPC. BCL-2 was highlighted with a red arrow. **e** GSEA showing that genes preferentially expressed in LNCaP 2° CRPC (vs. 1° CRPC) were enriched in BCL-2 targets. **f** Enza induces BCL-2 expression in primary patient PCa cells. WB analysis of BCL-2 in HPCa cells freshly prepared from 3 primary tumors and treated with Enza for 72 h. **g** BCL-2 promotes LNCaP cell holoclone growth in Enza-containing medium. *P* value was determined using paired Student’s *t*-test (mean ± SD). **h** BCL-2 promotes rapid LNCaP AD tumor growth in intact male NSG mice. Presented is the median tumor (T) volume (note that the majority of tumors developed in the CTL group were very small (see Supplementary Fig. 15i) resulting in low median tumor volumes shown here). **i** BCL-2 is a critical therapeutic target for AR^+/hi^ LNCaP 2° CRPC. Shown on top is a timeline of the therapeutic experiment for LNCaP CRPC with 4 groups of treatment (all i.p injections; combo = Enza + RU486 + ABT-199). Shown below are endpoint tumors with weight and incidence indicated and *P* values for tumor incidence (*χ*^2^ test)

The above experiments in genetically matched LNCaP clonal cells have documented the relative proliferative, survival, and tumorigenic advantages of AR^+^ vs. AR^-^ PCa cells in androgen-proficient vs. androgen-ablated conditions, respectively. Although AR^+^ LNCaP tumors are sensitive to Enza, as observed in AR^+/hi^ LNCaP 1° CRPC (Fig. 1f) and patient CRPC, AR-KO LNCaP tumors, much like AR^−/lo^ LAPC9 CRPC (Fig. 1f), are refractory to Enza. Subsequently, we have made efforts to understand how AR^+/hi^ LNCaP 1° CRPC becomes Enza-resistant and why AR^−/lo^ LAPC9 1° CRPC is Enza-resistant de novo. Enza-resistant LNCaP 2° CRPC continues to express high levels of AR and also upregulates GR (Supplementary Fig. 11a-b; Supplementary Note 1), which has been implicated in castration resistance^21^. RNA-Seq analysis (Fig. 7a; Supplementary Fig. 11c–f) in LNCaP AD→1° CRPC→2° CRPC models (Fig. 1d) reveals 2,451 differentially expressed genes (DEGs; FC ≥ 2, FDR < 0.05) in LNCaP 1° CRPC (over AD), 3254 DEGs in 2° CRPC (over AD), and 601 DEGs in 2° CRPC compared to 1° CRPC (Supplementary Fig. 11f; Supplementary Data 2-4). Overall, there are more upregulated than downregulated genes in both 1° and 2° CRPC compared to LNCaP AD tumors (Supplementary Fig. 12a-b). Examination of 9 genes (*AR, NR3C1 [*GR*], KLK3, FKBP5, ALDH7A1, BCL-2, ITGA2, MYC*, and *STAT3*) in RNA-Seq (Supplementary Fig. 12), whose protein products are analyzed in western blotting analyses (Fig. 2b; Supplementary Fig. 11a), reveals that *AR* is upregulated by castration and further upregulated by Enza (Supplementary Fig. 12a, b, d) but *PSA* and *FKBP5* levels decrease in both CRPC (Supplementary Fig. 12d) whereas *NR3C1* is prominently upregulated in LNCaP 2° CRPC (Supplementary Fig. 12a-d). Among the 5 CSC molecules examined, *BCL-2*, *ITGA2*, and *MYC* show upregulation mainly in 2° CRPC whereas *ALDH7A1* and *STAT3* mRNA levels are decreased (Supplementary Fig. 12e). Notably, NEPC markers including *SYP*, *CHGA, CHGB, SCG3*, and *SCN3A* are either unaltered or actually downregulated in LNCaP CRPC.

Bioinformatics analysis of our RNA-Seq data (Supplementary Figs. 13–14; Supplementary Note 1), including IPA (Ingenuity Pathway Analysis) and GSEA (Gene Set Enrichment Analysis; Supplementary Table 3), reveals that both 1° and 2° LNCaP CRPC are enriched in gene signatures associated with ADT resistance in patient CRPC^22,23^ (Fig. 7b; Supplementary Fig. 13a) and patient CRPC-NE (neuroendocrine-like CRPC) phenotype^24^ (Fig. 7c). For example, GSEA (note that the FDR in GSEA is the estimated probability that a gene set with a given “normalized enrichment score/NES” represents a false positive finding, and an FDR < 0.25 for GSEA is considered statistically significant) shows that genes enriched in both 1° and 2° LNCaP CRPC correlate with the genes upregulated in patient tumors that have failed ADT^22^ (Fig. 7b), and, with the top 700 genes overexpressed in CRPC-NE compared to CRPC-Adeno tumors^24^ (Fig. 7c). Interestingly, both 1° and 2° LNCaP CRPC gene profiles are enriched for interesting signaling pathways including “Stem Cell Signaling”, “Lipid Signaling”, and “Neurogenesis” (Fig. 7d, Supplementary Fig. 13b–f, Supplementary Fig. 14a-c; Supplementary Note 1). For example, 1° and 2° LNCaP CRPC DEGs correlate with neurogenesis gene signatures in PSA^−/lo^ PCa stem cells^11^ (Supplementary Fig. 14a), normal human prostate basal/stem cells^25^ (Supplementary Fig. 14b), and patient CRPC-NE^24^ (Fig. 7c, Supplementary Fig. 14c). Annotation of the 601 genes preferentially expressed in 2° LNCaP CRPC involved specifically in Enza resistance reveals further enrichment in genes associated with stem cell and lipid signaling (Fig. 7d, Supplementary Fig. 14d–h).

### BCL-2 as a therapeutic target in AR^+/hi^ 2° LNCaP CRPC

Interestingly, BCL-2 is prominently upregulated at both mRNA (Fig. 7d, Supplementary Fig. 12b, e; Supplementary Fig. 15a) and protein (Fig. 2b, Supplementary Fig. 11a, 15b) levels in 1° and, in particular, 2° LNCaP CRPC. BCL-2 ‘target genes’ are also enriched in 2° LNCaP CRPC (Fig. 7e). Significantly, BCL-2, but not BCL-XL or MCL-1, is selectively upregulated in clinical CRPC^22^ (Supplementary Fig. 15c, d). The greater upregulation of BCL-2 in 2° LNCaP CRPC suggests that Enza may directly induce BCL-2 expression. Indeed, Enza treatment induces BCL-2 in LNCaP (Supplementary Fig. 15e), as well as primary patient tumor-derived PCa (Fig. 7f) cells. BCL-2 overexpression in LNCaP cells (Supplementary Fig. 15f) enhances CSC-enriched holoclone^11,14–17^ formation in regular (Supplementary Fig. 15g) and Enza-containing (Fig. 7g) media and also promotes tumor growth in intact (Fig. 7h; Supplementary Fig. 15h, i) and castrated (Supplementary Fig. 15j) mice. Consequently, we initiate a therapeutic experiment by treating 1° LNCaP CRPC with the BCL-2 inhibitor ABT-199^26^, which targets leukemic stem cells and has been recently FDA-approved (Venetoclax)^27^, either alone or with Enza. Remarkably, although ABT-199 alone does not inhibit the growth of 1° LNCaP CRPC (Supplementary Fig. 15k-l), combining ABT-199 with Enza, but not with GR antagonist RU486, greatly inhibits the emergence (incidence) of Enza-resistant 2° LNCaP CRPC (Fig. 7i). These results implicate BCL-2 as a critical driver and therapeutic target in Enza-resistant AR^+/hi^ CRPC and suggest that ABT-199 needs to be used in combination with Enza for the prevention and treatment of Enza-resistant AR^+/hi^CRPC.

### Signaling pathways and therapeutic strategies in AR^−/lo^ LAPC9 CRPC

**Fig. 8:**
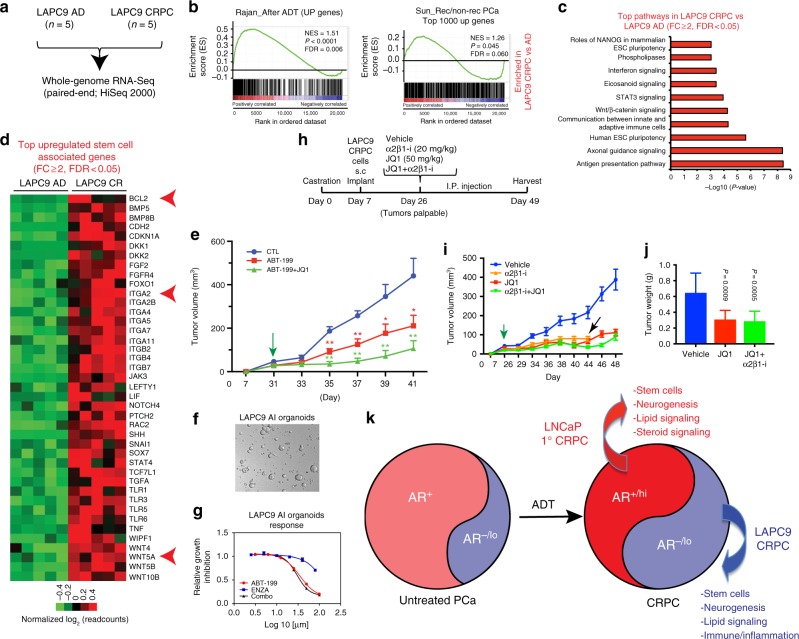
Novel mechanisms and therapeutic strategies in AR^−/lo^ LAPC9 CRPC. **a** Experimental scheme of RNA-Seq in LAPC9 models. **b** GSEA showing that LAPC9 CRPC is enriched in gene sets preferentially expressed in ADT-resistant patient CRPC^22^^,^^23^. **c** IPA biological function profiling of 3929 DEGs (FC ≥ 2, FDR < 0.05) in LAPC9 CRPC tumors (*n* = 5) relative to AD tumors (*n* = 5). Shown are selected top pathways. **d** Heat map showing representative stem cell genes upregulated in LAPC9 CRPC compared to AD tumors. Three representative genes are indicated by a red arrow. **e** Therapeutic targeting of AR^−/lo^ LAPC9 CRPC using ABT-199 alone or ABT-199 combined with JQ1 (see also Supplementary Fig. 19f). *P < 0.05; **P < 0.01 (Student’s *t*-test; mean ± SD). **f**, **g** BCL-2 inhibitor ABT-199 inhibits LAPC9 organoid growth. Shown are a representative phase contrast image of the LAPC9 CRPC (AI) organoids (**f**) and growth curves of LAPC9 organoids in response to treatments (**g**). Note identical responses to ABT-199 and ABT-199 plus Enza (**g**). **h**–**j** Targeting stem cell molecules integrin α2β1 and c-Myc inhibits LAPC9 CRPC growth. Shown are the experimental scheme (**h**), tumor growth curves (**i**) and endpoint tumor weights (**j**). In h, the doses of inhibitors for α2β1 (i.e., α2β1-i) and for BET bromodomain proteins including c-Myc (i.e., JQ1) are indicated. For i, the green arrow indicates the time (day 26) when drug treatment was initiated and the black arrow indicates the time when the α2β1-i ran out (and thus this treatment was stopped on day 44). The differences in tumor volumes between treatment groups vs. vehicle control group were all statistically significant (*P* < 0.01; Student’s *t*-test) starting from day 29. Error bars represent the mean ± S.D. For **j**, *P* values for tumor weights were determined by comparing with the vehicle control group (Student’s *t*-test; mean ± SD). **k** A model depicting dynamic changes in AR^+^ and AR^−/lo^ PCa cells in response to ADT (castration and anti-AR therapeutics). AR heterogeneity becomes accentuated in CRPC manifested by the emergence of AR^+/hi^ cells (illustrated by deeper red color) due to AR overexpression (e.g., genomic amplification of the *AR* gene and epigenetic alterations), as well as expansion of the AR^−/lo^ PCa cell population

The AR^-/lo^ LAPC9 CRPC does not respond to Enza (Fig. 1f). Both CR and Enza-treated LAPC9 tumors express little AR and PSA but high levels of CSC molecules including N-cadherin^28^, c-MYC, integrin α2, and BCL-2 (Fig. 2a; Supplementary Fig. 16a-b). RNA-Seq analysis in LAPC9 AD and CR tumors (Fig. 8a; Supplementary Fig. 16c-e) reveals 3,929 DEGs (FDR < 0.05; FC ≥ 2) in CRPC (Supplementary Fig. 17a; Supplementary Data 5). Examination of 4 differentiation-related genes (*AR, NR3C1, KLK3, FKBP5*) and 6 CSC genes *(ALDH7A1, BCL-2, CDH2, ITGA2, MYC*, and *STAT3*) in RNA-Seq (Supplementary Fig. 17b-d), for which we have assessed their protein levels during LAPC9 AD to CRPC transition (Fig. 2a; Supplementary Fig. 16a), reveals that, consistent with western blotting, *AR* and *FKBP5* are much reduced and *KLK3* is undetectable whereas *NR3C1* is slightly increased in LAPC9 CRPC (Supplementary Fig. 17c). Among the 6 CSC molecules, *BCL-2*, *CDH2*, *ITGA2*, and *STAT3* show (a trend of) upregulation in CRPC whereas *ALDH7A1* is decreased (Supplementary Fig. 17b,d). Interestingly, although the c-MYC protein levels are slightly elevated in LAPC9 CRPC compared to AD tumors (Fig. 2a; Supplementary Fig. 16a; Supplementary Table 3), its mRNA levels are actually reduced (Supplementary Fig. 17a, d), suggesting that the increase in c-MYC protein in LAPC9 CRPC might have resulted from post-transcriptional mechanisms.

GSEA demonstrates that LAPC9 CR tumors are enriched in genes associated with ADT resistance and tumor relapse in clinical CRPC (Fig. 8b). Additional bioinformatic analysis reveals gene expression patterns and deregulated signaling pathways both shared with AR^+/hi^ LNCaP CRPC and unique to AR^−/lo^ LAPC9 CRPC (Supplementary Fig. 18, Supplementary Fig. 19a-d). Like AR^+/hi^ LNCaP CRPC, LAPC9 CRPC is enriched in “Lipid Signaling” (Phospholipases, Eicosanoid Signaling, FXR/RXR Signaling, etc) and “Neurogenesis” (e.g., Axonal Guidance Signaling, Serotonin Degradation) pathways (Fig. 8c; Supplementary Fig. 18a). In support of a neurogenesis gene expression profile, LAPC9 CRPC-expressed genes are enriched in ‘Proneural’ subtypes of glioblastoma^29^ (Supplementary Fig. 18b) and in patient CRPC-NE^24^ showing an increased NE_score (Supplementary Fig. 19a, b). Of interest, LAPC9 CRPC and the patient CRPC-NE^24^ share 906 genes (Supplementary Fig. 19c, Supplementary Data 6), which are also enriched in Lipid Signaling and Neurogenesis pathways (Supplementary Fig. 19d). On the other hand, despite enrichment of neurogenesis genes in LAPC9 CRPC, the NEPC markers, including *SYP, CHGA, CHGB, SCG3*, and *SCN3A*, are either unaltered or actually downregulated in CRPC. Intriguingly, LAPC9 CRPC shows a unique enrichment in “Immune/Inflammation” related pathways including “Antigen Presentation Pathway”, “Adaptive Immune Cells”, and, particularly, “Interferon Signaling” (Fig. 8c; Supplementary Fig. 18a, c).

Consistent with our earlier biochemical characterizations (Fig. 2a), LAPC9 CRPC also displays prominent alterations in pathways that regulate stem cells, including “Human ESC Pluripotency”, “Wnt/β-catenin”, and “STAT3 Signaling” (Fig. 8c; Supplementary Fig. 18a). Among the top upregulated stem cell genes in LAPC9 CRPC are BCL-2, CDH2, integrins including ITGA2, SHH, and WNT genes such as WNT5A (Fig. 8c, d; Supplementary Fig. 17a, b, d; Supplementary Fig. 19e). CDH2 (N-cadherin) mRNA (Fig. 8d; Supplementary Fig. 17a, d) and protein (Fig. 2a; Supplementary Fig. 16a) are both upregulated in LAPC9 CRPC and a monoclonal antibody to N-cadherin has been shown to inhibit castration resistance^28^, suggesting that the CSC molecules upregulated in AR^−/lo^ CRPC are causally involved in CRPC emergence and progression. As LAPC9 CRPC is AR^−/lo^ and does not respond to Enza, we further explore potential causal functions of 3 upregulated CSC molecules, i.e., BCL-2, ITGA2 (integrin α2), and c-MYC by therapeutically targeting them alone or in combination. The BCL-2 inhibitor ABT-199 alone significantly inhibits LAPC9 CRPC growth (Fig. 8e; Supplementary Fig. 19f, Student’s *t*-test) and organoid expansion (Fig. 8f, g). Enza displays little inhibitory effects, and Enza plus ABT-199 demonstrates similar inhibitory effects to ABT-199 alone, on LAPC9 CRPC organoid growth (Fig. 8f, g). On the other hand, ABT-199 in combination with JQ1, which inhibits the BET bromodomain-containing proteins including c-MYC^30^, further inhibits LAPC9 CRPC growth in castrated mice (Fig. 8e; Supplementary Fig. 19f). Finally, as we have previously demonstrated the critical importance of integrin α2 in the tumorigenic growth of LAPC9 and LAPC4 CRPC in vivo^16^, we perform mono- and combinatorial therapies targeting integrin α2β1 using a small molecule inhibitor (α2β1-i)^31^. As shown in Fig. 8h–j. α2β1-i inhibits LAPC9 CRPC growth in castrated mice, which is potentiated by JQ1.

Collectively, these xenograft-based therapeutic experiments targeting 3 CSC signaling molecules, i.e., BCL-2, integrin α2β1, and c-MYC, offer novel proof-of-concept strategies for treating the AR^−/lo^ LAPC9-type of CRPC cells/clones.

## Discussion

Heterogeneity in AR expression levels in PCa has been noted for decades, from untreated tumors to CRPC to disseminated metastases^2–14^. In fact, AR^−/lo^ PCa cells pre-exist in untreated PCa^2,5,8,9,12–14^. By performing AR IHC analysis in prostatectomy specimens, we have observed ~5-20% of AR^−/lo^ PCa cells in a dozen of untreated Gleason 6-7 tumors with AR^+^ PCa cells representing the majority^14^. Heterogeneity in AR expression in CRPC tends to become accentuated such that AR^+^ PCa cells may evolve into AR^+/hi^ cells and the AR^-/lo^ cell population may expand^2,14^, leading to co-evolution of both subpopulation of PCa cells in the primary (prostate) tumor (Fig. 8k). PCa metastases, which are generally clonal in origin, may manifest as either AR^+^ or AR^−/lo^, as evidenced by commonly used, mostly metastasis-derived PCa cell lines (e.g., PC3, DU145, and PPC-1 being AR^-^ whereas LNCaP, LAPC9, VCaP, LAPC4 being AR^+^), as well as by recent positron emission tomography/computed tomography-based AR-axis imaging analysis^32,33^.

By analyzing 195 TMA cores and 8 whole-mount slides of CRPC specimens (mostly from the prostates with several metastases) obtained from 89 patients (most of whom were treated in the pre-Enza era), we have observed 3 distinct AR expression patterns, AR^+/hi^, nuc/cyto-AR, and AR^−/lo^, which can all be explained by well-established knowledge of steroid hormone receptor functions. Thus, under persistent selective pressure of castration and antiandrogens, PCa cells may overexpress (AR^+/hi^), lose (AR^−/lo^) or redistribute (cyto-AR) the target protein, AR, in order to survive. As more specific and potent AR-targeting therapeutics are being used in the clinic, more AR^+^ adenocarcinomas are being turned (reprogrammed) to AR^−/lo^ NE-like tumors called CRPC-NE^24,34^ or AR pathway-independent AR^−^NE^−^ tumors called double-negative PCa (or DNPC^35^). The emergence and increasing prevalence of AR^−/lo^ PCa cells/clones beg the important questions of whether they still respond to further antiandrogen treatments and how they can be therapeutically targeted.

The present study focuses on addressing the critical question of whether AR^+^ and AR^−/lo^ PCa cells possess intrinsically different biological and tumorigenic properties and how they respond to androgen-targeting therapeutics. Xenograft modeling of CRPC by serially propagating 4 AR^+^ human PCa models in castrated mice (which mimics first-line castration in patients using GnRH analogs^18,19^) reveals strikingly distinct evolutionary trajectories: the LNCaP model gradually becomes AR^+/hi^ and VCaP and LAPC4 become nuc/cyto-AR whereas the LAPC9 model evolves into AR^−/lo^. Therapeutic experiments demonstrate that the 3 AR-expressing CRPC models are transiently sensitive to Enza whereas the AR^−/lo^ LAPC9 CRPC are refractory to Enza treatment de novo. These results represent among the first *prospective* experimental evidence linking AR expression status to distinct castration and Enza responses. Ongoing studies are investigating how heterogeneity in AR distribution, e.g., largely cytoplasmic AR in LAPC4 CRPC, impacts tumor response to antiandrogens.

To further investigate intrinsic differences between AR-expressing and non-expressing PCa cells, we have established genetically matched AR^+^ and AR-KO LNCaP cell clones that allow us to perform side-by-side comparisons of their in vivo tumorigenic capabilities and relative competitive advantages in both androgen-intact and androgen-ablated (castration/Enza) hosts, as well as well-controlled cell biology experiments including single cell tracing studies. The results have demonstrated: (1) AR-KO cells possess intrinsically high tumor- and CRPC-regenerating activities in castrated mice (Fig. 4; Supplementary Fig. 8); (2) AR-KO PCa cells are resistant to Enza in vitro (Supplementary Fig. 9) and in vivo (Fig. 5) whereas AR^+^ cells are sensitive to Enza; (3) AR^+^ PCa cells, as expected, require androgen/AR signaling for their clonogenic and tumorigenic activities (Fig. 3; Supplementary Fig. 9) although some AR^+^ PCa cells may escape castration and regenerate tumors in castrated hosts (Fig. 3b–d; Supplementary Fig. 8); and (4) AR-KO LNCaP cells display prominent competitive advantages in castrated (or Enza-treated) conditions/hosts whereas AR^+^ PCa cells manifest competitive advantages in androgen-containing conditions (Fig. 6; Supplementary Fig. 10). While our manuscript was under revision, Bluemn et al. show that CRISPR/cas9-derived AR-null PacMet-UT1 PCa cells are also resistant to castration and Enza^35^.

Approximately 30% CRPC TMA cores are AR^−/lo^ (Fig. 1b) and 40-50% CRPC cells in whole-mount prostate sections are AR^−/lo^ (Fig. 1c; Supplementary Fig. 1d), which is substantially higher than the 5–20% AR^−/lo^ cells in untreated tumors^14^. Nevertheless, every treatment-failed prostate tumor harbors both AR^−/lo^ and AR^+/hi^ cells. This raises the question of why AR^−/lo^ PCa cells do not become the predominant or even the sole cell subpopulation in CRPC considering their competitive edges over the AR^+^ PCa cells. Plausible explanations may include: (1) incomplete chemical castration and AR blockage; (2) intracrine androgen production; (3) and inter-dependent symbiotic relationship between AR^+/hi^ and AR^−/lo^ cells. On the other hand, it is conceivable that in mCRPC patients, some metastatic foci may be predominantly AR^+/hi^ while others primarily AR^−/lo^ (reviewed in 2), as reported recently in patient metastases^32,33,35^.

Intriguingly, androgen seems to exert preferential suppressive effects on AR-deficient PCa cells. The two AR-KO LNCaP clones, when implanted in intact male mice with or without exogenous DHT, hardly regenerated any tumors (Fig. 3). Also, AR-KO cells, at the single-cell level, were less proliferative and formed fewer spheres and clones in DHT-containing media (Fig. 6c, e; Supplementary Fig. 7a--c). We have also observed that exogenous T greatly suppressed the growth of AR^−/lo^ LAPC9 CRPC implanted in castrated mice (Zhao et al., unpublished observations). Androgen suppression of AR^−/lo^ PCa cells have previously been reported, for example, in ARCaP cells^36^, and this effect seems to be still mediated through AR^37^. Interestingly, androgens have also been shown to repress AR^+/hi^ CRPC cells^38^. We are currently exploiting our novel AR-KO LNCaP and AR^−/lo^ LAPC9 CRPC models to elucidate molecular mechanisms underlying androgen suppression of AR-deficient PCa and to determine whether the phenomenon of T suppression of AR^−/lo^ CRPC in vivo might also involve host (e.g., immune, stromal) mechanisms. Also ongoing are comprehensive RNA-Seq and biological studies that aim to elucidate the transcriptome differences between AR^+^ vs. AR^−/lo^ (and AR-KO) PCa cells and to understand how some AR^+^ cells could develop tumors in castrated hosts.

Our studies suggest that AR^+^ and AR^−/lo^ PCa cell subpopulations, which co-exist in untreated primary tumors^2–14^, may co-evolve under pressure from castration/Enza to generate AR^+/hi^ (LNCaP-type), as well as AR^−/lo^ (LAPC9-type) CRPC cells/clones (Fig. 8k). RNA-Seq analyses show that AR^+/hi^ and AR^−/lo^ CRPC possess both shared and unique gene profiles—both are enriched in genes that regulate stem cells, neurogenesis, and lipid metabolism whereas AR^+/hi^ CRPC shows altered Steroid Signaling while AR^−/lo^ CRPC is enriched in Immune/Inflammation gene signatures (Fig. 8k). Although the functional significance of these pathways remains to be fully elucidated, several CSC molecules uncovered herein, highlighted by BCL-2, appear to be critical regulators and therapeutic targets of CRPC. Indeed, BCL-2 inhibition combined with Enza dramatically inhibits the emergence of Enza-resistant AR^+/hi^ CRPC whereas BCL-2 inhibition alone retards AR^−/lo^ CRPC. Although early studies have implicated BCL-2 in CRPC development^39–41^, our present study is the first to demonstrate that: (1) BCL-2, but not BCL-XL nor MCL-1, is exclusively upregulated in patient CRPC; (2) BCL-2 is directly induced by Enza treatment; and (3) BCL-2 is upregulated in both Enza-resistant AR^+/hi^ and Enza-insensitive AR^-/lo^ CRPC. These observations are consistent with early studies showing AR repression of BCL-2^41^. Although BCL-2 has been a long-standing therapeutic target in PCa, early clinical trials using non-specific and toxic BCL-2 family inhibitors have all failed. ABT-199 represents a third-generation BCL-2-slective inhibitor^26,42^, has recently been FDA-approved for CLL patients^27^, and is quickly moving to clinical trials for lymphoma due to its anti-CSC activities^43^. Collectively, our CRPC modeling in LNCaP system suggests a proof-of-concept therapeutic regimen for AR^+/hi^ CRPC clones, i.e., to combine steroid receptor (AR, GR) targeting therapeutics with inhibitors of ADT-induced CSC molecules such as BCL-2. Based on this rationale, we (G.C.; I.P.; D.G.T.) have initiated a phase Ib/II clinical trial of combining Enza and Venetoclax (i.e., ABT-199) to treat Enza-naïve metastatic PCa patients aiming to delay/prevent CRPC. It should be noted that the VCaP CRPC do not upregulate BCL-2, suggesting that, due to genetic diversity (e.g., TMPRSS-ERG fusion), different CRPC clones may employ different survival strategies to evade ADT.

Xenograft modeling in LAPC9 system has also provided proof-of-concept therapeutic strategies for AR^−/lo^ CRPC clones. The LAPC9 CRPC, which phenotypically resembles AR^−^NE^−^ DNPC^35^, is characterized by prominent upregulation of multiple stem cell/CSC molecules in addition to BCL-2 (Figs. 2a,  8c, d), suggesting that such CRPC cells/clones may be sensitive to, and, should be treated with, combinatorial anti-CSC strategies. In support, treating AR^−/lo^ LAPC9 CRPC with ABT-199 or ABT-199 plus JQ1 (a BET inhibitor that also inhibits c-Myc), led to dramatic inhibition of AI tumor growth (Fig. 8e). Similar CRPC-suppressive effects are also observed with an inhibitor of integrin α2β1, either alone or in combination with JQ1 (Fig. 8I, j). Of great interest, the AR^−/lo^ LAPC9 CRPC is greatly enriched in Immune/Inflammation gene signatures, in particular, the IFN signatures, and, the AR^−^ (NE^−^) patient CRPC are also enriched in IFN-responsive gene signatures^35^. These new observations raise the intriguing possibility that AR^-/lo^ CRPC cells might preferentially cross talk with the host immune system to escape immune surveillance.

Most prostate (primary) tumors in CRPC patients contain both AR^+/hi^ and AR^−/lo^ PCa cells and mCRPC patients harbor AR^+/hi^ and AR^−/lo^ metastatic clones^2,32–34^; therefore, curative anti-CRPC therapeutic strategies entail elimination of both PCa cell populations. Our observations that both AR^+/hi^ LNCaP and AR^−/lo^ LAPC9 CRPC are enriched in neurogenesis and lipid signaling gene signatures open the door for future exploration of novel regulators and therapeutic targets in CRPC. The availability of our novel CRPC xenograft and genetically matched AR^+^/AR-KO LNCaP clonal models should facilitate future studies on: (1) dynamic and reciprocal cooperation and interactions between the two PCa cell subpopulations during ADT and tumor relapse, (2) the causal functions of and novel signaling pathways in mediating Enza response by cytoplasmic AR (e.g., LAPC4 1° CRPC), and (3) PCa cell reprogramming and lineage plasticity accompanying tumor progression and clinical treatments. These models and tools should also facilitate unbiased high-throughput small-molecule and genetic screens to identify novel AR^-/lo^ CRPC cell-targeting therapeutics.

## Methods

### Overall study design and rationale

The present study was initiated to address the critical question of how AR heterogeneity in expression levels impacts PCa growth and responses to ADT and Enza. We started by performing IHC analysis of AR, using several titrated anti-AR N-terminal antibodies, in tissue microarray or whole-mount sections from a total of 89 CRPC patients. The results revealed 3 patterns of AR expression in patient CRPC: nuc-AR^+/hi^, nuc/cyto-AR, and AR^−/lo^ (Fig. 1a–c; Supplementary Fig. 1). Subsequent xenograft modeling and therapeutic experiments in 4 models indicated that nuc-AR^+/hi^ and nuc/cyto-AR PCa xenografts initially responded to Enza but this response was transient and Enza-resistant tumors quickly emerged. In contrast, AR^−/lo^ xenografts were Enza-resistant de novo (Fig. 1d–f). These results suggest that lack or low levels of AR expression confers castration/Enza resistance. To further investigate this important point, we employed two complimentary genome-editing techniques to derive AR-RFP^+^ (AR^+^) and AR-knockout (AR-KO) LNCaP clones, which were then used in a battery of in vitro biological and in vivo tumorigenic and Enza response assays, and the results revealed many interesting and surprising differences in the two subpopulations of PCa cells with different AR expression status (Figs. 3–6; Supplementary Fig. 3-10; Supplementary movies 1-4). We then focused on two xenograft model systems, i.e., Enza-resistant AR^+/hi^ LNCaP secondary CRPC and Enza-non-responsive AR^−/lo^ LAPC9 CRPC, performed RNA-Seq and exhaustive bioinformatics analysis, and conducted mechanistic and combinatorial therapeutic experiments (Figs. 7–8; Supplementary Figs. 11–19). The results revealed many novel signaling molecules and pathways in the development of two distinct modes of castration resistance mediated by AR^+/hi^ and AR^−/lo^ PCa cells, respectively. Importantly, our study establishes proof-of-principle therapeutic regimens that co-target the AR^+/hi^ and AR^−/lo^ CRPC cells/clones.

### Cell lines, animals, and reagents

LNCaP and VCaP cells were purchased from the American Type Culture Collection (ATCC) and cultured in RPMI (for LNCaP) or DMEM (for VCaP) plus 10% heat-inactivated fetal bovine serum (FBS) plus antibiotics. LAPC4 and LAPC9 xenograft lines were initially provided by Dr. Robert Reiter (UCLA) and have been used extensively in our previous studies^11,14–17^. These PCa lines were authenticated regularly in our institutional CCSG Cell Line Characterization Core and also examined to be free of mycoplasma contamination. Antibodies used in the current study are summarized in Supplementary Table 1. Immunodeficient mice, NOD/SCID (non-obese diabetic/severe combined immunodeficiency) and NOD/SCID-IL2Rγ^−/−^ (NSG) were obtained from the Jackson Laboratory, and the breeding colonies were maintained in standard conditions in our animal facilities. All animal-related studies in this study have been approved by our Institutional Animal Care and Use Committee (IACUC) at the M.D. Anderson Cancer Center (ACUF#00000923-RN00) or the Roswell Park Comprehensive Cancer Center (animal protocol# 1331 M and 1328 M). Therapeutic reagents used in this study included enzalutamide (also called MDV3100; APExBio, Cat# A3003 and Selleck Chemicals, Cat# S1250), ABT-199 (APExBio, Cat# A8194), Mifepristone/RU486 (APExBio, Cat# B1511), JQ1 (APExBio, Cat# A1910), docetaxel (APExBio, Cat# A4394), and TC-I 15 or compound 15 (an α2β1 integrin inhibitor or α2β1-i; Tocris Bioscience, Cat# 4527).

### CPRC TMAs and WM CRPC sections

Three TMAs containing 195 patient CRPC cores were originally derived from 81 CRPC patients (Supplementary Data 1): (1) UCLA TMA (*n* = 40 cores with 2 cores per patient, provided by Dr. Jiaoti Huang now at Duke University) was made from 20 CRPC patients^11,14^; (2) Cornell TMA (*n* = 99 cores with 3 cores per patient, provided by Dr. Mark Rubin and Dr. Himisha Beltran) was created from 33 CRPC patients; and (3) Roswell Park TMA (*n* = 56 cores with 2 cores per patient, provided by Dr. James Mohler) was acquired from 28 CRPC patients. In addition, WM slides from 8 CRPC patients were provided by Dr. Jiaoti Huang. Formalin-fixed paraffin-embedded (FFPE) sections were cut from these TMAs and WM samples and used for immunohistochemistry (IHC) of AR. Relevant information available on patient samples and treatment was summarized in Supplementary Data 1. All studies using archived human (tumor) specimens such as CRPC sections have been approved and followed the ethical regulations recommended by the Institutional Review Board (IRB STUDY00000079).

### IHC and IF in FFPE sections and western blotting

Basic IHC and IF procedures in FFPE sections (4 μm) were detailed previously^11,14–17^. For IHC, slides were deparaffinized in xylene and hydrated in gradient alcohols to water. Slides were treated with antigen retrieval in 10 mM citrate buffer (pH 6.0) and incubated with primary antibodies (Supplementary Table 1; optimal dilutions were determined by antibody titration experiments in pilot studies) followed by secondary antibodies and DAPI counterstaining (when applicable). IHC images were captured using an Olympus inverted (epifluorescence) microscope. The whole-mount and TMA IHC slides were scanned using Aperio ScanScope imaging system (Aperio Technologies, Vista, CA, USA) and a ×40 objective. Images were analyzed using the ScanScope software. For Western blotting analysis, whole cell lysate was prepared in RIPA buffer and run on 4–15% gradient SDS-PAGE gels. The proteins were transferred to nitrocellulose membrane followed by incubation with primary antibodies (Supplementary Table 1) and corresponding secondary antibodies. Films were developed using Western Lighting ECL Plus reagent (PerkinElmer). Representative original films for multiple Western blotting analyses were presented in Supplementary Figs. 20–25.

### Establishing serially transplantable AD and CR or AI PCa xenografts

Briefly, AD (i.e., androgen-sensitive) xenograft tumors, LNCaP, VCaP, LAPC4, and LAPC9, were routinely maintained in intact immunodeficient NOD/SCID or NSG mice^11,14–17^. To establish the CR lines, parental AD tumor cells were purified, mixed with Matrigel, injected subcutaneously and serially passaged in surgically castrated immunodeficient mice. The LAPC4 and LAPC9 AD and CR xenograft lines were maintained and passaged in NOD/SCID mice, whereas LNCaP and VCaP AD and CR xenograft lines were passaged in NSG mice. Xenograft tumors that became CR were termed primary (1°) CRPC and CR tumors that became Enza-resistant were termed secondary (2°) CRPC (Fig. 1d).

### Purification of human PCa cells from xenograft tumors

Basic xenograft tumor processing was described previously^11,14–17^. In brief, xenograft tumors were harvested, chopped into pieces (~1 mm^3^), and digested with Accutase (Sigma, A6964) for 30 min at room temperature (RT) under constant rotations. Single cells were collected via a pre-wetted 40-μm strainer and further purified on Histopaque-1077 (Sigma) gradient to deplete debris and dead cells.

### Primary human PCa (HPCa) sample processing

All primary HPCa samples were obtained with written informed patient consent in accordance with federal and institutional guidelines, which was approved by M.D. Anderson Cancer Center Institutional Review Board (IRB) (Protocol number LAB04-0498) and the Roswell Park Comprehensive Cancer Center IRB (BDR073416). Three HPCa samples were utilized in this study: HPCa229 (71 years old, Gleason grade 3 + 4 = 7, 30% tumor involvement), HPCa231 (72 years old, Gleason grade 4 + 4 = 8, 100% tumor involvement), and HPCa232 (72 years old, Gleason grade 4 + 3 = 7, 100% tumor involvement). Basic procedures for patient HPCa sample processing have been described previously^11,14^. Single HPCa epithelial cells were purified into Trop2^+^CD45^−^ cell population using flow cytometry (FACS), cultured in PureCol bovine collagen solution (Advanced BioMatrix) coated dishes^16^ in modified WIT medium^25^, and treated with DMSO (vehicle control) or Enza (10 or 20 μM).

### RNA isolation and qRT-PCR analysis

Total RNA was extracted using RNeasy Mini Kit (Qiagen) according to the manufacturer’s instructions. qRT-PCR was performed using a CFX Connect Real-Time PCR Detection System (Bio-Rad). qPCR data were normalized to 18 S RNA. Primers utilized were: 18 S, FWD: 5′-AAGTCCCTGCCCTTTGTACACA-3′ REV: 5′-GATCCGAGGGCCTCACTAAAC-3′; AR, FWD: 5′-TAGGGCTGGGAAGGGTCTAC-3′ REV: 5′-GGGAGGTGCTGCGCTC-3′; and PSA, FWD: 5′-GATGAAACAGGCTGTGCCG-3′ REV: 5′-CCTCACAGCTACCCACTGCA-3′.

### Generating AR-tagging LNCaP cell clones

The general scheme for generating AR-tagging LNCaP cell clones was presented in Supplementary Fig. 3a. The zinc finger nuclease (ZFN) pair (5′-ACCTGCTAATTAATCAAGTCACnnATGGTGAGCGTGGACTTTCCG-3′; Supplementary Fig. 3b) was designed and synthesized by Sigma (St. Louis, MO). Donor vector was synthesized by GeneScript (Piscataway, NJ), which contained the sequences of furin binding (cleavage) peptide, spacer, P2A peptide and tagRFP cDNA (Supplementary Fig. 3b; also see Supplementary Note 1). This backbone was flanked with 800 bp homologous arms, which are the sequences immediately upstream and downstream the *AR* stop codon (Supplementary Fig. 3b). To generate AR^+^ clones, 1 million LNCaP cells were transfected with 10 μg ZFN pair plasmid and 5 μg donor plasmid with Lipofectamine 2000. We used donor plasmid-only transfection as negative control. Three days after transfection, LNCaP single cell suspension was analyzed and sorted for tagRFP^+^ cells with BD FACSAria™ Fusion cell sorter. tagRFP^+^ cells were counted with hemocytometer after sorting and seeded into 96-well plates at one cell/well. Single cell-derived clones were propagated up in the conditioned medium prepared from regular LNCaP cell cultures containing 7% FBS, and collected after four weeks for genotyping. Genomic DNA of the clonal cells was prepared using the Qiagen Genomic DNA Extraction kit. Genotyping was determined using primers: P1 flanking the ZFN cutting site (FWD: 5′-GAAGGGGGAGGAAACAAAAG-3′, REV: 5’-TAGAGGAAATTCCCC AAGGC-3′) and P2 (designed inside the transgene tagRFP; FWD: 5′-ACTTCAAGTGCACATCCGAGG-3′, REV: 5′-AGTTTGCTAGGGAGGTCGC-3′) (Supplementary Fig. 3c). The upper band amplified with the P1 primers was purified from agarose gel and sequenced and the sequence data were blasted with Seqman Pro. Cell images were captured by an Olympus inverted epifluorescence microscope. The stable AR-RFP^+^ LNCaP clones were designated as AR-FRP^+^ cells.

### Generating AR-KO LNCaP cell clones

The general scheme for generating AR-KO LNCaP cell clones was presented in Supplementary Fig. 5a. Two guide RNAs (gRNAs) were designed targeting AR exon 1 (gRNA1 and gRNA2; Supplementary Fig. 5b) using the following website: http://crispr.mit.edu. The two gRNAs scored 59 and 97, respectively. We cloned the gRNAs into the CRISPR nuclease vector (ThermoFisher, A21174), and the reporter gene in the vector, OFP (orange fluorescence protein) immediately after the cas9 nuclease was used to screen for the transfected cells via FACS. Four microgram of total gRNA1 and gRNA2 vectors were transfected into 500,000 LNCaP cells with Lipofectamine 2000. Three days after transfection, LNCaP single cell suspension was analyzed and sorted for OFP^+^ cells with BD FACSAria™ Fusion cell sorter. OFP^+^ cells were counted with hemocytometer after sorting and seeded into 96-well plates at one cell/well. Clonal cells were collected after four weeks and genotyped using primers: ARN FWD: 5′-GAGACAGACTGTGAGCCTAGC-3′ and ARN REV: 5′-GCTGTGAAGGTTGCTGTTCC-3′. Sequence data were analyzed with Seqman Pro. The resultant LNCaP clones with AR knocked out were called AR-KO clones.

### Luciferase assays in AR^+^ and AR-KO cells

LNCaP clonal cells were seeded in 24-well plates (3 × 10^4^ cells/well) and were co-transfected with 1 μg ARE (androgen-responsive element) or PSA reporters together with the Renilla luciferase internal normalization plasmid (phRL-CMV). As a control, one group was treated with 10 nM DHT (dihydrotestosterone) 12 h before measuring luciferase activities. The ratio of firefly to Renilla luciferase activity was determined with a dual luciferase assay (Promega) 48 h later. Unpaired two-tailed Studentʼs *t*-test was used to compare differences between groups, and *P* < 0.05 was considered statistically significant.

### Clonal and clonogenic (sphere formation) assays in AR^+^ and AR-KO cells

For in vitro clonal assays, 5000 AR-KO or AR^+^ LNCaP cells were plated in 6-well plates, and 2 weeks later, cells were stained with 1:20 Giemsa and images scanned with an HP scanner. The number of clones was quantified with ImageJ. For clonogenic assays, 500 AR-KO or AR^+^ cells were suspended in 100 μL 50% Matrigel and 50% normal media containing 0.1 nM DHT or 2 μM Enza. The single cell suspension was spread on the edge of wells in a 24-well plate. 2 weeks later, images were taken with an Olympus inverted epifluorescence microscope and sphere numbers were counted.

### BrdU incorporation assays in AR^+^ and AR-KO cells

Cells at 50% confluence were incubated with 2 μM 5-Bromo-2’-deoxyuridine (BrdU) for 5 h. Cells were fixed with 4% PFA for 5 min and permeabilized with 1% Triton-X100. DNA was denatured using 3 M hydrochloric acid in 1% Triton-X100 for 20 min and then treated with 0.1 M sodium borate for 15 min. Slides were blocked with background sniper (Biocare Medical, Concord, CA, BS996) for 20 min and incubated with mouse monoclonal BrdU specific antibody (Sigma, MO, #B2531) for 5 h at room temperature, followed by incubation with goat anti-mouse IgG conjugated to Alexafluor 488 and counterstaining with DAPI.

### Tumor regeneration and therapeutic experiments in AR^+^ and AR-KO LNCaP clones

In general, 200,000 AR-KO and AR^+^ LNCaP cells were suspended in Matrigel (50:50 in normal media) and injected subcutaneously into castrated or T (testosterone)-implanted NSG male mice (8–10 weeks old). Tumor growth was measured twice weekly in two dimensions using a digital caliper. Tumor volume was calculated using 1/2 (length × width^2^). Animal body weight and health status were monitored during the experiments. At the end of experiments, tumors were harvested, and tumor incidence, weight, and gross images were recorded. For Enza therapeutic experiments (Fig. 5), 200,000 AR-KO cells were injected subcutaneously into castrated NSG male mice (~6 weeks old). At ~1 month later when tumors became palpable, mice were randomly divided into two groups: vehicle control (*n* = 14) and Enza (30 mg/kg/week, *n* = 13). In the mean time, 200,000 AR^+^ cells were injected subcutaneously into intact NSG male mice (~6 weeks old), which were castrated 1 week prior to initiation of Enza treatment at day 30, when animals were randomized into vehicle control (*n* = 8) and Enza (30 mg/kg/week and 3 times/week, *n* = 7) groups. Enza was dissolved in DMSO as 90 mg/ml stock solution, which was diluted in corn oil (Sigma) to make 2.5 mg/ml working solution. Mice were given 100 μL i.p. (intraperitoneal) injections 3 times a week.

### Time lapse videomicroscopy studies in AR^+^ and AR-KO LNCaP cells

LNCaP AR-KO and AR^+^ clonal cells were placed on the incubator stage of a JuLI™ Stage Real-Time microscope system, and maintained at 37 °C, 5% CO_2_ and >95% humidity in RPMI medium supplemented with 10% FBS, 1% Pen/Strep, and Enza (2 μM) or DHT (0.1 nM). Phase, RFP and GFP images were collected continuously with a ×20 objective lens at 2-h intervals for 96 h. Data analysis was performed using JuLI™ Stage Software.

### In vitro and in vivo competition assays in AR-KO and AR^+^ cells

For in vitro competition assays, AR-KO cells were infected with pGIPZ non-silencing GFP control lentivirus at an MOI of 10 to mark the cells with GFP. For in vitro competition assays, 5000 of AR-KO (GFP^+^) or AR^+^ (RFP^+^) cells were plated into one well in 6-well plates. Cell images were captured with JuLI™ Stage Real-Time microscope at 2-h intervals. Three points of each group were recorded and cell confluence data was measured using ImageJ by analyzing the proportion of fluorescence positive area of the cell images. For in vivo competition assays, 200,000 each of AR-KO and AR^+^ cells mixed together were injected subcutaneously into castrated or T-supplemented male mice. Once palpable, tumors were harvested at different time points and analyzed using a BD LSR II flow cytometer. A minimum of 10,000 cells were analyzed for each time point.

### RNA-Sequencing (RNA-Seq) and bioinformatic analysis

Basic procedures have been previously described^17,25,44^. In brief, LNCaP (AD, primary AI, secondary AI) and/or LAPC9 (AD and AI) tumors were harvested and total RNA was extracted by RNeasy mini kit (Qiagen), which includes on-column DNA digestion to deplete genomic DNA. 1000 ng of RNA was used to synthesize cDNA libraries using the Illumina TruSeq Stranded Total RNA LT Sample Prep kit according to the manufacturer’s guidelines. The libraries were then sequenced via 75 nucleotide paired-end running on HiSeq 2000 instrument (Illumina, San Diego, CA). 4 (for LNCaP) or 5 (for LAPC9) biological replicates were prepared for each condition. The reads were mapped to the reference human genome sequence (hg38) using TopHat version 2.0.10 (ref.^45^) and Bowtie version 2.1.0 (ref.^46^) with parameters “-r 50 --mate-std-dev 50 --library-type fr-unstranded –G”. More than 80% fragments were mapped to human genome. The number of fragments in each known gene from GENCODE Release 21 (ref.^47^) was enumerated using htseq-count from HTSeq package (version 0.6.1) (ref.^48^) with parameters “-f bam -m union -s reverse”. Genes with fewer than 10 fragments in all the samples were removed before differential expression analysis. There were a total of 60,119 genes, and after the removal of genes with low number of fragments, there remained 23,933 genes for LNCaP and 20,842 genes for LAPC9 models. DEseq (version 1.14.0) (ref.^49^) was used to call differentially expressed genes (DEGs) in our samples.

To define DEGs, we set up a stringent statistic cutoff of fold change (FC) of ≥2 and the false discovery rate (FDR) <0.05. A total of 2451 DEGs was identified between LNCaP AD and 1° CRPC and 3254 DEGs between LNCaP AD and LNCaP 2° CRPC. Also, 601 DEGs were identified between 2° CRPC vs. 1° CRPC using the criteria of FC ≥ 1.5 and FDR < 0.05, which were specific for Enza resistance. In LAPC9, 3929 DEGs were identified between AD tumors and CRPC. The Venn diagram was generated at http://bioinfogp.cnb.csic.es/tools/venny/ using lists of DEGs from two differential expression comparisons. Heatmaps were plotted by the free downloaded software of Cluster 3.0 and Java Tree View. To generate the landscape profile of RNA-Seq, for the fragments that have both ends mapped, the first reads were kept. Together with the reads from the fragments that have only one end mapped, every read was extended to its 3′ end by 200 bp in exon regions. For each read, a weight of 1/n was assigned, where n is the number of positions the read was mapped to. The sum of weights for all the reads that cover each genomic position was rescaled to normalize the total number of fragments to 1 M and averaged over 10 bp resolution. The normalized values were displayed using UCSC genome browser (http://genome.ucsc.edu/).

### IPA, GSEA, and determination of NE_scores

Ingenuity Pathway Analysis (IPA; Qiagen) was used to analyze pathways that are preferentially enriched in our LNCaP and LAPC9 CRPC models compared to the corresponding AD tumors using an IPA software downloaded from www.ingenuity.com. Basic procedures have been described in our earlier report^25^. In brief, we uploaded our DEG lists with FC ≥ 2 and FDR < 0.05 into the IPA software, and ran the Core Analysis to find the enriched signaling pathways. The FC and FDR were estimated by DESeq. IPA used Fisher’s exact test to determine if the genes in each pathway significantly overlap our DEG lists.

We performed GSEA (Gene Set Enrichment Analysis)^50^ to correlate our LNCaP and LAPC9 CRPC models (vs. AD tumors) to known gene expression signatures. The normalized count table estimated by DESeq for all genes (i.e., unselected) was taken as the input for GSEA and the genes were ranked in GSEA by the Signal/Noise metric. The sample permutation was performed by 1000 times to assess the statistical significance. We followed the GSEA user’s guide (http://www.broadinstitute.org/gsea/doc/GSEA UserGuideFrame.html) using default parameters to run the software. The FDR in GSEA is the estimated probability that a gene set with a given “normalized enrichment score/NES” represents a false positive finding, and an FDR < 0.25 for GSEA is considered statistically significant. For GSEA, our genes (all genes in LNCaP primary or secondary CRPC vs. AD or LAPC9 CRPC vs. AD) are used as the ranked gene list (all the genes ranked from most upregulated to downregulated) to interrogate GSEA database.

The gene expression-profiling data sets employed to perform GSEA of our RNA-Seq were summarized in Supplementary Table 3, and included: (1) an RNA-Seq data set comparing the transcriptomes of PCa patient tumors before vs. after ADT^22^ (Figs. 7b,  8b); (2) a microarray data set comparing the gene expression profiles of ‘androgen-independent/AI’ patient tumors vs. untreated ‘AD’ patient tumors^23^ (Supplementary Fig. 13a; note that we used the top 1000 upregulated genes (AI vs. AD) from this microarray data set^51^); (3) an RNA-Seq analysis comparing AD LNCaP vs. androgen-independent LNCaP-AI cells^52^ (Supplementary Fig. 13a); (4) a microarray analysis comparing the gene expression profiles of recurrent (Rec) vs. non-recurrent (non-Rec) PCa^53^ (Fig. 8b; note that we used the top 1000 upregulated genes (Rec vs. non-Rec) from this microarray data set^51^); (5) an in-house RNA-Seq data set comparing the PSA^−/lo^ vs. PSA^+^ LNCaP cells (Supplementary Fig. 14a; Liu, X. et al., manuscript in preparation); (6) an RNA-Seq data set in human benign prostate basal/stem cells vs. luminal cells^25^ (Supplementary Fig. 14b; Supplementary Fig. 18c); (7) an RNA-Seq profiling data set in neuroendocrine prostate cancer (CRPC-NE) vs. CRPC adenocarcinoma (CRPC-Adeno)^24^ (Fig. 7c; Supplementary Fig. 19a); (8) a microarray data set of genes that are upregulated in the neural crest stem cells^54^ (Supplementary Fig. 13f); and (9) a microarray data set presenting genes preferentially expressed in the proneural subtype of glioblastoma mutiforme^29^ (Supplementary Fig. 13f; Supplementary Fig. 18d).

In addition, we used curated gene set C2 of the Molecular Signature Database (MSigDB) version 6.0 to determine correlations between our gene set and gene sets in the MSigDB. Representative data sets presented were also summarized in Supplementary Table 3. For example, Vanasee et al.^55^ identified gene signatures upregulated in primary B lymphocytes isolated from transgenic mice overexpressing Bcl-2 (Fig. 7e). Boquest et al.^56^ performed microarray analysis and uncovered genes upregulated in stromal stem cells (CD31^+^) vs. non-stem cells (CD31^−^) from adipose tissue (Supplementary Fig. 14e). In another study, Lim et al.^57^ compared gene expression changes of 4 normal subpopulations of mammary epithelial cells isolated from mouse mammary gland (stem cell, committed luminal progenitor, mature luminal, and stromal cells) with their human counterparts, and showed genes upregulated in the stem cell subpopulation (Supplementary Fig. 14e). Ben-Porath et al.^58^ analyzed the gene enrichment patterns related to embryonic stem cells from different human tumor types, and found that the Polycomb-regulated genes were preferentially repressed in poorly differentiated tumors (Supplementary Fig. 14f). Through a Hu6800 GENECHIP array in HT1080 cells after treatment with interferon (IFN)-α, IFN-β, or IFN-γ, Der et al.^59^ identified genes upregulated upon INF-α, IFN-β, or IFN-γ stimulation (Supplementary Fig. 18b).

An NE_score was developed using two different methods to determine the similarities in gene expression profiles between adenocarcinomas and NEPC^24,60^. The first method^24^ is based on a list of 70 genes and the score was the Pearson correlation coefficient of normalized FPKM values of each sample with the mean value of the NEPC samples in the paper. The score is called integrated NEPC score. The second method^60^ is based on a smaller list of 50 genes and the NE_score was the weighted sum of the FPKM value of those 50 genes. The raw data were obtained from Beltran et al.^24^. We determined the relative NE_scores in the LNCaP and LAPC9 CRPC vs. AD using the second method by Lee et al.^60^. Briefly, the list of 50 genes was selected to best distinguish NEPC and ADPC samples from Beltran et al^24^ using a logistic regression model and elastic net regularization. The weighted sum of the 50 genes was treated as NE_score and results were presented as relative NE_scores in AD tumors vs. CRPC (Supplementary Fig. 14c and 19b).

### Therapeutic experiments in CR xenografts

NOD/SCID or NSG male mice (8–10 weeks old) were castrated on day 0. After 7–10 days, 100,000–500,000 CRPC cells in different models were injected subcutaneously in Matrigel. For Enza treatment (Fig. 1f), once tumors were palpable, castrated mice were randomly divided into two groups (vehicle control, *n* = 10; Enza, 30 mg/kg, *n* = 10) and drugs were administered intraperitoneally 3 times a week till the end. For targeted therapeutic experiments in AR^+/hi^ LNCaP-type of CRPC (Fig. 7i), drugs were delivered as follows: (1) Enza (*n* = 12, 30 mg/kg); (2) Enza (30 mg/kg) + RU486/Mifepristone (20 mg/kg, i.p. 5 times per week^61^) (*n* = 12); (3) Enza (30 mg/kg) + ABT-199 (50 mg/kg, oral gavage, 5 times per week^26^) (*n* = 12); (4) Combo: Enza (30 mg/kg) + RU486 (20 mg/kg) + ABT-199 (50 mg/kg) (*n* = 12). For experiments in *AR*^*-/lo*^*LAPC9-type of CRPC* (Fig. 8e–j; Supplementary Fig. 19f), drugs were delivered as follows: (1) vehicle control (*n* = 12); (2) JQ1 (50 mg/kg, i.p, 5 times per week)^30^ (*n* = 10); (3) α2β1 inhibitor (i.e., α2β1-i)/compound 15 (20 mg/kg, i.p, 4 times per week^31,62^) (*n* = 12); (4) Combo: JQ1 (50 mg/kg) + α2β1-i (20 mg/kg) (*n* = 12). For additional experiments in AR^-/lo^ LAPC9 CRPC, once tumors grew up, castrated mice were randomly divided and drugs were delivered as follows: (1) vehicle control (*n* = 12); (2) ABT-199 (50 mg/kg) (*n* = 12); (3) Combo: JQ1 (50 mg/kg) + ABT-199 (50 mg/kg) (*n* = 12). ABT-199 (5 mg/ml) was dissolved in ethanol, and prepared in Phosal-50 PG (Lipoid): polyethylene glycol-400 (Spectrum): ethanol (6:3:1)^26^, whereas other drugs were dissolved in 3–5% DMSO or ethanol and prepared in corn oil (Sigma). In another experiment, castrated NSG males bearing LNCaP 1° CRPC were treated with ABT-199 (100 mg/kg, oral gavage, 5×/week) or vehicle (5% DMSO, 50% PEG30, and 5% Tween 80). Tumor growth was measured weekly in two dimensions using a caliper and tumor volumes were determined using 1/2 (length × width^2^). Animal body weights and health status were monitored. At the end, tumors were harvested, and tumor incidence, weight, and gross images were recorded.

### BCL-2 related experiments

*To overexpress BCL-2 in LNCaP cells*, we first produced the Precision LentiORF BCL-2 lentivirus in 293 T packaging cells (ATCC), which was titered using GFP positivity in HT1080 cells. LNCaP cells were infected at MOI 10–15 for 72 h at 37 °C. Precision LentiORF Bcl-2 lentiviral vectors (Clone ID: PLOHS_100004100) and the control vector (Precision LentiORF RFP positive control) were purchased from GE Dharmacon. The overexpression effect was assessed using Western blotting and GAPDH was used as a loading control. *For clonal assays*, BCL-2 overexpressing LNCaP cells were plated at a clonal density (5,000 cells/well in a 6-well dish) under both regular serum-containing (RPMI-1640 medium + 10% FBS + 1% penicillin/streptomycin) and AR-inhibited (RPMI-1640 medium + 10% FBS + 1% penicillin/streptomycin + 10 μM Enza) conditions. Holoclones^11,14^ were counted and imaged 2 weeks after plating. *For tumor assays*, control and BCL-2 overexpressing LNCaP cells were mixed with Matrigel and injected s.c. at 100,000 cells/injection in intact NSG mice (8–10 weeks). Tumor volume was measured at weekly basis and the median tumor volume was shown in Fig. 7h. Tumor incidence was monitored weekly. At the endpoint, tumors were harvested, and tumor weight and incidence were summarized. *To examine if BCL-2 can promote tumorigenicity in castrated hosts*, tumor cells were obtained from AD tumors and injected into castrated NSG mice at 20,000 cells/injection.

### ‘Therapeutic’ experiments in LAPC9 CRPC organoids

LAPC9 CRPC (AI) xenografts were digested to single cells using Accumax^11,14–17^ and single cells were obtained using a 40-μm cell strainer and purified using Histopaque-1077 (Sigma, 10771). Cells were plated at a density of 25,000 cells/cm^2^ in a 384 well plate. Cells were plated in 5% Matrigel diluted with organoid media^63^ without androgens. Three days after plating, organoids were treated with various doses of Enza, ABT-199, or an equimolar combination of the 2 drugs in a constant-ratio combination. Cell viability was analyzed 6 days post treatment using CellTiter-Blue Cell Viability Assay (Promega).

### Statistical analysis

Unpaired two-tailed Student’s *t*-test was used to compare significance in tumor weight and tumor volume, and *χ*^2^ test was employed to compare tumor incidence. The results were presented as mean ± S.D. *P* < 0.05 was considered statistically significant.

## Electronic supplementary material

Supplementary Information

Description of Additional Supplementary Files

Supplementary Data 1

Supplementary Data 2

Supplementary Data 3

Supplementary Data 4

Supplementary Data 5

Supplementary Data 6

Supplementary Movie 1

Supplementary Movie 2

Supplementary Movie 3

Supplementary Movie 4

## Data Availability

The RNA-Seq data is deposited in the Gene Expression Omnibus (GEO) database and can be accessed at https://www.ncbi.nlm.nih.gov/geo/query/acc.cgi?acc = GSE88752.
