# Supplementary material for: Linking prostate cancer cell AR heterogeneity to distinct castration and enzalutamide responses
Source: Nat Commun. 2018 Sep 6;9:3600. doi: 10.1038/s41467-018-06067-7 (PMC6127155; doi:10.1038/s41467-018-06067-7)
Supplement: Supplementary file 1 — Supplementary Information [file 41467_2018_6067_MOESM1_ESM.pdf]

Supplementary Note 1

Generation of AR$^+$ and AR-KO LNCaP cell clones

AR-tagged LNCaP clonal cells (Supplementary Fig. 3a) are generated by inserting the reporter, tagRFP, into the C-terminus of *AR* gene using the zinc finger nuclease (ZFN) mediated integration strategy (Supplementary Fig. 3b). A ZFN pair (5'-ACCTGCTAATTAATCAAGTCACnnATGGTGA GCGTGGACTTTCCG-3') is selected for introducing double strand breaks due to its high cutting efficiency. Meanwhile, donor constructs containing the reporter tagRFP are designed for homology-directed repair (HDR; Supplementary Fig. 3b). P2A peptide (ATNFSLLKQAGDVEENPGP) is used to link AR and tagRFP. The P2A peptide is optimized for transgene expression by adding furin cleavage site (RAKR) and spacer (SV-SGSG) peptides. Two sets of primers, P1, flanking the sequences around the ZFN cutting sites and P2, located inside the tagRFP gene (Supplementary Fig. 3c), are used for genotyping. A total of 46 AR-RFP$^+$ LNCaP clones (Supplementary Fig. 3a) are established. Genotyping with P1 primers shows that all RFP$^+$ clones harbor the ~1.2 kb band from integrated allele and one smaller band from wild-type allele. Genotyping with P2 primers also shows the expected 614 bp band (Supplementary Fig. 3c). Sequencing of the upper P1 band in several clones indicates successful integration of the transgene (Supplementary Fig. 3d). Under fluorescence microscope, virtually all cells in the AR-RFP$^+$ LNCaP cell clones are RFP$^+$ (Supplementary Fig. 3e). When AR-RFP$^+$ clones are treated with AR siRNA, RFP is lost within 48 h (Supplementary Fig. 4a), suggesting that RFP is co-expressed with, and, reports endogenous AR. Western blotting with tagRFP antibody shows no AR bands in AR$^+$ clones (Supplementary Fig. 4b), indicating full cleavage of tagRFP from the AR at the protein level.

We also employ CRISPR-cas9 technology to generate AR knockout (AR-KO) LNCaP cell clones (Supplementary Fig. 5a). The two gRNAs used, gRNA1 and gRNA2, are predicted to target the *AR* gene a short distance downstream the start codon (Supplementary Fig. 5b). Six AR-KO clones, i.e., KO-01, KO-03, KO-14, KO-16, KO-21 and KO-41, are established, which harbor various mutations in both alleles

(Supplementary Fig. 5c) and lack full-length AR protein expression as assessed by Western blotting using two anti-AR antibodies (Supplementary Fig. 5d).

The characterization studies (Supplementary Fig. 3–6) together indicate that 1) the AR-tagged LNCaP clones express normal functional AR, and 2) AR-KO clones demonstrate undetectable AR mRNA and protein expression, low AR activity, and blunted androgen responses. The slightly increased luciferase activities observed in AR-KO LNCaP cells upon DHT stimulation (Supplementary Fig. 6e-f) could be due to activation of related steroid receptors such as GR by DHT and low levels of other steroids in serum.

STR-based cell authentication experiments demonstrate that the derived AR^{+} and AR-KO LNCaP clones were identical to the parental LNCaP cells.

LNCaP 2° CRPC upregulated AR and GR

During AD ➔1° CRPC transition in the LNCaP model, nuc-AR is upregulated (Fig. 1e; Supplementary Fig. 2a). When LNCaP 1° CRPC is treated with Enza, tumors initially respond for ~6–7 weeks and then become Enza-resistant (Fig. 1f). Western blotting analysis of molecules involved in AR signaling, CSC, and castration resistance in Enza-resistant LNCaP 2° CRPC reveals that AR is further upregulated but the two canonical AR targets, PSA and FKBP5, are downregulated (Supplementary Fig. 11a-b), which suggested that these tumors are enriched in phenotypically undifferentiated ($PSA^{-/lo}$) cells, as previously reported[11,14]. One significant change in LNCaP 2° CRPC is the upregulation of GR (Supplementary Fig. 11a-b), which has been implicated in castration and Enza resistance[21]. Lower M.W. species of both AR and GR are noted (Supplementary Fig. 11a, asterisks), which might represent spliced isoforms although AR-V7 is not detected (Supplementary Fig. 11a). Among the "CSCs & castration resistance" molecules examined, BCL-2, Myc, p-Akt and E-cadherin are upregulated whereas N-cadherin is not detected and integrin α2, p-ERK1/2, and p-Stat3 do not show changes in 2° LNCaP CRPC (Supplementary Fig. 11a; Supplementary Table 2).

Signaling pathways in $AR^{+/hi}$ LNCaP 1° and 2° CRPC

Comprehensive bioinformatics analysis of our RNA-Seq data in LNCaP CRPC models is performed (Fig. 7b-e; Supplementary Fig. 12-14). GSEA (Gene Set Enrichment Analysis; Supplementary Table 3) reveals that genes preferentially expressed in the LNCaP 1° and 2° CRPC (versus AD tumors) are enriched in gene signatures associated with ADT resistance in patient CRPC[22,23] and androgen-independent LNCaP cells (Supplementary Fig. 13a). IPA (Ingenuity Pathway Analysis) of the 2,451 and 3,254 DEGs expressed in 1° and 2° LNCaP CRPC, coupled with GSEA, demonstrates that LNCaP 1° and 2° CRPC also are enriched in signaling pathways including "Stem Cell Signaling", "Lipid Signaling", and "Neurogenesis". The *Stem Cell Signaling pathway* includes GO terms such as HIF1α, BMP, Notch, Human ESC Pluripotency, TGFβ, and STAT3 (Supplementary Fig. 13, b and d). The *Lipid Signaling pathway* encompasses GO terms including, among others, Eicosanoid Signaling, Phospholipases, Sphingosine and Sphingosine-1-phosphate Metabolism, and Phospholipase C Signaling (Supplementary Fig. 13, b and d). Both 1° and 2° LNCaP CRPC show upregulation (and also some downregulation) of numerous phospholipases and molecules involved in the metabolism of arachidonic acid, sphingolipids, and other lipids (Supplementary Fig. 13, c and e). Finally, the *Neurogenesis pathway* is highlighted by GO terms such as Axonal Guidance and Neural Stem Cell Specification (Supplementary Fig. 13, b and d). Indeed, both 1° and 2° LNCaP CRPC gene expression profiles are enriched in gene sets preferentially expressed in neural crest stem cells and proneural glioblastoma (Supplementary Fig. 13f), CSC-enriched PSA$^{-/lo}$ PCa cell population in which neurogenesis genes are over-represented[11,14] (Supplementary Fig. 14a), neurogenesis gene-enriched normal human prostate basal/stem cells[25] (Supplementary Fig. 14b), and CRPC-NE (CRPC with neuroendocrine-phenotype) tumors[24] (Fig. 7c). In further support of the neurogenesis gene enrichment, both 1° and 2° LNCaP CRPC show increased NE_scores (Supplementary Fig. 14c).

The 601 DEGs in LNCaP 2° CRPC compared to 1° CRPC (FC≥1.5, FDR< 0.05; Supplementary Data 4), may play important roles in Enza resistance. IPA of the 601 DEGs demonstrates that the most prominently altered pathways are related to stem cell functions, which include 'STAT3 Signaling', 'IGF-1 Signaling', 'Wnt/β-catenin Signaling', 'TGF-β Signaling', and 'Notch signaling' (Supplementary Fig. 14d). Many stem cell regulators (e.g., WNT) are upregulated in 2° CRPC (Fig. 7d) and GSEA reveals enrichment

of stem cell expression and stem cell signaling signatures in LNCaP 2° CRPC vs. 1° CRPC (Supplementary Fig. 14e-f). The "Lipid Signaling" pathways (GO terms including Eicosanoid Signaling, TR/RXR Signaling, Prostanoid Biosynthesis, Phospholipases, etc) continue to be significantly enriched in the LNCaP 2° CRPC (Supplementary Fig. 14g-h). The functional significance of these signaling pathways in regulating castration and Enza resistance in $AR^{+/hi}$ PCa cells awaits experimental validation.

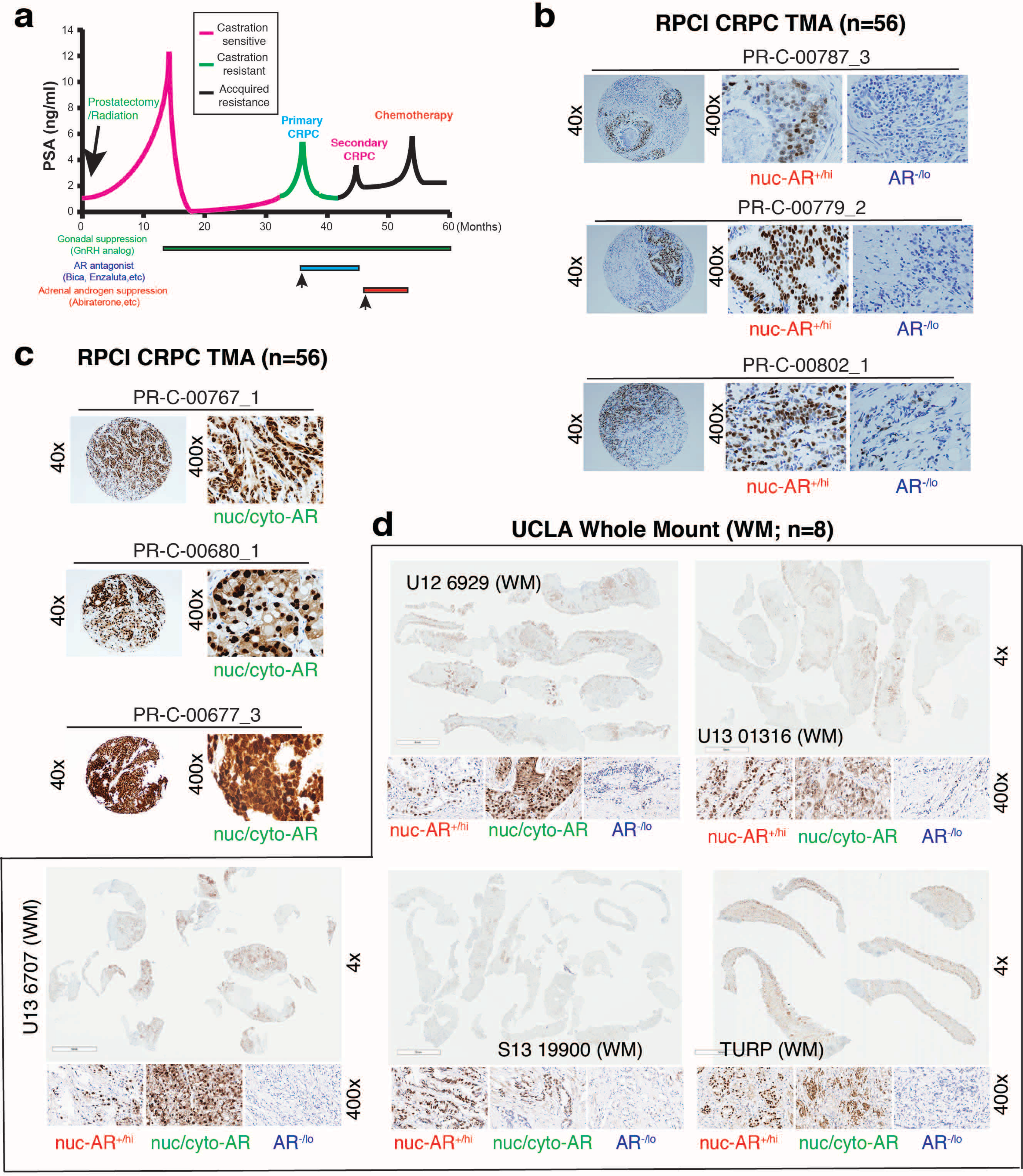

Li & Deng et al., Supplementary Figure 1

Supplementary Figure 1. Heterogeneous AR protein expression in patient CRPC specimens.

(a) *Schematic showing current clinical treatment for advanced and metastatic PCa*. See Introduction for more discussions.

(b–d) *AR heterogeneity in CRPC*. AR IHC in RPCI TMA illustrating 3 CRPC samples containing both nuc-$AR^{+/hi}$ as well as $AR^{-/lo}$ PCa cells (b) and 3 CRPC cores containing nuc/cyto-AR^{+} cells (c). Presented in d is AR IHC in WM sections from 5 CRPC patients showing 3 AR expression patterns.

Original magnifications are presented in individual panels.

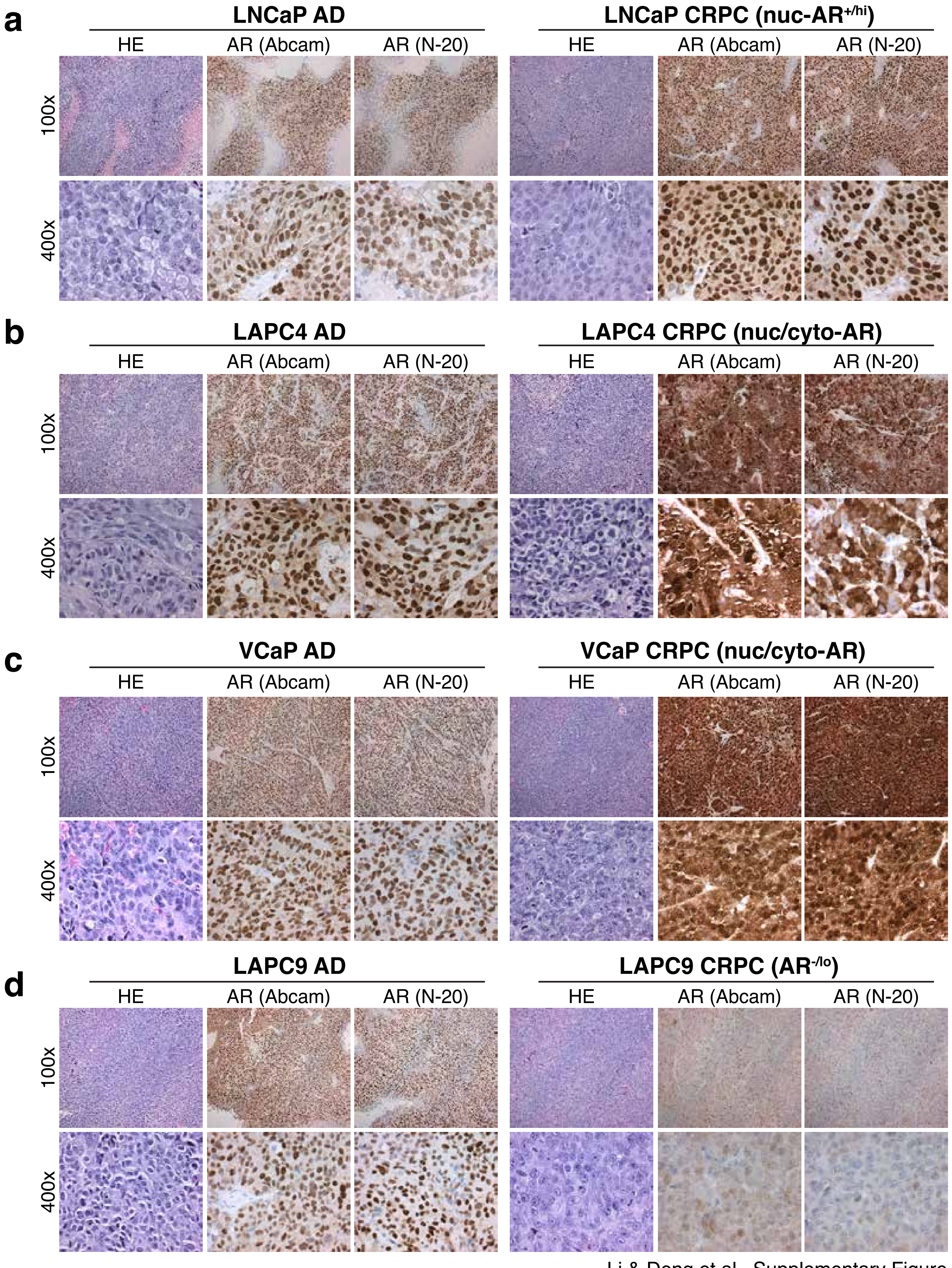

Li & Deng et al., Supplementary Figure 2

Supplementary Figure 2. Distinct AR expression and distribution patterns in 4 CRPC models.

Shown are representative images of AR IHC staining using 2 N-terminally directed anti-AR antibodies (Supplementary Table 1) in 4 pairs of androgen-dependent (AD) tumors and corresponding CRPC. Original magnifications (100X and 400X) are indicated.

(**a**). AR IHC analysis shows increased nuclear AR staining in LNCaP primary CRPC compared to the corresponding AD tumors.

(**b**). AR IHC analysis shows redistribution of nuclear AR in LAPC4 AD tumors to primarily cytoplasmic AR in the LAPC4 primary CRPC.

(**c**). AR IHC analysis shows redistribution of nuclear AR in VCaP AD tumors to cytoplasmic/nuclear AR in the VCaP primary CRPC.

(**d**). AR IHC analysis shows prominent reduction and loss of AR in LAPC9 CRPC compared to mainly nuclear AR in LAPC9 AD tumors.

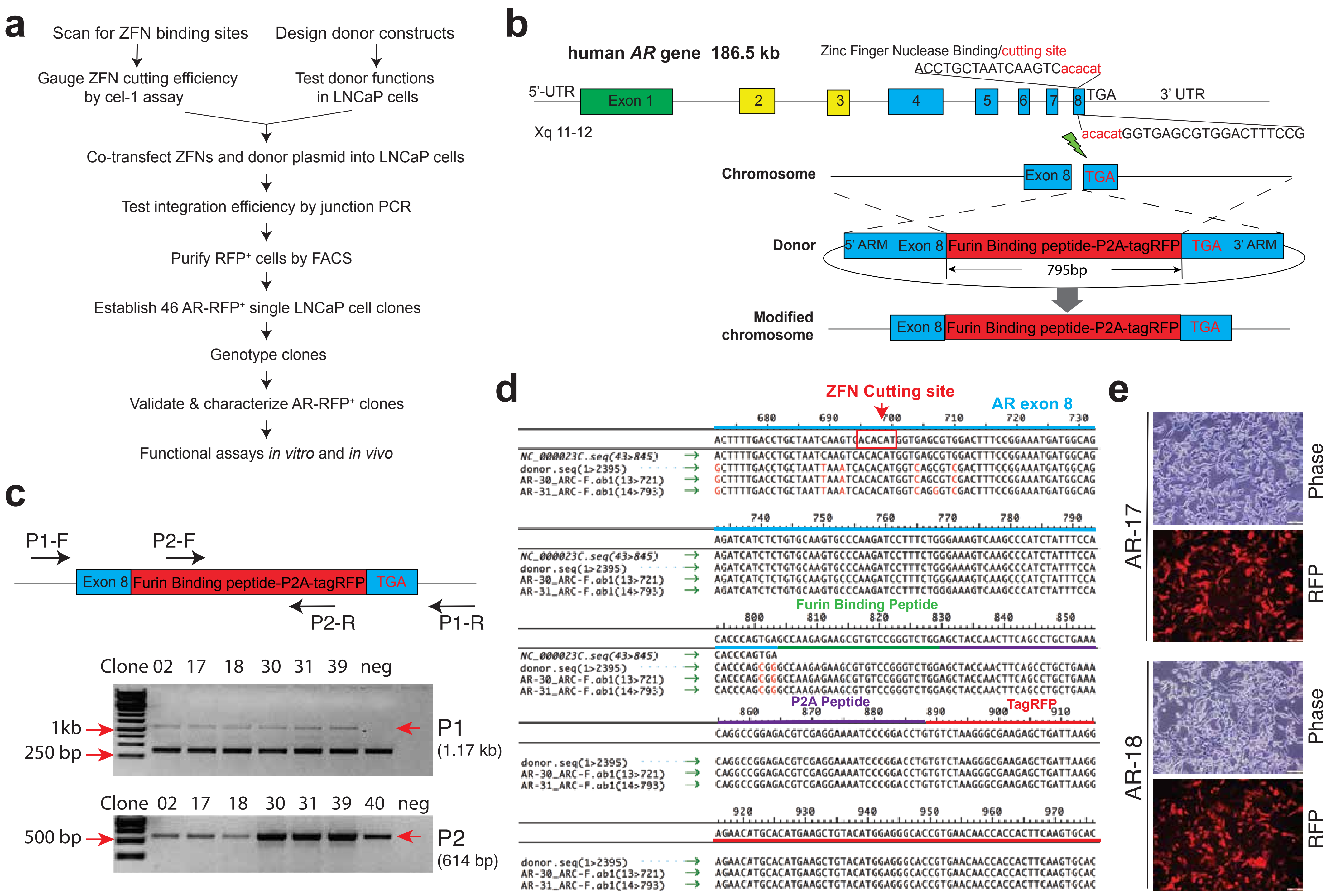

Li & Deng et al., Supplementary Figure 3

Supplementary Figure 3. Generating AR-tagging (AR^{+}) LNCaP cell clones using ZFN-mediated integration strategy.

(a) Workflow of the strategies used to generate AR-tagging LNCaP clones (see Supplementary Note 1).

(b) Schematic of the human *AR* gene locus and exon-intron structure, donor plasmid used to introduce tagRFP, and the expected integration product. The ZFN cutting site is indicated in red on the top and the donor structure in the middle. The tagRFP is knocked into the C terminus of human *AR* gene before the TGA stop codon.

(c) Genotyping of representative AR-RFP clones. Genomic DNA extracted from clones indicated was amplified with primer P1 (flanking the inserted gene tagRFP), and primer P2 (inside tagRFP-containing cassette). F and R refer forward and reverse primers. PCR products were electrophoresed on 1% agarose gel with ethidium bromide. Red arrows on the right indicate the integrated alleles. 'neg' indicates a (negative) non-RFP^{+} LNCaP clone similarly generated.

(d) DNA sequences of the upper bands amplified with the primer P1 (C) blasted with the NCBI human AR sequence. From top to bottom are NCBI *AR* sequence, donor sequence, and sequences of the clone AR-30 and AR-31. Shown are also ZFN cutting site (red arrow), the AR exon 8 (blue bar), furin-binding peptide (green bar), P2A peptide (purple bar), and tagRFP (red bar).

(e) Representative phase and red fluorescence images of clones AR-17 and AR-18 (scale bars in the images, 20 µm) showing virtually all cells in the clones were RFP^{+}.

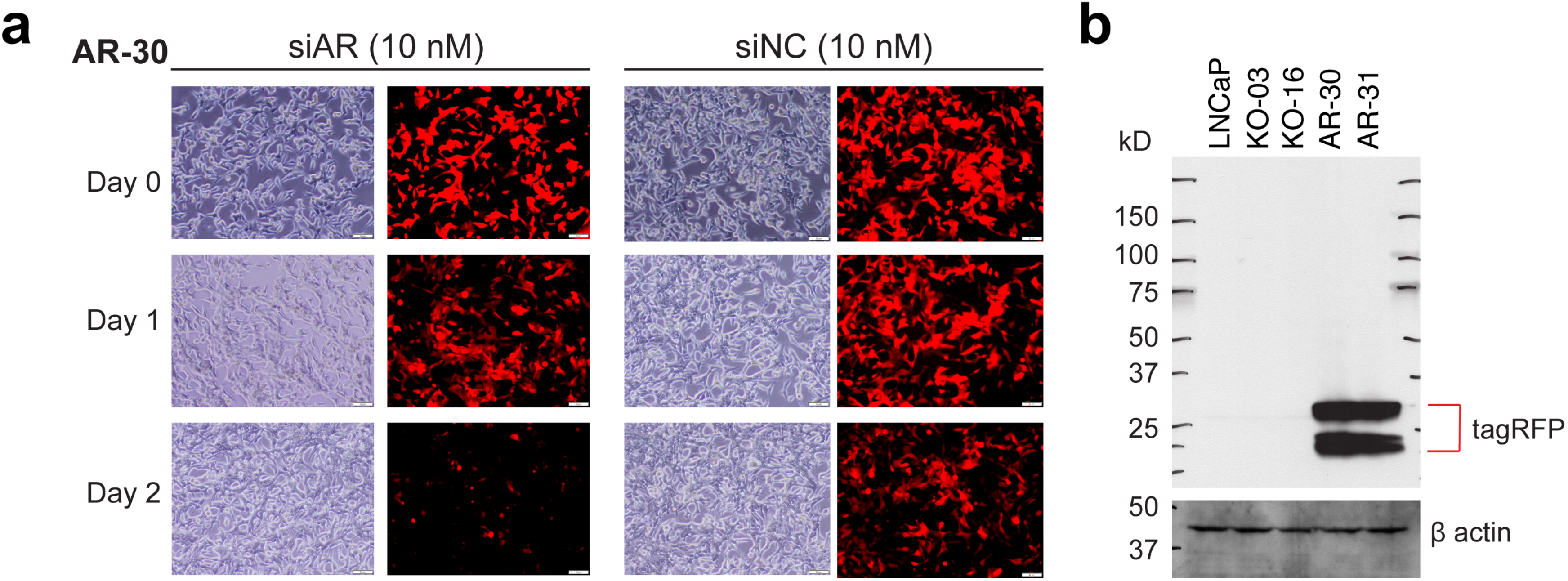

Supplementary Figure 4. Further characterizations of RFP-tagged AR⁺ LNCaP cell clones.

(**a**) RFP reports endogenous AR as assessed using siRNA knockdown (KD) of AR. AR-30 clonal LNCaP cells were transfected with control or AR siRNAs (10 nM). Shown are the phase and RFP images at time 0 and day 1 – 2 after siRNA transfection.

(**b**) WB analysis of tagRFP in the indicated AR⁺ and AR-KO LNCaP clones demonstrated that tagRFP and AR were separate protein products.

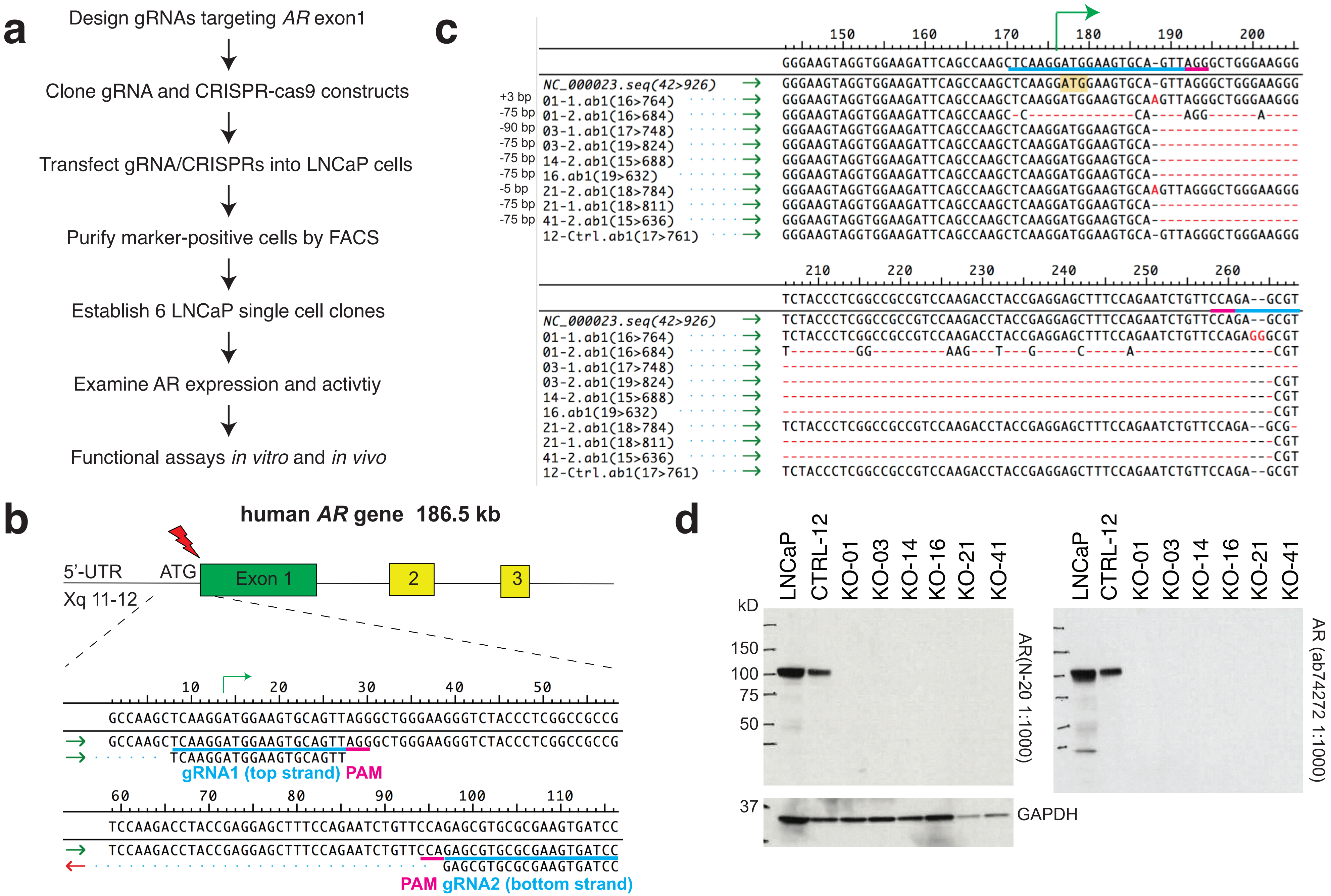

Supplementary Figure 5. Generating AR-KO LNCaP cell clones with CRISPR-cas9.

(**a**) Workflow of generating AR-KO LNCaP cell clones.

(**b**) Schematic of the two gRNAs (gRNA1 and gRNA2, blue bars) used in inducing double strand breaks (DSBs) in the 5' of human AR gene. PAM sequences were marked in pink.

(**c**) DNA sequences of the indicated AR-KO clones (i.e., KO-01, KO-03, KO-14, KO-16, KO-21, and KO-41) blasted with the NCBI human AR gene (NC_000023). The mutations in these clones were presented on the left. A wild-type LNCaP clone (CTRL-12) was included as control.

(**d**) WB analysis using two anti-N-terminus AR antibodies showed AR KO in the 6 clones. CTRL-12 was a wild-type LNCaP clone.

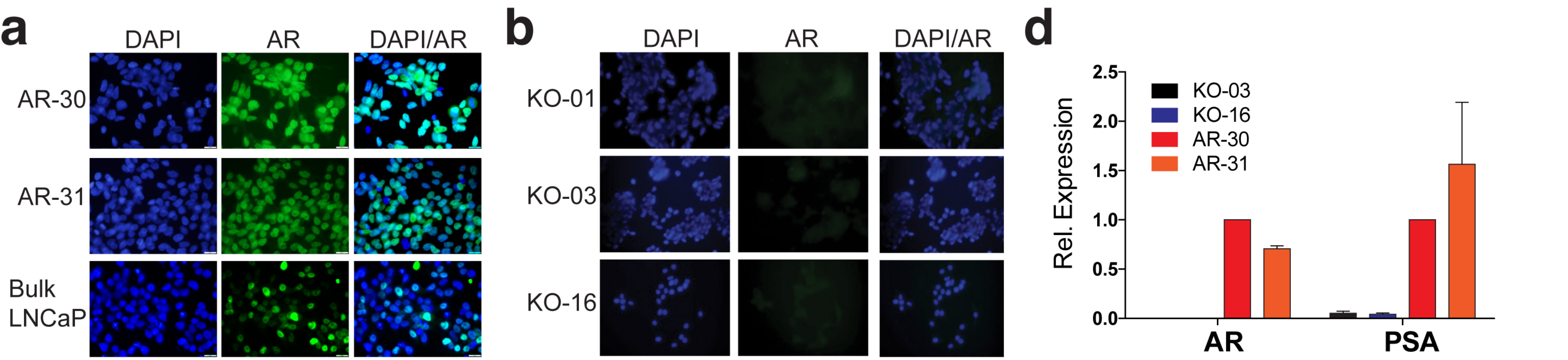

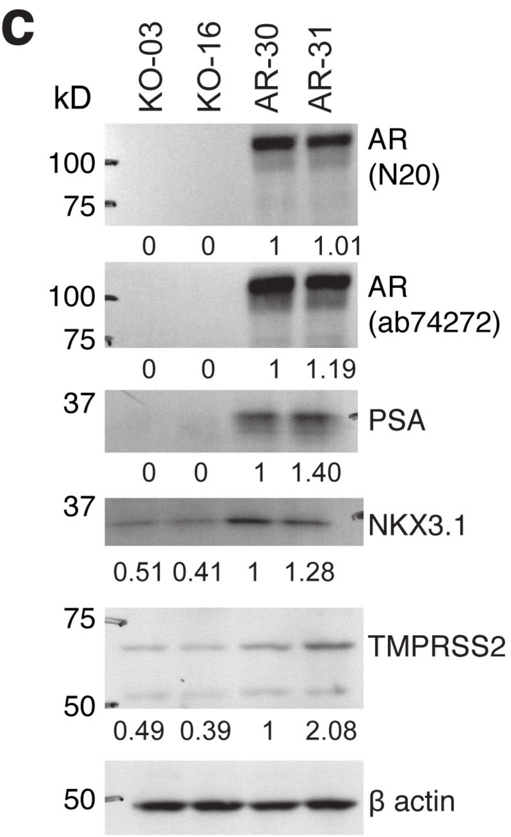

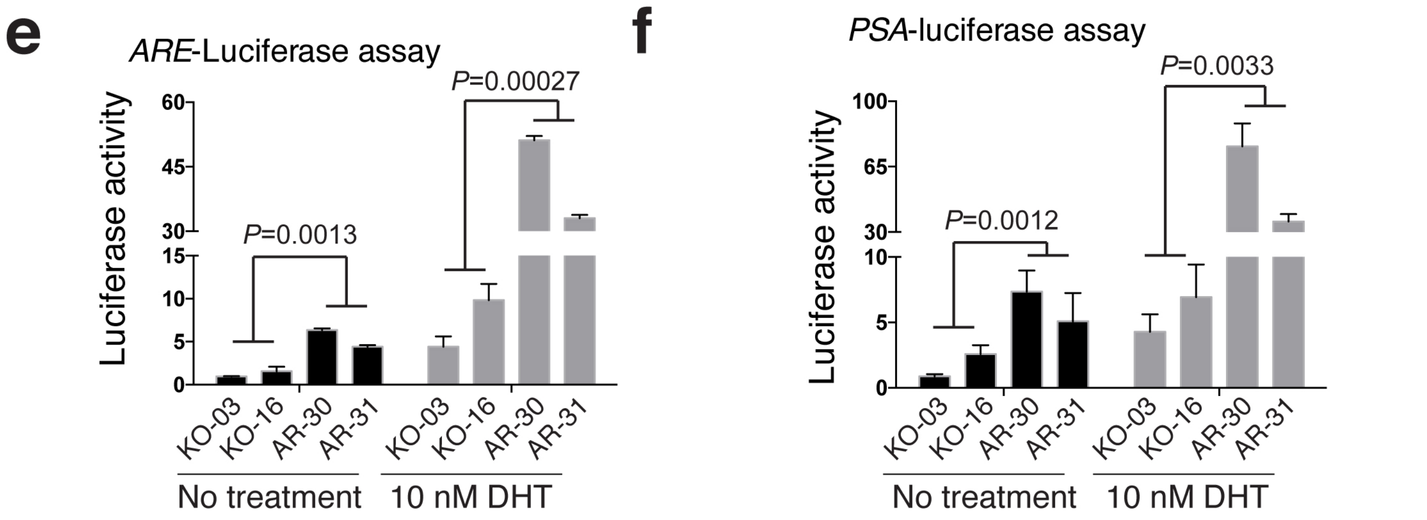

Supplementary Figure 6. The AR⁺ and AR-KO LNCaP clones manifest distinct AR activities.

(**a, b**) IF staining of AR (green; using N20 Ab) in bulk LNCaP cells, and AR-30 and AR-31 clones (a), and in the 3 AR-KO (KO-01, KO-03 and KO-16) clones (b). Original magnifications, x200.

(**c**) WB of AR, TMPRSS2, NKX3.1 and PSA in AR-30, AR-31, KO-03 and KO-16 clones. Relative expression levels were determined using densitometric analysis normalized to respective β-actin, and compared to the individual protein levels in AR-30. Lack of PSA expression and reduced expression of NKX3.1 and TMPRSS2 indicate reduced AR activity in AR-KO cells.

(**d**) qPCR analysis of *AR* and *PSA* mRNA levels in AR-30, AR-31, KO-03 and KO-16 clonal cells. Values represent the mean ± s.e.m. of triplicate samples from 3-5 independent experiments.

(**e, f**) Luciferase (Luc) reporter (*ARE*-luciferase or *PSA*-luciferase) assays to measure AR activities in AR-30, AR-31, KO-03 and KO-16 clonal cells with or without 10 nM DHT treatment. Values represent the mean ± s.e.m. of triplicates from 3-5 independent experiments. *P* values were determined using unpaired Student's *t*-test.

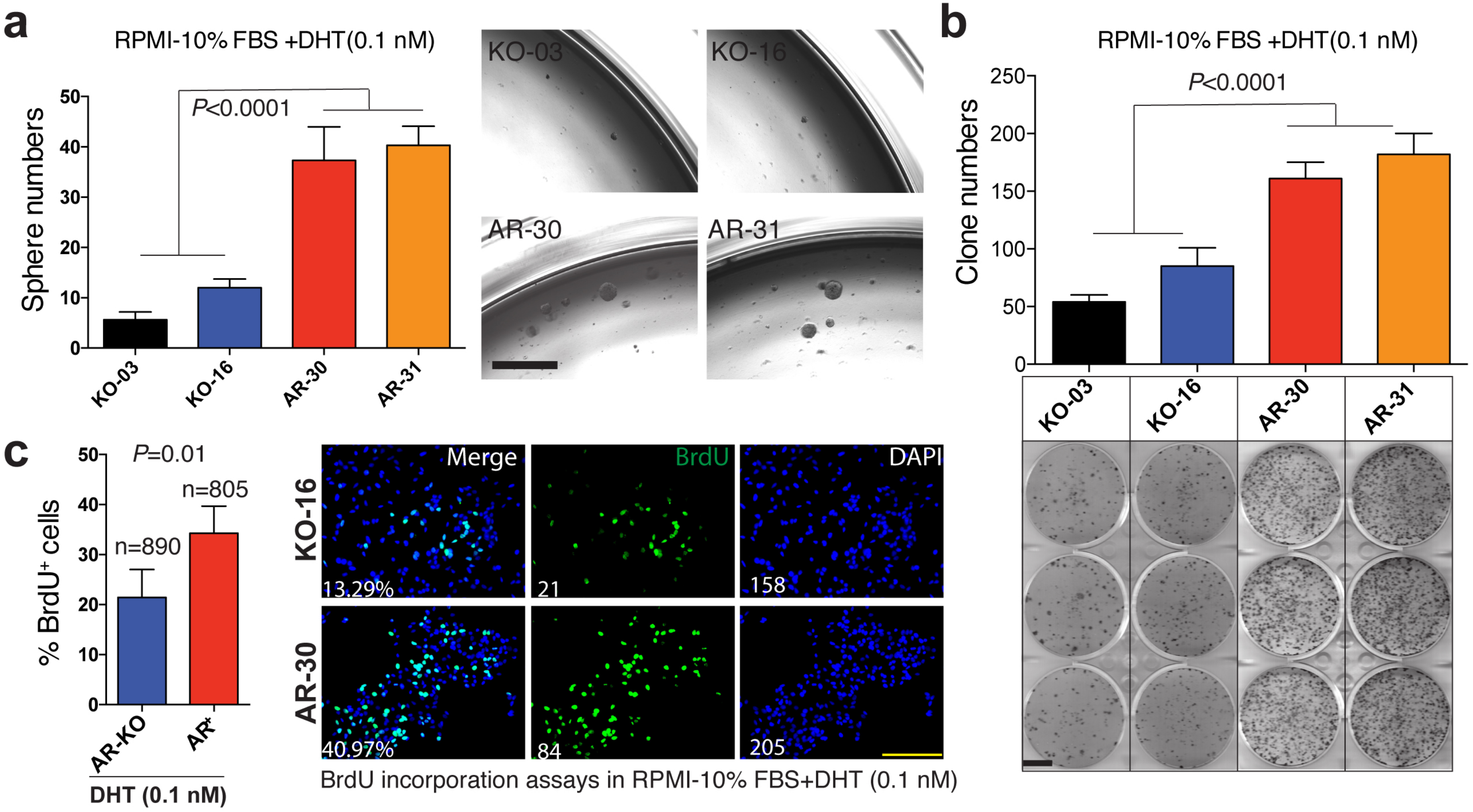

Supplementary Figure 7. AR⁺ LNCaP cells possess higher clonal, sphere-formation, and proliferative capacities than AR-KO LNCaP cells in androgen-containing environment.

(**a**) AR⁺ LNCaP cells (AR-30 and AR-31) display higher sphere-forming activities than AR-KO cells (KO-03 and KO-16) in DHT-containing media (n=3; Student *t*-test). Representative images are shown on the right (Scale bar: 1 μm).

(**b**) AR⁺ LNCaP cells demonstrate higher clonal activities than AR-KO cells in DHT-containing media (n=3; Student *t*-test). Representative images are shown below (Scale bar: 1 cm).

(**c**) AR⁺ LNCaP cells (AR-30) are more proliferative than AR-KO (KO-16) cells in DHT-containing media (n=3; Student *t*-test) assessed by BrdU incorporation assays. Bars (left) represent mean ± s.d. of triplicates from three independent experiments (n, total number of cells analyzed; P, unpaired Student's *t*-test). Shown on the right are representative images of BrdU IF staining with cell numbers indicated on the BrdU and DAPI images and the % BrdU⁺ cells indicated on the 'Merge' images. Scale bar: 250 μm.

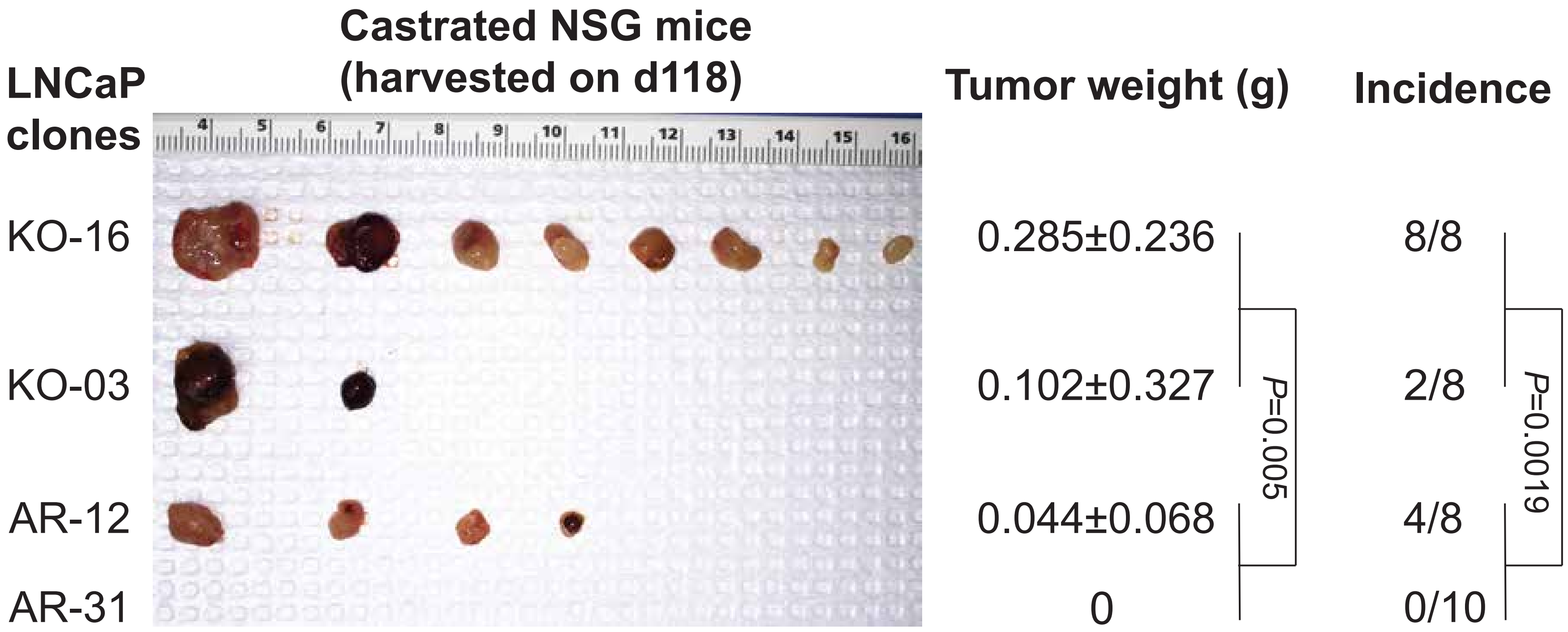

Supplementary Figure 8. AR-KO LNCaP cells are more tumorigenic than AR^{+} cells in castrated hosts.
End-point tumors on day 118 of KO-03, KO-16, AR-12 and AR-31 LNCaP cells implanted in castrated NSG mice. Tumor images, weight and incidence with corresponding *P*-values are indicated. *P*-values for tumor weight and incidence comparisons were determined using unpaired Student's *t*-test and Chi-squared test, respectively.

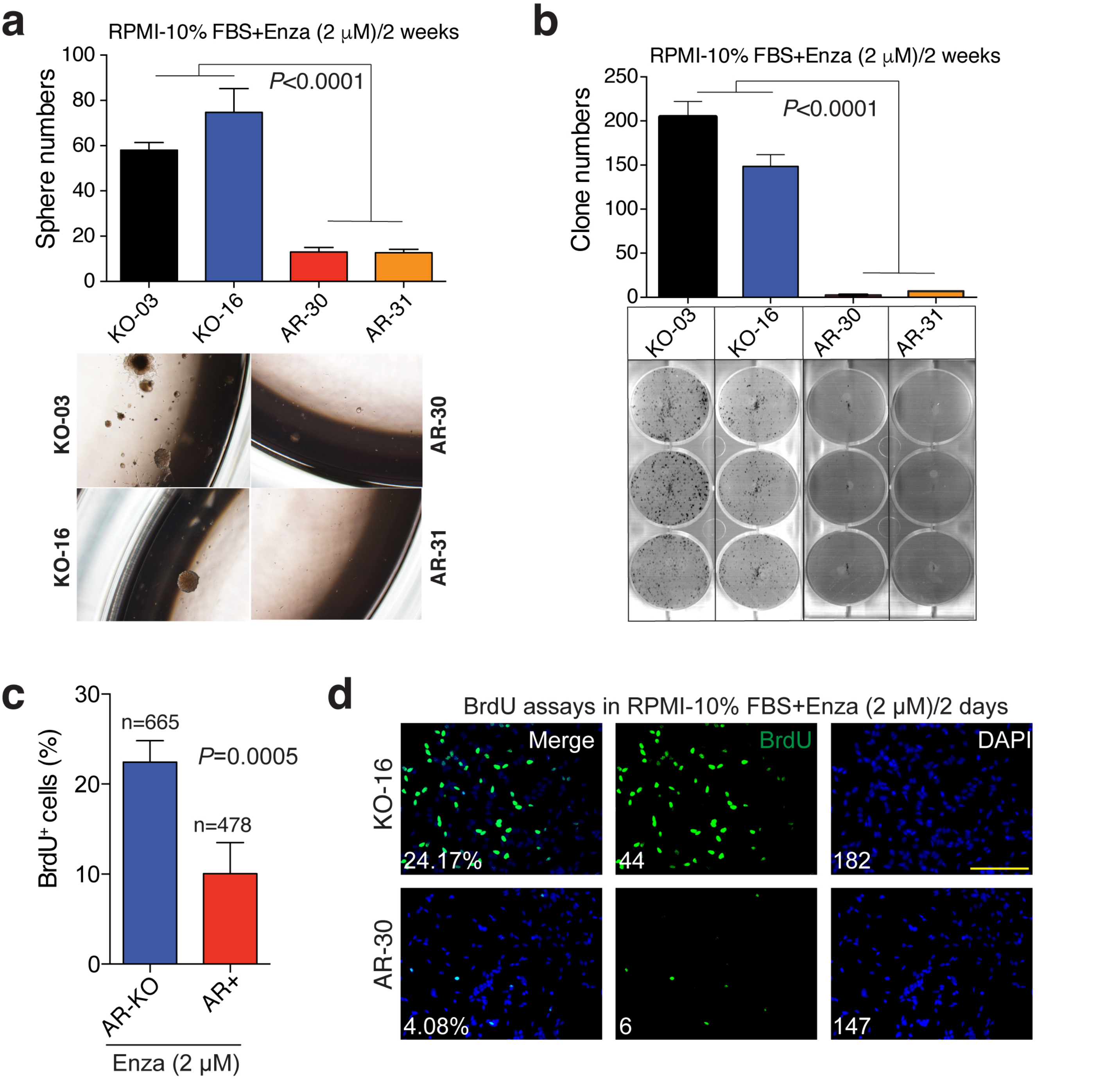

Supplementary Figure 9. AR-KO LNCaP cells resist Enza in vitro and proliferate faster in Enza-containing media.

(**a, b**) AR-KO LNCaP clones possess higher sphere formation (a) and clonal (b) capacities than AR+ clonal cells in response to Enza. Representative images are shown below. Values represent mean ± s.d. of triplicates of three repeat experiments. *P* values were calculated using unpaired Student's *t*-test.

(**c, d**) AR-KO LNCaP cells are more proliferative in the presence of 2 µM Enza. Shown in c is a bar graph presenting % BrdU+ cells in two AR+ (i.e., AR-30 and AR-31) and two AR-KO (KO-03 and KO-16) clones (n=3 experiments; mean ± s.d) with the total numbers of cells counted and *P* value (Student's *t*-test) indicated. Shown in d are representative images of BrdU staining in the indicated clonal AR-KO and AR+ cells in which cell number and % BrdU+ cells are shown on the lower left corners of the images. Scale bar: 250 µm.

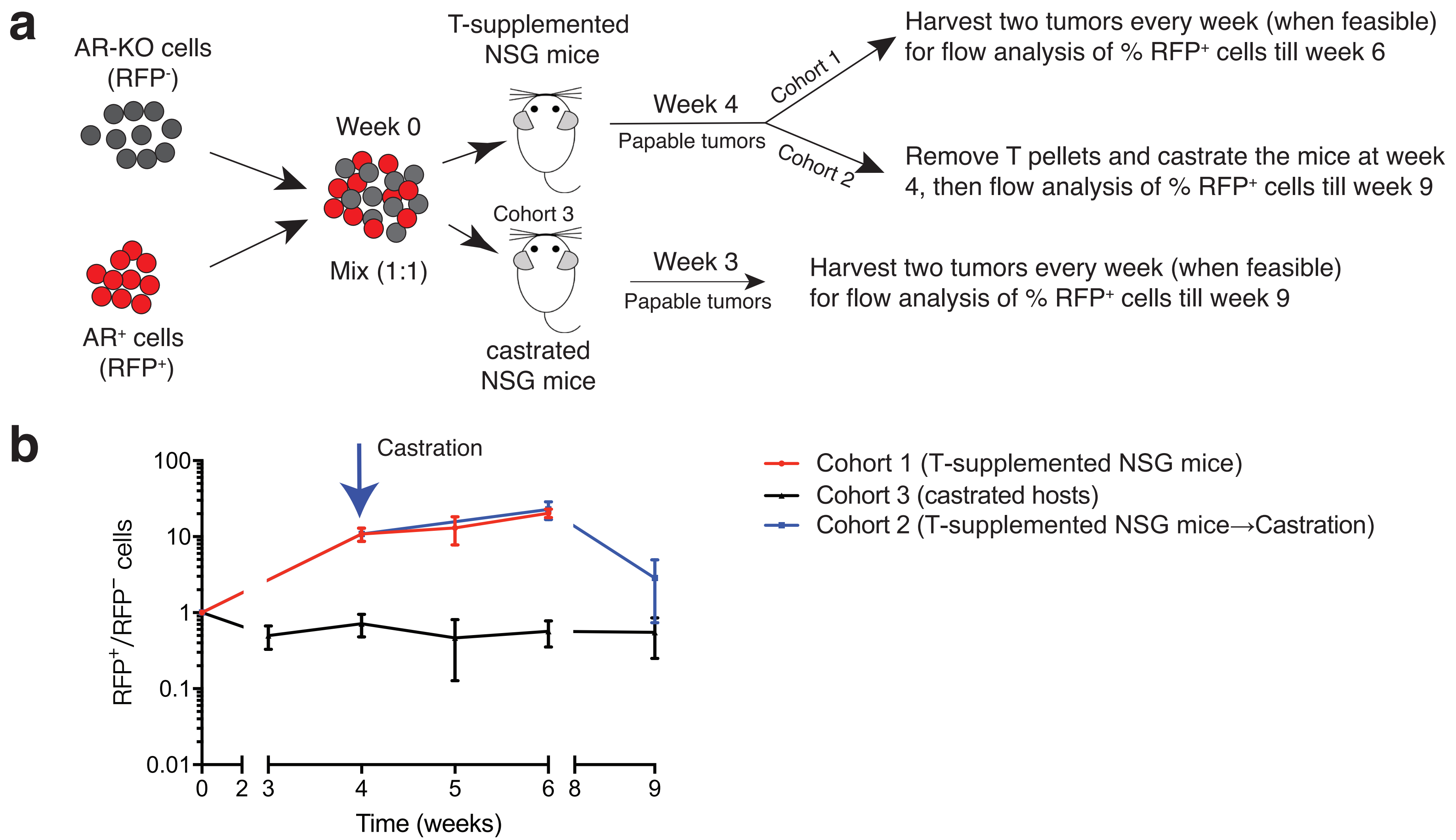

Supplementary Figure 10. Dynamic changes of AR-KO and AR$^+$ LNCaP cells in the tumors in animals with different systemic androgen levels.

(**a**) Schematic of the experimental design for the *in vivo* competition assays. AR-KO (KO-03 or KO-16) and AR-RFP$^+$ (AR-30 or AR-31) cells were mixed (1:1) and implanted (total 400,000 cells/injection) in either T-supplemented male NSG mice (for cohort 1 and 2) or castrated NSG mice (cohort 3).

(**b**) Systemic levels of circulating androgens influence the relative abundance of AR$^+$ vs. AR-KO LNCaP cells in the tumors. Presented are normalized RFP$^+$/RFP$^-$ ratios in the three groups at various time points. Two tumors were analyzed at each condition and time point and data is shown as mean ± SD. The red line represents ratios in tumors from T-supplemented mice (cohort 1), showing that in androgen-proficient mice the AR-RFP$^+$ tumor cells predominated. On the other hand, in castrated mice the AR-KO (RFP$^-$) tumor cells represented the majority in the tumors (cohort 3). When several T-supplemented animals bearing mixed tumors were removed of the T pellets and also castrated at week 4 (cohort 2), although the AR-RFP$^+$ LNCaP cells represented the predominant cell population up till week 6, by week 9, the AR$^+$ cells significantly reduced whereas AR-KO (RFP$^-$) cells dramatically increased.

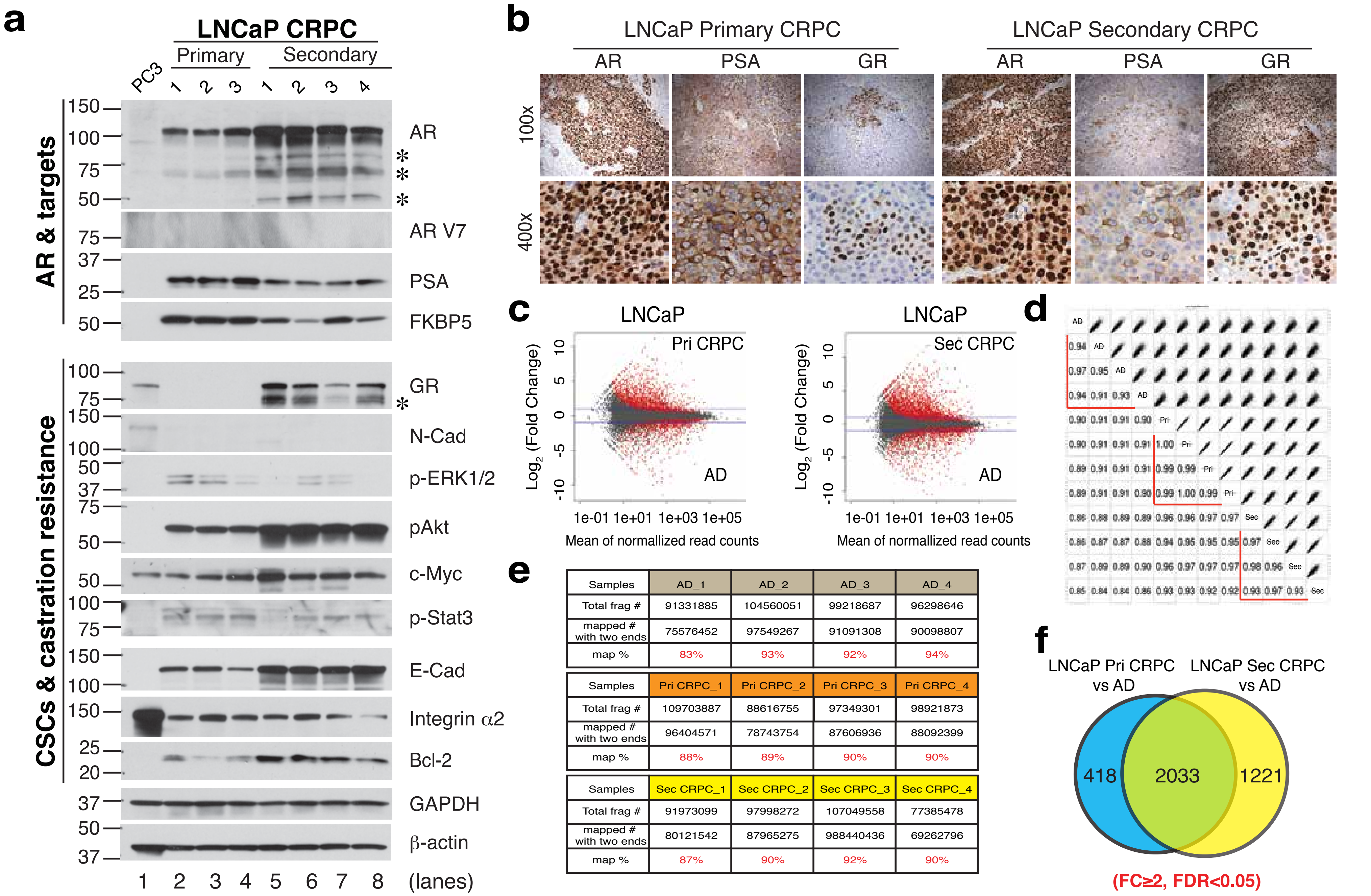

Samples	AD_1	AD_2	AD_3	AD_4
Total frag #	91331885	104560051	99218687	96298646
mapped # with two ends	75576452	97549267	91091308	90098807
map %	83%	93%	92%	94%

Samples	Pri CRPC_1	Pri CRPC_2	Pri CRPC_3	Pri CRPC_4
Total frag #	109703887	88616755	97349301	98921873
mapped # with two ends	96404571	78743754	87606936	88092399
map %	88%	89%	90%	90%

Samples	Sec CRPC_1	Sec CRPC_2	Sec CRPC_3	Sec CRPC_4
Total frag #	91973099	97998272	107049558	77385478
mapped # with two ends	80121542	87965275	988440436	69262796
map %	87%	90%	92%	90%

Supplementary Figure 11. Protein changes in Enza-resistant LNCaP CRPC and RNA-Seq analysis in the LNCaP AD to 1° CRPC to 2° CRPC progression models.

(**a**) Western blotting analysis of the molecules indicated in 3 – 4 representative LNCaP primary (i.e., castration-resistant) and secondary (i.e., both castration- and Enza-resistant) CRPC. PC3 cells were used as controls for molecules tested. Both β-actin and GAPDH were used as loading controls (bottom). *, potential AR and GR splice variants.

(**b**) Representative IHC images of AR, PSA and GR in LNCaP 1° and 2° CRPC endpoint tumors. Original magnifications (100x and 400x) are shown.

(**c**) The MA plot showing the proper normalization of RNA-Seq data in the LNCaP model.

(**d**) The correlation plot showing high consistency in the biological replicates.

(**e**) Mapping rate in LNCaP RNA-Seq and sequencing data were mapped to the reference human genome UCSC version hg38.

(**f**) Venn diagram showing differentially expressed genes (DEGs) between LNCaP 1° CRPC vs. AD and 2° CRPC vs. AD. The statistic threshold was set at fold change (FC) ≥ 2 and false discovery rate (FDR) < 0.05. Of the 2,033 shared genes, 1,437 were upregulated and 596 were downregulated.

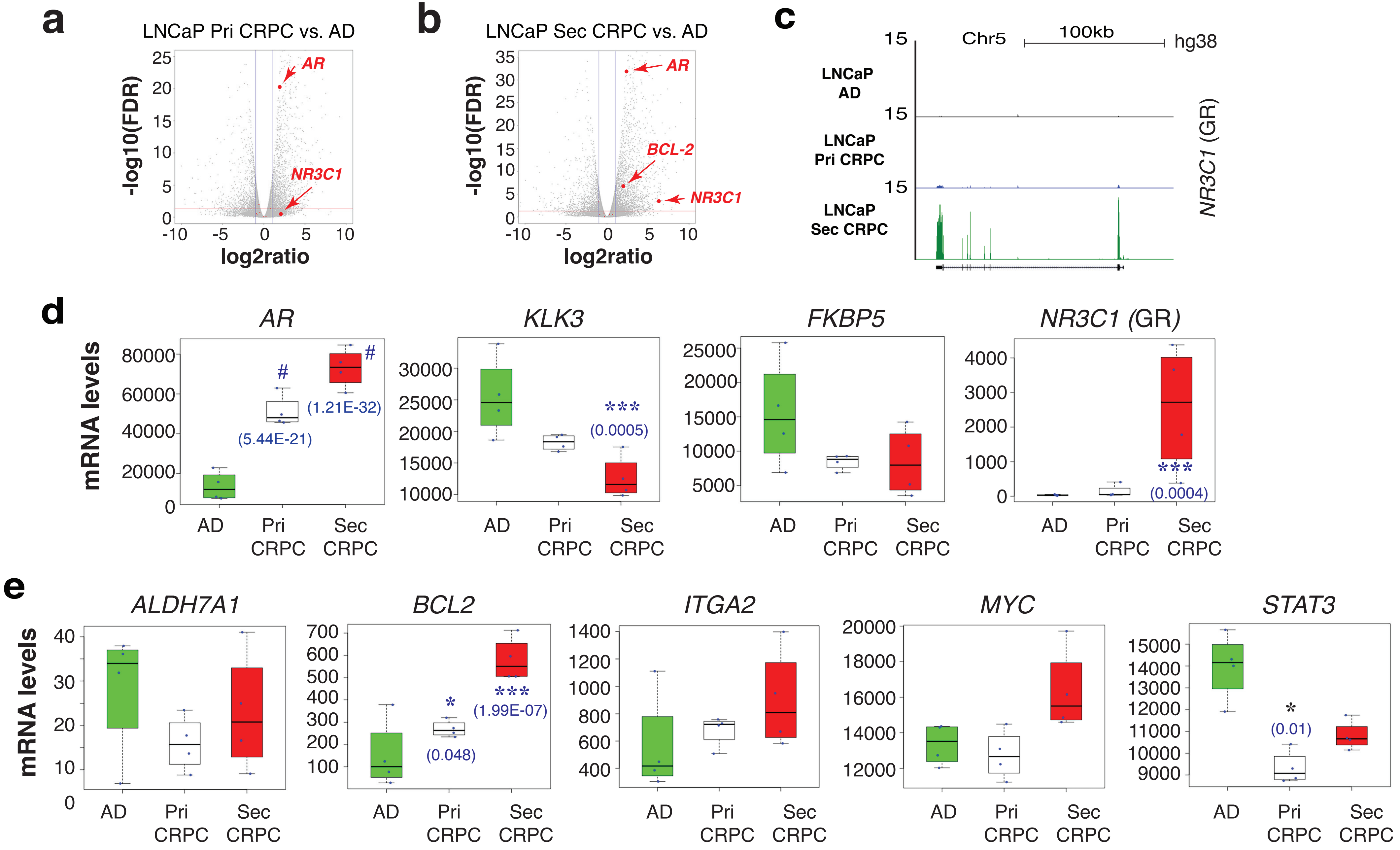

Supplementary Figure 12. Differentially expressed genes in LNCaP CRPC models.

(**a-b**) Volcano plots presenting DEGs (both upregulated and downregulated) in LNCaP 1° CRPC vs. AD (a) and LNCaP 2° CRPC vs. AD (b) tumors. The values (both log2ratio and –log10(FDR)) for each gene represent the mean of the 4 biological replicates. Note overall more upregulated DEGs in the CRPC compared to AD tumors. Several upregulated genes were highlighted by arrows.

(**c**) Significant upregulation of *NR3C1* (GR) mRNA in Enza-resistant LNCaP 2° CRPC. Shown are UCSC tracks from the averaged landscape (n = 4 biological replicates for LNCaP AD tumor, primary CRPC, and secondary CRPC, respectively).

(**d-e**) Box plots showing the relative mRNA levels (DESeq normalized counts) of 4 differentiation related genes (d) and 5 CSC genes (e), whose protein products were analyzed by Western blotting (Fig. 2b; Supplementary Fig. 11a). Statistically significant *P* values are indicated by asterisks (**P*<0.05; ***P*<0.05; ****P*<0.01; #*P*<0.001; compared to AD tumors) and related FDR (q) values indicated below. For the 4 pro-differentiation genes (d), castration induced *AR* mRNA, which was further upregulated by Enza in 2° CRPC. In contrast, the two AR target genes, *KLK3* and *FKBP5*, were decreased in 1°, and, further, in 2° CRPC. Enza (but not castration) selectively upregulated *NR3C1* gene (consistent with c). For 5 CSC genes, only *BCL-2* was induced by castration and further upregulated by Enza. *STAT3* was actually reduced by castration.

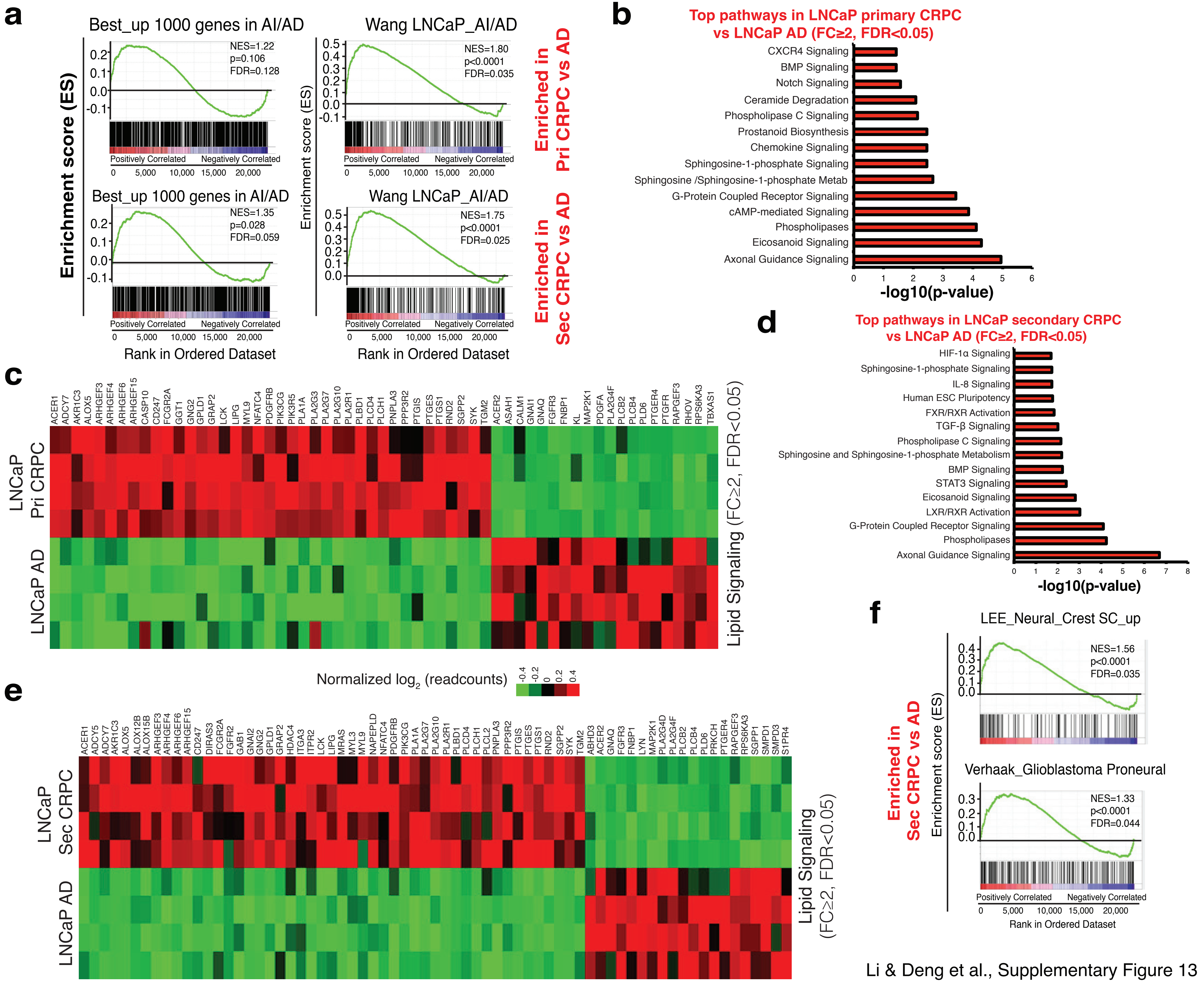

Li & Deng et al., Supplementary Figure 13

Supplementary Figure 13. Novel signaling pathways enriched in LNCaP 1° and 2° CRPC compared to AD tumors.

(a) GSEA showing that both primary (pri) and secondary (sec) LNCaP CRPC (compared to AD) are enriched in gene sets preferentially expressed in ADT-resistant patient CRPC[23] and in castration-resistant (AI) LNCaP cells (see Supplementary Table 3 for GSEA data set).

(b) IPA biological function profiling of 2,451 DEGs (FC≥2, FDR<0.05; Supplementary Data 2) in LNCaP 1° CRPC (n=4) relative to AD tumors (n=4). Shown are selected top pathways.

(c) Heat map of representative lipid signaling molecules altered in LNCaP 1° CRPC vs. AD tumors.

(d) IPA biological function profiling of 3,254 DEGs (FC≥2, FDR<0.05; Supplementary Data 3) in LNCaP 2° CRPC (n=4) relative to AD tumors (n=4). Shown are selected top pathways.

(e) Heat map of representative lipid signaling molecules altered in LNCaP 2° CRPC vs. AD tumors.

(f) GSEA showing that "Neurogenesis" genes preferentially expressed in LNCaP 2° CRPC vs. AD tumors are enriched in the upregulated gene set in neural crest stem cells[51] and in the proneural subtype of glioblastoma[52].

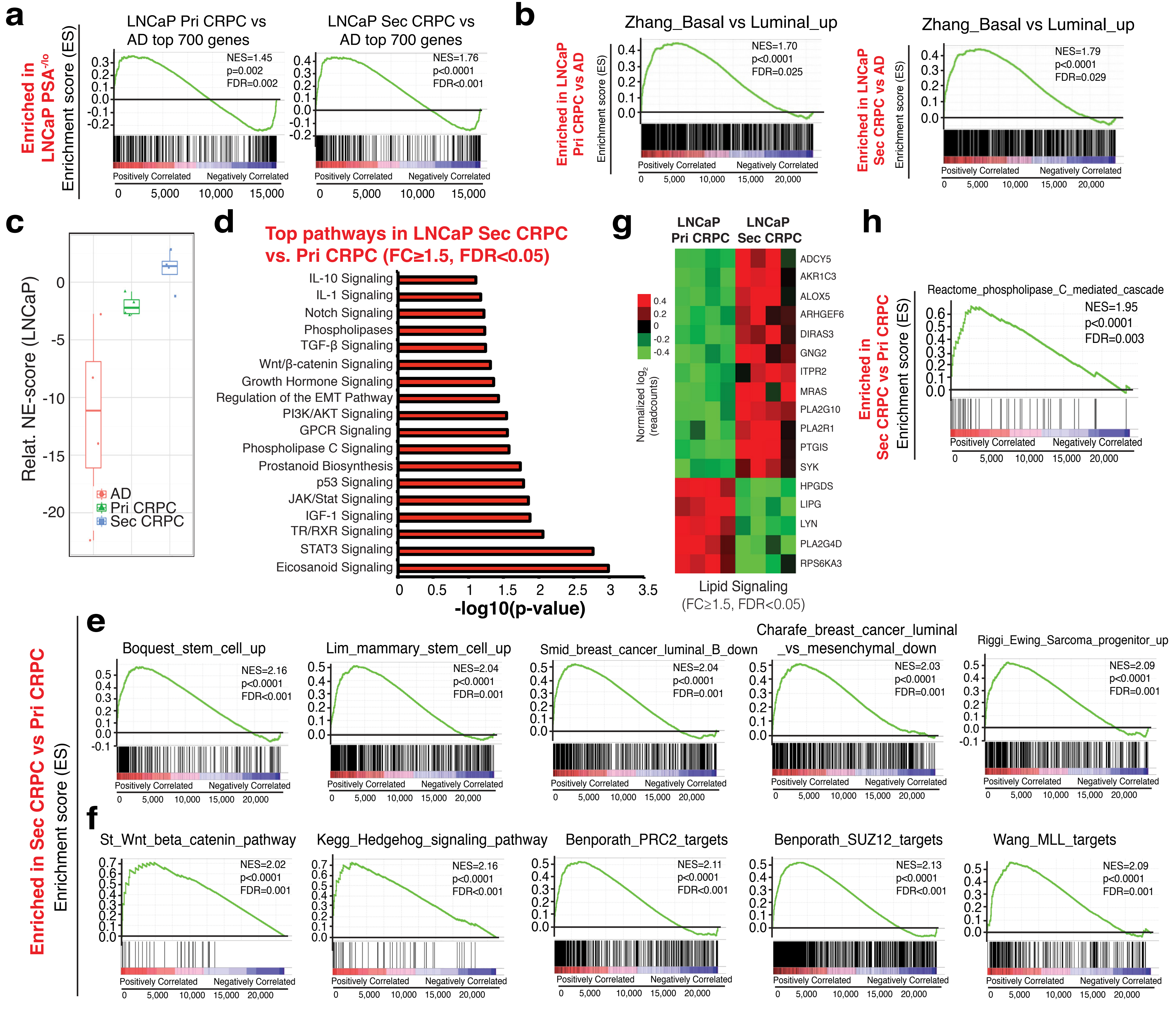

Li & Deng et al., Supplementary Figure 14

Supplementary Figure 14. Association of LNCaP CRPC gene expression profiles with NEPC and novel signaling pathways enriched in LNCaP 2° (i.e., Enza-resistant) CRPC compared to 1° CRPC.

(a) The top 700 DEGs in both LNCaP 1° CRPC (vs. AD) and LNCaP 2° CRPC (vs. AD) are highly enriched in $PSA^{-/lo}$ LNCaP cells.

(b) Both LNCaP 1° CRPC (vs. AD) and LNCaP 2° CRPC (vs. AD) DEGs positively correlate with the neurogenesis gene expression profiles in normal human prostatic basal/stem cells.

(c) Increasing NE_scores in LNCaP 1° and 2° CRPC compared to AD tumors.

(d) IPA biological function profiling of 601 DEGs (FC≥2, FDR<0.05; Supplementary Data 4) in LNCaP 2° CRPC (n=4) relative to 2° CRPC (n=4). Shown are selected top pathways.

(e) GSEA showing enrichment of multiple stem cell gene signatures in LNCaP 2° CRPC vs. 1° CRPC.

(f) GSEA showing enrichment of stem cell and epigenetic signaling pathways or their target genes in LNCaP 2° CRPC vs. 1° CRPC.

(g) Heat map showing representative lipid signaling molecules altered in LNCaP 2° vs. 1° CRPC.

(h) GSEA showing association of a phospholipase C gene signature with LNCaP 2° CRPC.

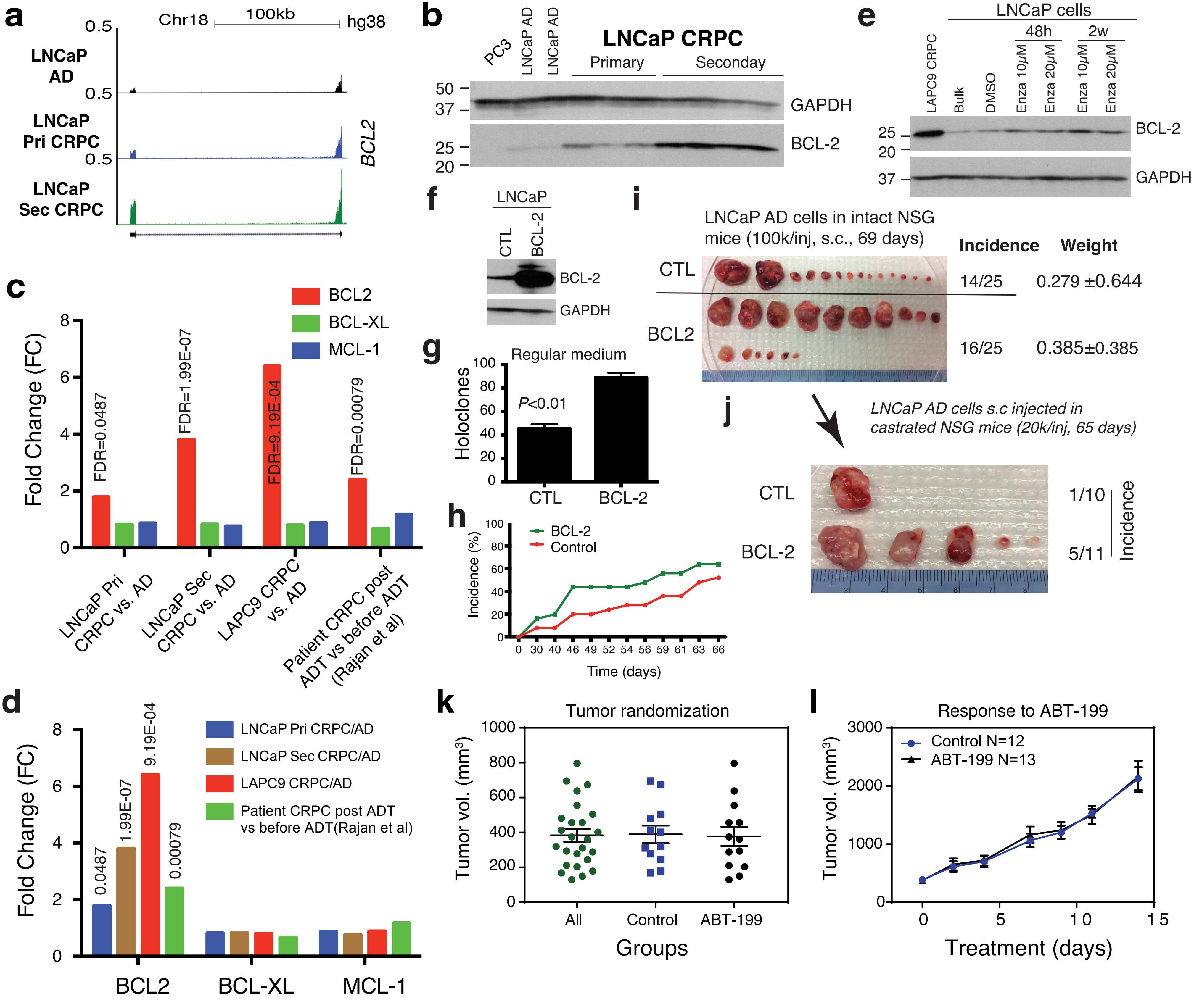

Li & Deng et al., Supplementary Figure 15

Supplementary Figure 15. Enza directly upregulates BCL-2 to confer androgen-independent tumor growth.

(a) RNA-Seq tracks showing upregulation of *BCL-2* mRNA in LNCaP 1° and 2° CRPC. Shown are UCSC tracks from averaged landscape (n=4 biological replicates for LNCaP AD tumor, primary CRPC, and secondary CRPC, respectively).

(b) WB showing upregulation of BCL-2 protein in LNCaP 1° CRPC and, further, in 2° CRPC.

(**c-d**) BCL-2, but not BCL-XL or MCL-1, was upregulated in not only LNCaP and LAPC9 CRPC but also patient CRPC. Shown are two different ways to present exclusive upregulation of *BCL-2*.

(e) Enza induces BCL-2 expression. WB analysis of BCL-2 in LNCaP cells treated with Enza (2 concentrations) for either 48 h or 2 weeks (w). LAPC9 CRPC lysate was used as control.

(f) BCL-2 overexpression in LNCaP cells assessed by WB.

(g) BCL-2 overexpression promotes LNCaP cell clonal (i.e., holoclone) growth in regular media.

(h) BCL-2 overexpression promotes rapid LNCaP AD tumor growth in intact male NSG mice manifested as increased palpable tumor incidence (i.e., earlier tumor appearance).

(i) BCL-2 overexpression promotes LNCaP AD tumor growth in intact male NSG mice manifested as more endpoint large tumors when animals were terminated on day 69 (Note that the *P* value for tumor weight comparison in the two groups was at the borderline due to large variations in tumor sizes).

(j) BCL-2 overexpression promotes AI tumor regeneration. BCL-2 overexpressing LNCaP AD tumors regenerated more tumors when transplanted into castrated NSG mice.

(**k-l**) ABT-199 does not inhibit the growth of LNCaP primary CRPC. Castrated male NSG mice bearing subcutaneous LNCaP 1° CRPC were randomized into two groups when tumors reached ~400 mm^3 (k) and then treated with ABT-199 (100 mg/kg, oral gavage, Mon-Fri) or vehicle control (5% DMSO, 50% PEG30, and 5% Tween 80) and tumor volumes were measured (l).

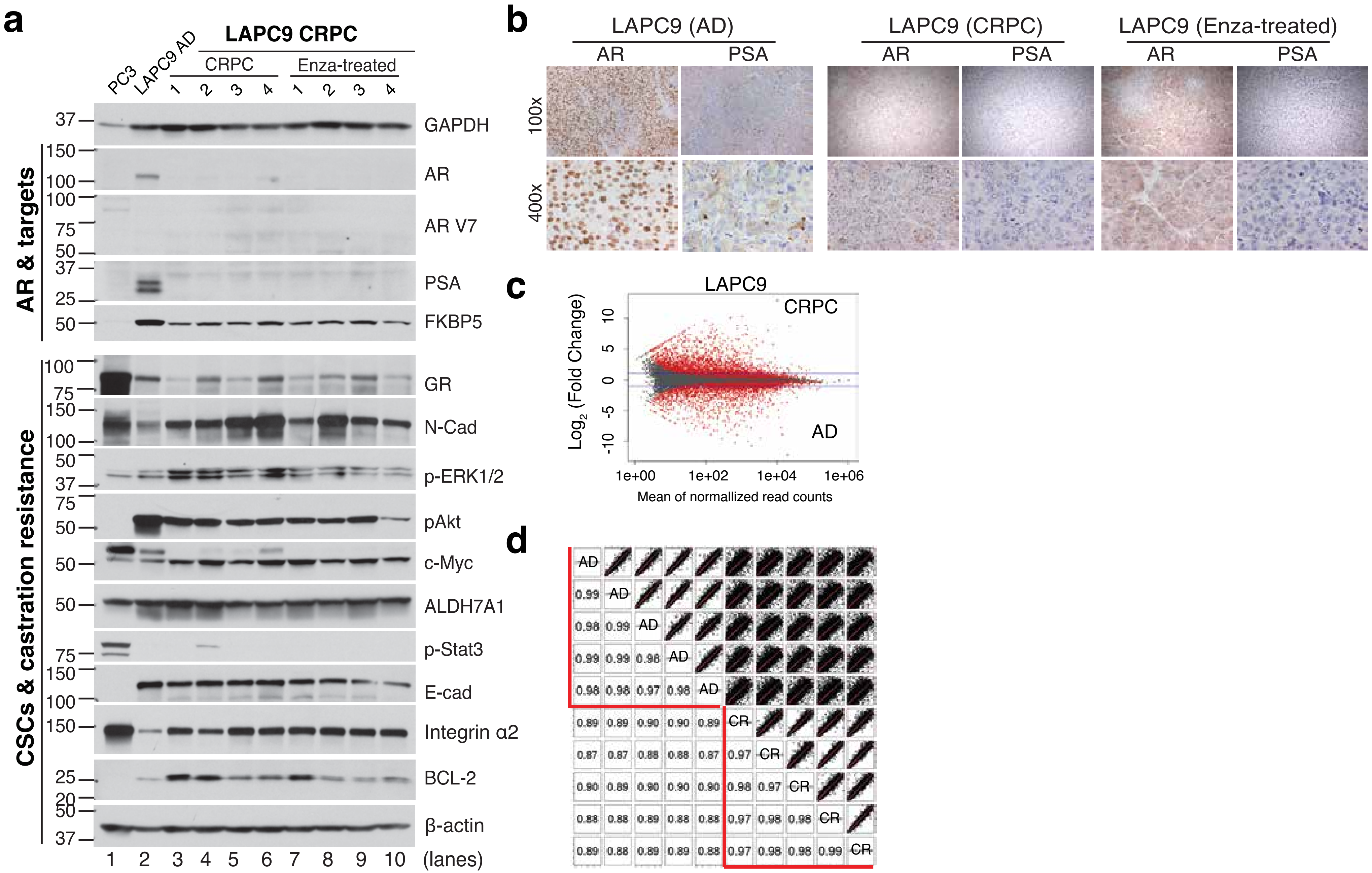

e

Samples	LAPC9 AD_1	LAPC9 AD_2	LAPC9 AD_3	LAPC9 AD_4	LAPC9 AD_5	LAPC9 CR_1	LAPC9 CR_2	LAPC9 CR_3	LAPC9 CR_4	LAPC9 CR_5
Total frag #	77124335	72889996	76289856	59405395	82657415	74480005	79283170	75935469	74810037	69407355
mapped # with two ends	62670649	60800909	65208224	47579559	68955077	63108728	70113572	630313489	66721696	62230909
map %	81%	83%	85%	80%	83%	85%	88%	83%	89%	90%

Li & Deng et al., Supplementary Figure 16

Supplementary Figure 16. Biochemical changes in Enza-treated LAPC9 CRPC and RNA-Seq analysis in LAPC9 AD and CRPC models.

(a) Western blotting analysis of the molecules indicated in 4 representative CR and 4 Enza-treated LAPC9 tumors (Fig. 1f). One LAPC9 AD tumor and PC3 cells were used as controls for molecules tested. Both β-actin (bottom) and GAPDH (top) were used as loading controls.

(b) Representative IHC images of AR and PSA in AD, CR, and Enza-treated LAPC9 tumors. Original magnifications are shown.

(c) The MA plot showing proper normalization of RNA-Seq data in the LAPC9 AD and CR tumors.

(d) The correlation plot showing high consistency in the biological replicates.

(e) Mapping rate (hg38) in LAPC9 RNA-Seq was 80-90%, indicating high quality of the RNA sequencing data.

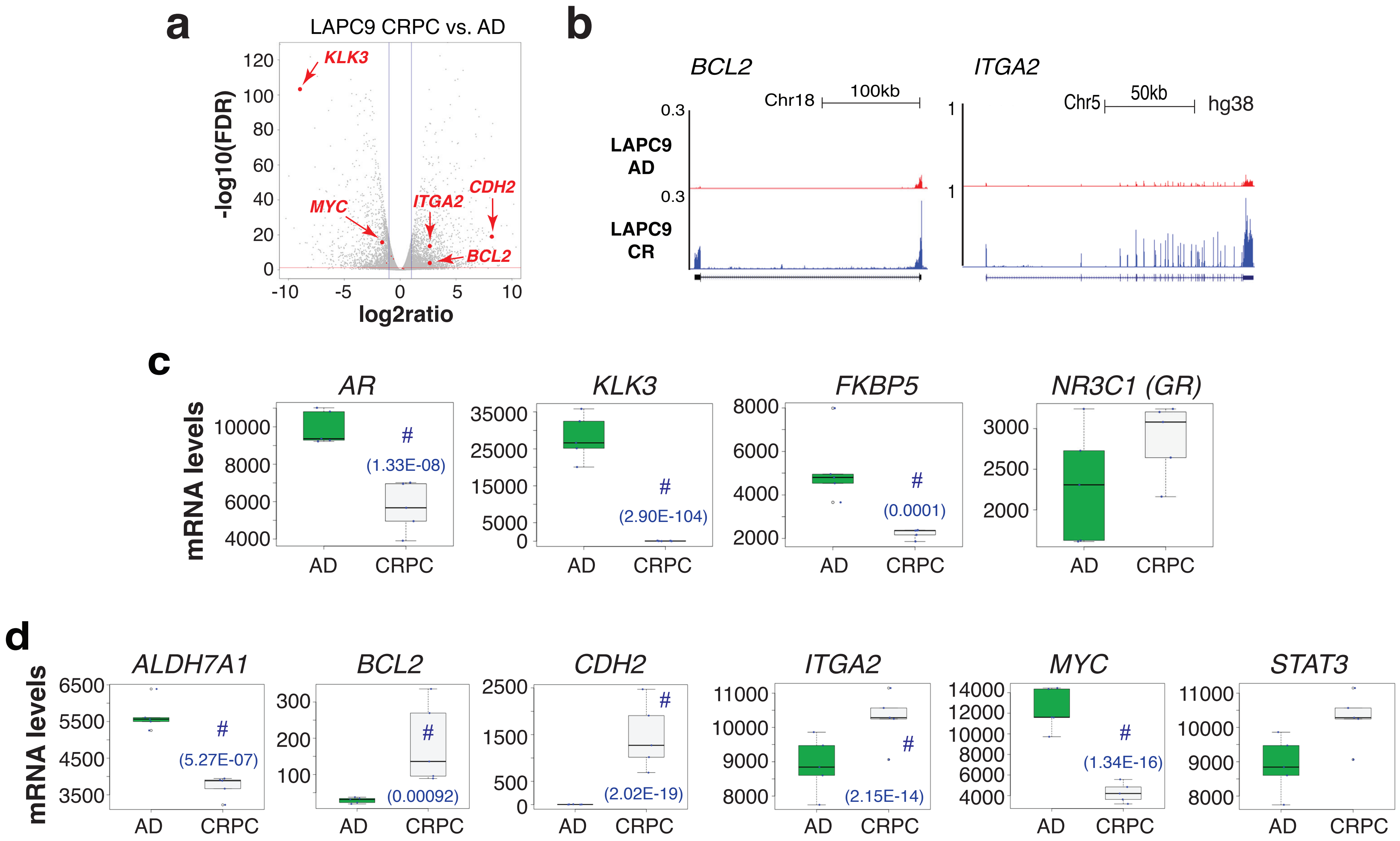

Supplementary Figure 17. Differentially expressed genes (DEGs) in the LAPC9 AD and CRPC models.

(**a**) Volcano plots presenting DEGs (both upregulated and downregulated) in LAPC9 CRPC vs. AD tumors (n=5 each). The values (both log2ratio and –log10(FDR)) for each gene represent the mean of 5 biological replicates. Several DEGs were highlighted by arrows.

(**b**) Upregulation of *BCL-2* and *ITGA2* mRNAs in LAPC9 CRPC. Shown are UCSC tracks from the averaged landscape (n=5 biological replicates for LAPC9 AD and castration-resistant(CR) tumors, respectively).

(**c-d**) Box plots showing the relative mRNA levels (DESeq normalized counts) of 4 differentiation related genes (c) and 6 CSC genes (c) whose protein products were analyzed by Western blotting (Fig. 2a; Supplementary Fig. 16a). Statistically significant *P* values (#*P*<0.001; compared to AD tumors) and related FDR (q) values are indicated. For the 4 pro-differentiation genes (c), castration significantly decreased mRNA levels of both *AR* and the two AR target genes, *KLK3* and *FKBP5*. The *NR3C1* mRNA levels were slightly increased in $AR^{-/lo}$ LAPC9-CRPC although this increase was not statistically significant. Amonth the 6 CSC genes examined, 3 (*BCL-2, CDH2, ITGA2*) were significantly upregulated and *STAT3* showed statistically insignificant increase whereas *ALDH7A1* and *MYC* mRNA levels were significantly decreased in LAPC9-CRPC.

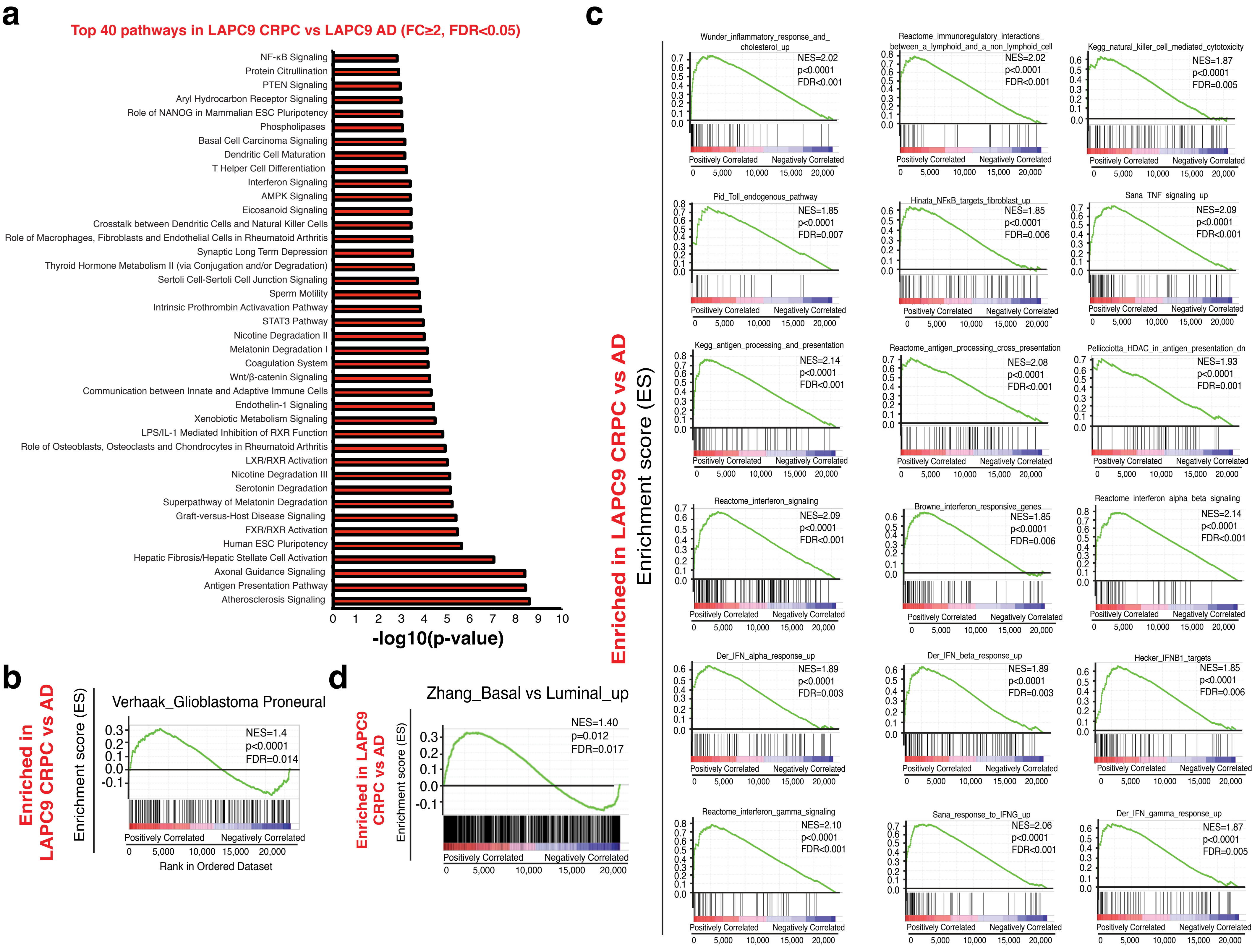

Li & Deng et al., Supplementary Figure 18

Supplementary Figure 18. Potential novel signaling pathways deregulated in LAPC9 CRPC.

(a) IPA of the 3,929 DEGs in LAPC9 CRPC (vs. AD tumors; Supplementary Data 5) revealed enrichment of many pathways, especially those involved in Stem Cell, Lipid, Neuorogenesis, and Immune/Inflammatory signaling. Shown are the top 40 unselected pathways.

(b) GSEA showing enrichment of gene signatures related to Immune/Inflammatory responses, especially the interferon signaling, in LAPC9 CRPC.

(c) GSEA showing that LAPC9 CRPC (vs. AD) DEGs positively correlated with the neurogenesis gene-enriched profiles in normal human prostate basal/stem cells[25].

(d) GSEA showing that genes preferentially expressed in the LAPC9 CRPC correlated with gene expression signature in Proneural subtype of gliobalstoma[42].

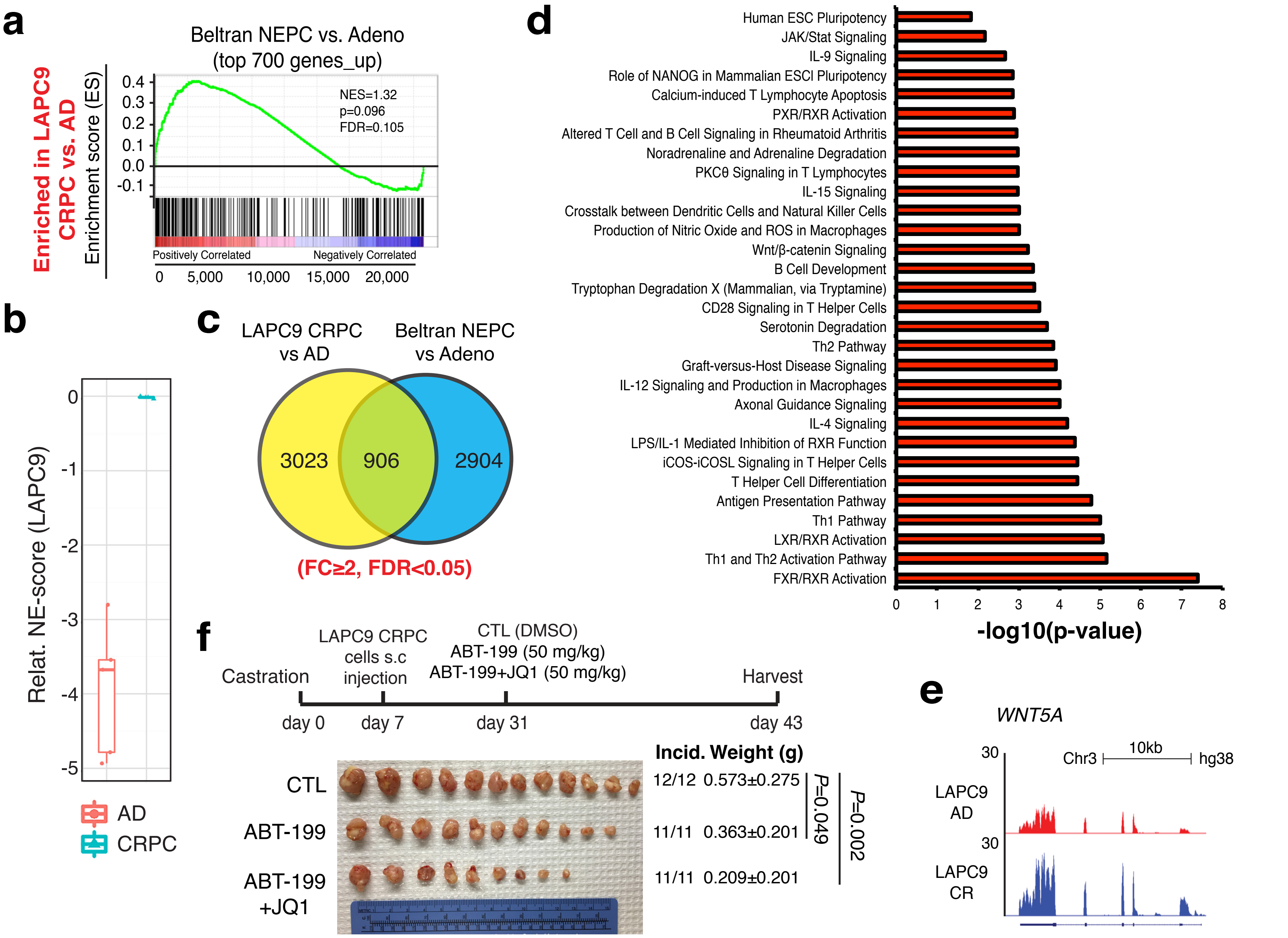

Li & Deng et al., Supplementary Figure 19

Supplementary Figure 19. Association of AR$^{-/lo}$ LAPC9 CRPC with CRPC-NE phenotype and novel therapeutic strategies targeting cancer stem cell molecules.

(**a**) GSEA showing that LAPC9 CRPC gene expression profile correlates with CRPC-NE signature[24]. GSEA was conducted using the top 700 genes *upregulated* in CRPC-NE over CRPC-Adeno in the Beltran dataset[24]. Note that in GSEA FDR carries more weight than p values and an FDR of ≤0.25 is considered significantly different. A similar GSEA using the top 400 genes *downregulated* in CRPC-NE over CRPC-Adeno in the Beltran dataset[24] revealed NES = -1.31, p=0.046, and FDR=0.054 (data not shown), against supporting that the LAPC9 CRPC gene expression profile correlates with CRPC-NE signature.

(**b**) Increased NE_score in LAPC9 CRPC compared to AD tumors.

(**c**) Venn diagram showing the 906 (528 upregulated and 378 donwregulated) genes shared in LAPC9 CRPC/AD DEGs and the Beltran NEPC (CRPC-NE)/Adeno (CRPC-Adeno) DEGs[24] (see Supplementary Data 6 for the gene list).

(**d**) IPA of the 906 genes reveals the top 30 pathways in LAPC9 CRPC associated with Stem Cell, Lipid, Neurogenesis, and Immune/Inflammation signaling.

(**e**) UCSC genome browser view of RNA-Seq signals in the *WNT5A* gene region. Shown are UCSC tracks from the averaged landscape (n = 5 biological replicates for AD and CR (castration-resistant) tumors, respectively).

(**f**) Novel strategies to treat AR$^{-/lo}$ LAPC9 CRPC. Shown on top is the time line for LAPC9-CRPC treatment. NOD/SCID male mice were castrated on day 0, LAPC9-CRPC cells were injected s.c on day 7, and 3 treatment regimens indicated were initiated on day 31 when tumors reached ~50 mm^3. Shown below are endpoint tumors (harvested on day 43), tumor incidence and weight, and statistics for weight (Student's *t*-test).

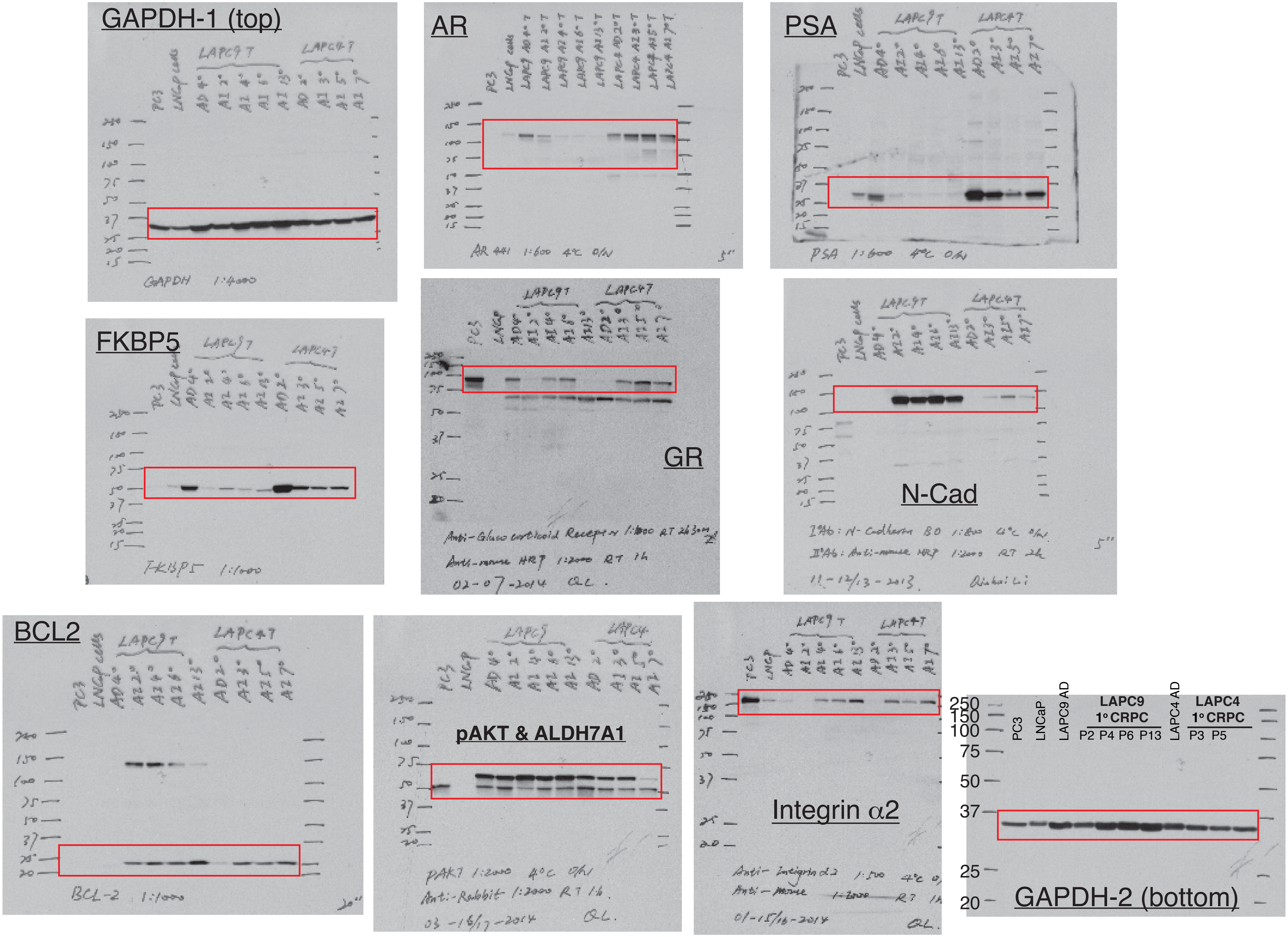

Li & Deng et al., Supplementary Fig. 20 (representative original films for Fig. 2a)

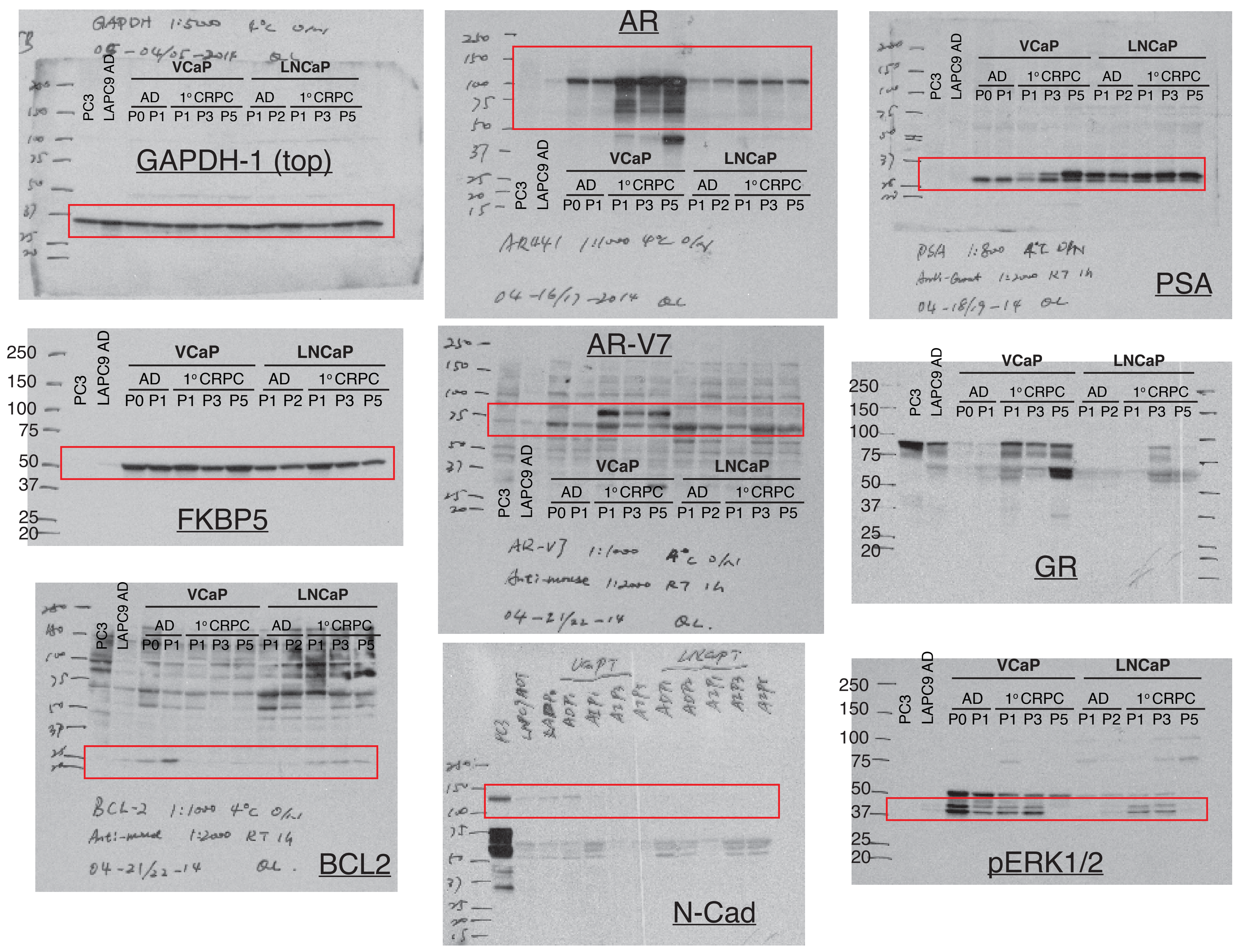

Li & Deng et al., Supplementary Fig. 21 (representative original films for Fig. 2b)

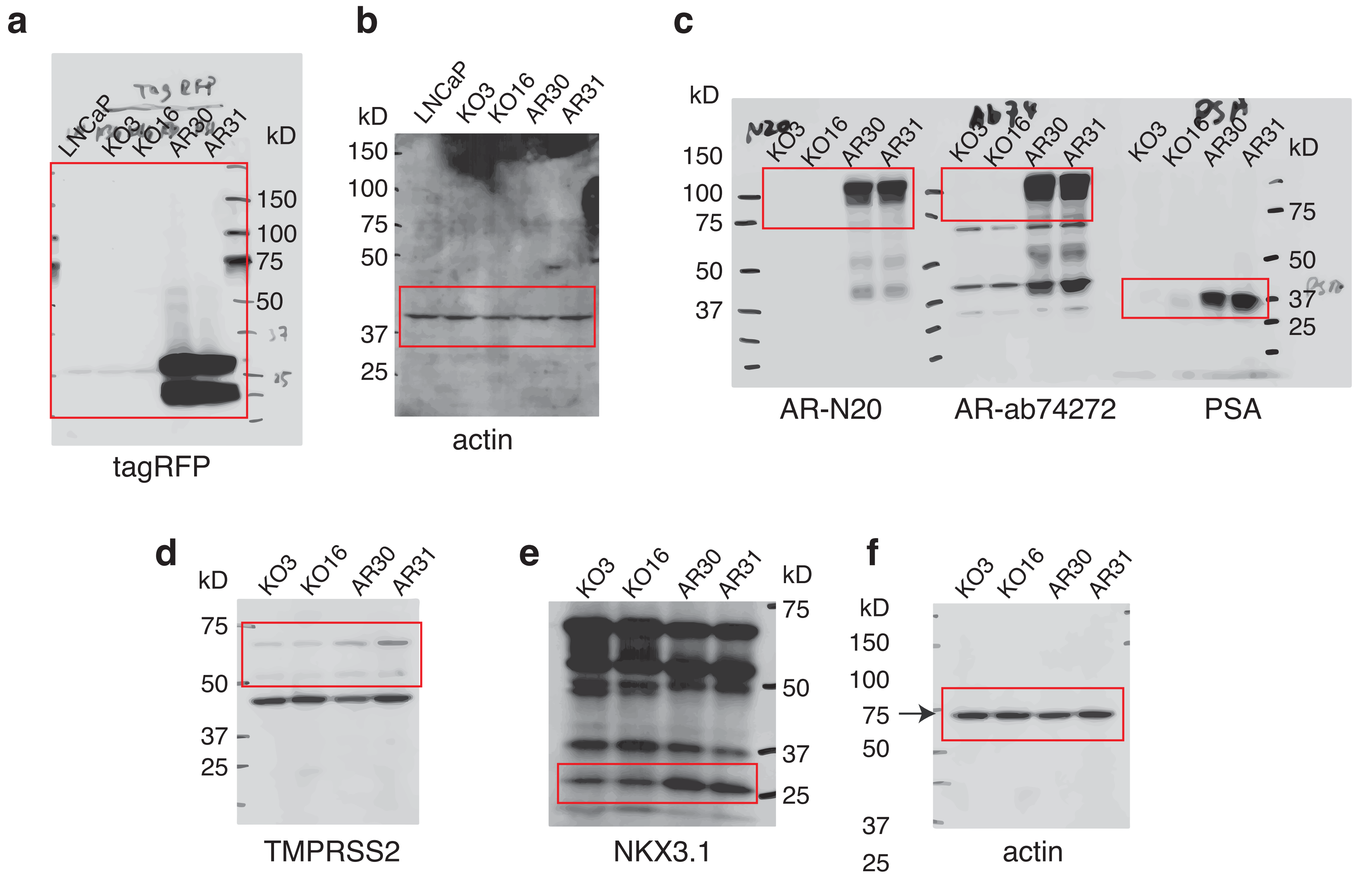

Supplementary Figure 22. Representative full-film images characterizing AR-RFP and AR-KO LNCaP cell clones.

(**a-b**) RPF reports endogenous AR in LNCaP cells (a). Shown in b is the actin loading control. For both tagRFP and actin, the bands in boxed rectangles were presented in Supplementary Fig. 4b.

(**c-f**) Lack of full-length AR and PSA, and, reduced expression of AR target proteins, TMPRSS2 and NKX3.1, in AR KO LNCaP clones. Shown are full-film images for the results in Supplementary Fig. 6c. Shown in c, the left and middle panels, are two AR blotting images detected using the two indicated N-ter anti-AR antibodies. PSA was detected as a ~32-34 kD doublet as widely reported. Shown in d is TMPRSS2 detected as a ~54 kD band and a ~60 kD band (likely glycosylated form). Shown in d is the detection of NKX3.1 as a 28 kD protein (boxed). All antibodies used are listed in Supplementary Table 2.

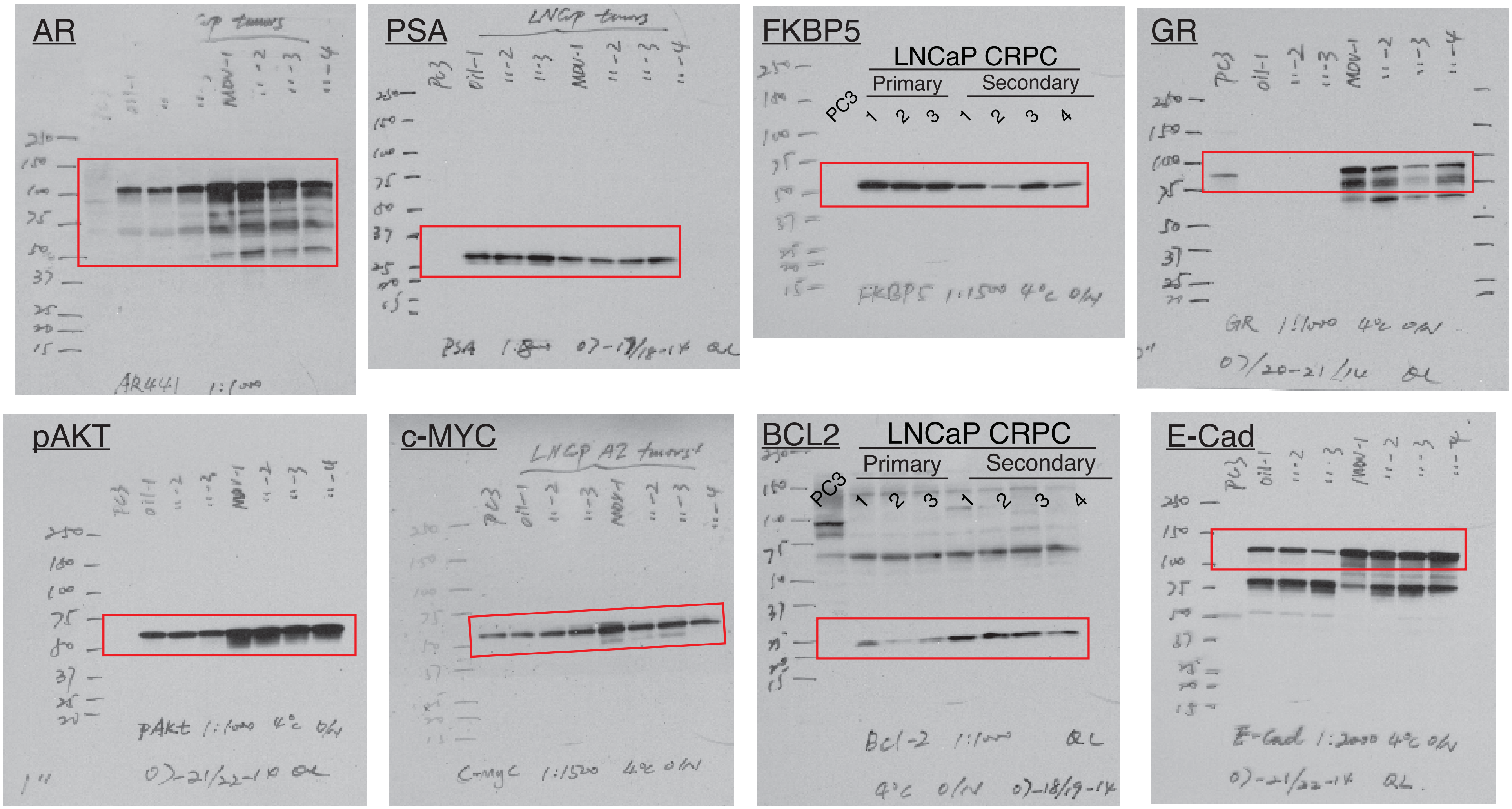

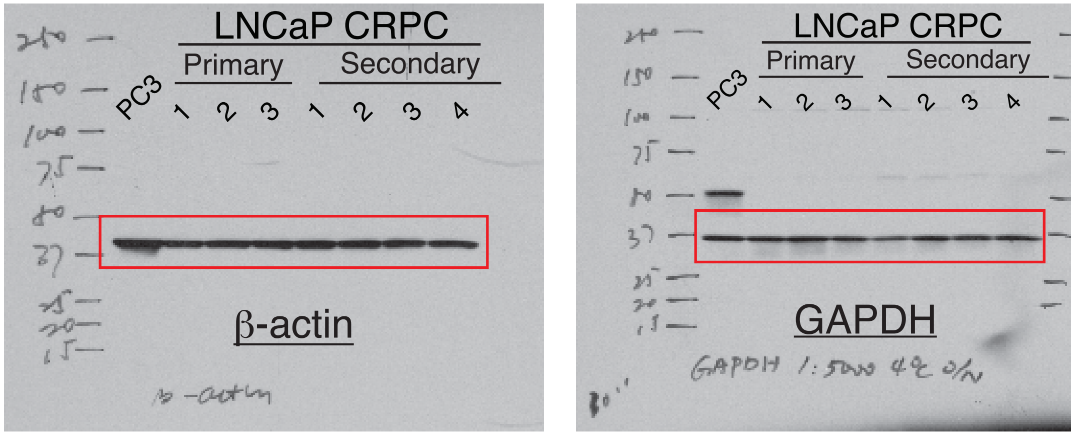

Li & Deng et al., Supplementary Fig. 23
(representative full-film images
for Supplementary Fig. 11a)

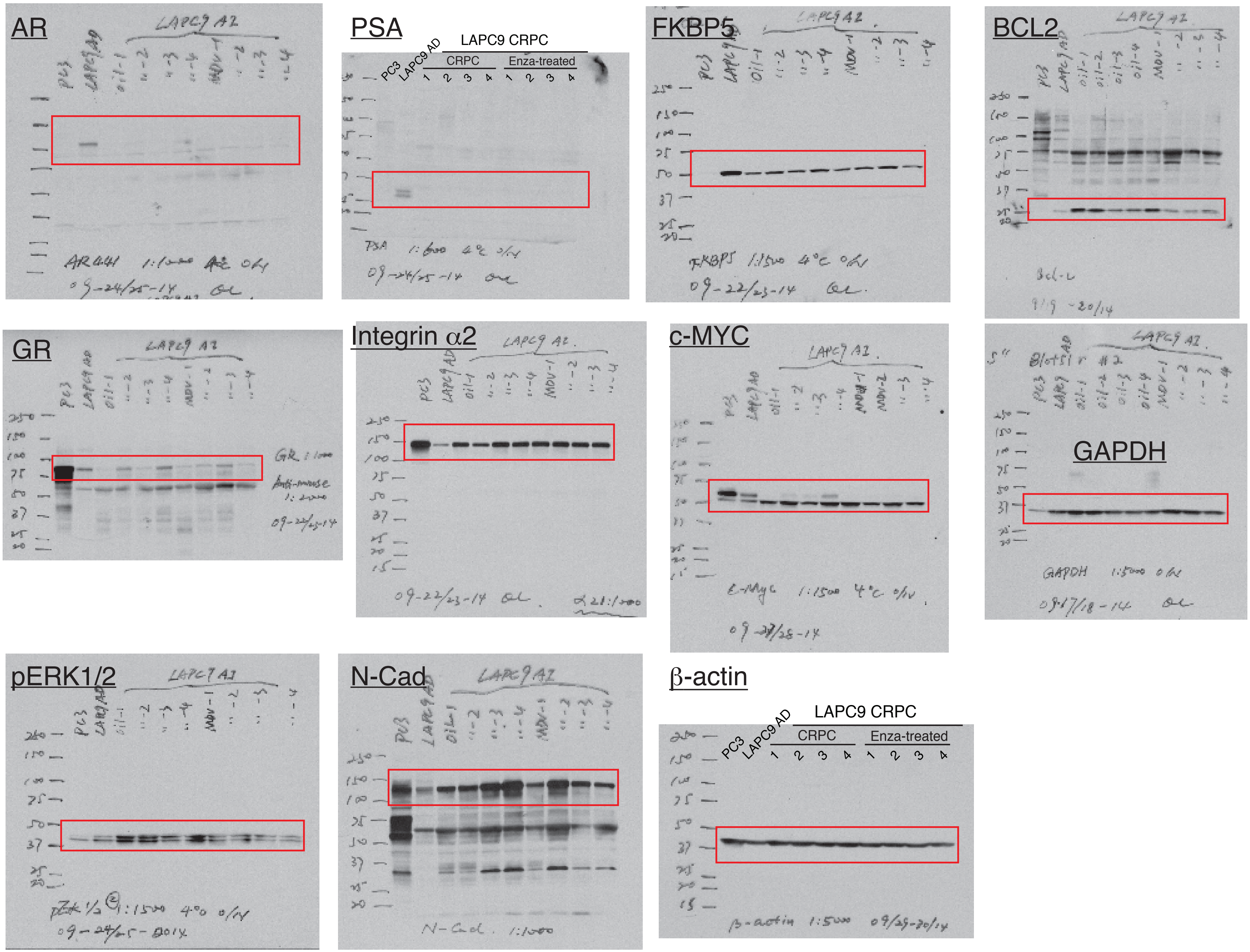

Li & Deng et al., Supplementary Fig. 24 (representative full-film images for Supplementary Fig. 16a)

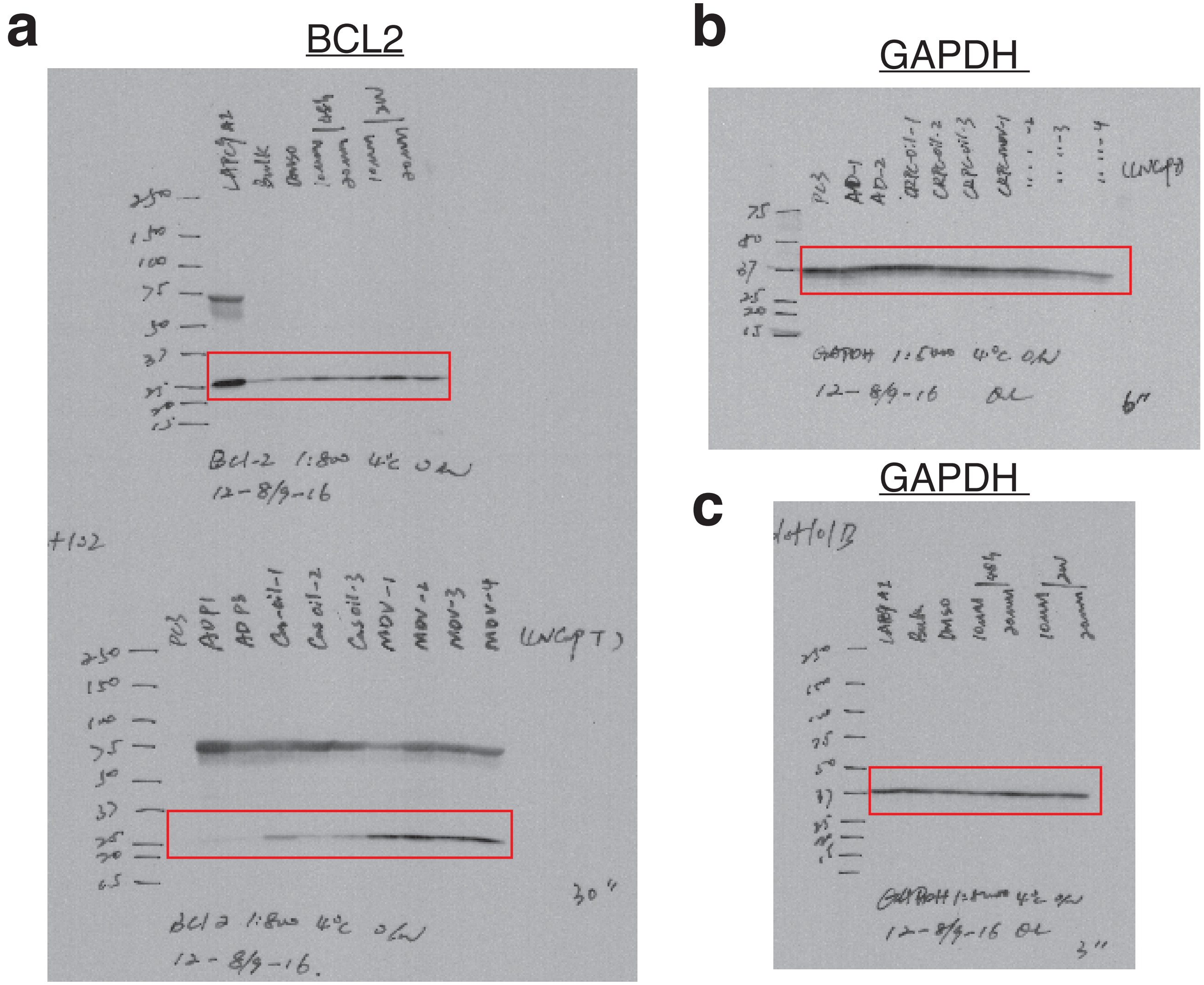

Supplementary Figure 25. Representative full-film images showing castration/Enza induced BCL-2 in the LNCaP model. BCL-2 expression was induced in LNCaP cells by Enza treatment for 48 h or 2 weeks (**a**; the upper panel). The boxed BCL-2 bands were presented in Supplementary Fig. 15e (shown in panel **c** is the corresponding GAPDH). Shown in the lower panel of **a** is the BCL2 induction in primary LNCaP CRPC (i.e., animals bearing castrated tumors treated with the vehicle control, oil) and further induction of BCL-2 in secondary CRPC treated with Enza (MDV3100). The boxed region was presented in Supplementary Fig. 15b (and the corresponding GAPDH was presented in panel **b**).

Supplementary Table 1. Primary antibodies used in the current study

Antibody	Company	Catalog#	Host/Type	Usage	Dilution	Remarks
ABCG2	SIGMA	AV43649	Rabbit pAb	WB	1:500	
ALDH	BD Transduction Laboratories	611195	Mouse mAb	WB	1:500	aa 7-128
ALDH7A1	Abcam	ab53278	Rabbit mAb	WB	1:1000	EP1935Y
AKT	Cell Signaling	4691	Rabbit mAb	WB	1:2000	C67E7
p-AKT	Cell Signaling	4067	Rabbit mAb	WB	1:2000	Ser473
AR	Santa Cruz	sc-7305	Mouse mAb	WB	1:1000	Clone 441; aa 299-315
AR	Santa Cruz	sc-816	Rabbit pAb	IHC	1:1000	N-20; N-terminus
AR	Abcam	ab74272	Rabbit pAb	IHC/IP	1:200	ChIP Grade; N-terminus
AR-V7	Precision Antibody	AG10008	Mouse mAb	WB	1:1000	
β-Actin	Santa Cruz	sc-47778	Mouse mAb	WB	1:4000	C-4
BCL-2	BD Transduction Laboratories	610538	Mouse mAb	WB	1:1000	Clone 7, aa 49-179
CD44	Abcam	Ab51037	Rabbit mAb	WB	1:2000	
c-Myc	Abcam	ab32072	Rabbit mAb	WB	1:2000	
E-Cadherin	Santa Cruz	sc-7870	Rabbit pAb	WB	1:1000	H-108
ERK1/2	Cell Signaling					
p-ERK1/2	Cell Signaling	9101	Rabbit pAb	WB	1:1000	Thr202/Tyr204
FKBP5	Cell Signaling	8245	Rabbit pAb	WB	1:1500	
GAPDH	Santa Cruz	sc-25778	Rabbit pAb	WB	1:5000	FL-335
GR	BD Transduction Laboratories	611227	Mouse mAb	IHC, WB	IHC 1:200<br>WB 1:1000	Clone 41, aa 176-289
Integrin α2	BD Transduction Laboratories	611016	Mouse mAb	WB	1:800	CD49b
Lamin A/C	Santa Cruz	sc-20681	Rabbit mAb	WB	1:4000	H-110
Synaptophysin	Abcam	Ab8049	Mouse mAb	IHC	1:200	
Chromogranin A	NeoMarkers	MS-381-P	Mouus mAb	IHC	1:100	
N-Cadherin	BD Transduction Laboratories	610921	Mouse mAb	WB	1:1000	Clone 32, aa 802-819
NF-kB	Santa Cruz	sc-1190	Goat pAb	WB	1:1000	P50, C-19
NKX3.1	SIGMA	N6036	Rabbit pAb	WB	1:1000	aa 70-83
PSA	Santa Cruz	sc-7638	Goat pAb	WB	1:800	C-19
STAT3	Santa Cruz	sc-7179	Rabbit pAb	WB	1:1000	H-190
p-STAT3	Cell Signaling	9131	Rabbit pAb	WB	1:1000	Try705
TMPRSS2	Millipore	ST1676	Mouse mAb	WB	1:500	

Abbreviations: AR, androgen receptor; GR, glucocorticoid receptor; PSA, prostate specific antigen; IHC, immunohistochemistry; WB, western blotting; IP, immunoprecipitation.

Supplementary Table 2. Molecular changes in xenograft models in Western blots*

Protein	1° CRPC vs. AD				Enza treated vs. 1° CRPC	
	LAPC9	LAPC4	VCaP	LNCaP	LNCaP	LAPC9
AR	↓	↑	↑	↑	↑	-
AR-V7	-	-	↑	-	-	-
PSA	↓	↓	↑	+/-	↓	-
FKBP5	↓	↓	+/-	+/-	↓	+/-
GR	↓	↑	↑	↑(+/-)	↑	+/-
N-Cadherin	↑	↑	↓	-	-	+/-
BCL-2	↑	↑	↓	↑	↑	+/-
p-ERK1/2	↑	↑	↓(+/-)	↑	+/-	↓(+/-)
p-AKT	+/-	↓	+/-	↓	↑	+/-
ALDH7A1	+/-	↓	-	-	-	+/-
c-MYC	↑	+/-	↓	+/-	↑	+/-
p-STAT3	+/-	↓	↓	↑	+/-	+/-
E-Cadherin	↓	+/-	+/-	↓	↑	+/-
Integrin α2	↑	↑	↓	↑	+/-	+/-

*This table summarizes changes in the molecules we analyzed by Western blotting in Figure 2 (for 1° CRPC vs. AD comparisons), Supplementary Figure 11a (for LNCaP 1° CRPC vs. 2° CRPC [Enza-treated] comparisons), and Supplementary Figure 16a (for LAPC9 1° CRPC vs. Enza-treated comparisons). Relative expression levels were determined by densitometric scanning of the bands and normalizing to corresponding GAPDH levels. Upregulation is indicated by red upward arrows whereas downregulation by black downward arrows. +/-: no significant or consistent changes; -, not detected or not done.

Supplementary Table 3. Datasets for GSEA in current study

Figures	Description	Literature
Fig. 7b<br>Fig. 8b	Two gene signatures generated from 7 patients with locally advanced or metastatic PCa before and after ADT treatment	Rajan P et al., *Eur Urol*, 66 (1):32-39(2014)
Fig. 7c<br>Suppl. Fig. 19a	Performed gene expression analysis and developed gene signatures in 15 CRPC-NEPC (from 10 individuals) vs. 34 CRPC-Adeno samples (from 33 individuals)	Beltran H et al., *Nat Med*, 22 (3): 298-305 (2016)
Fig. 7e	Used oligonucleotide microarrays and identified gene signatures upregulated in primary B lymphocytes isolated from Emu-Bcl-2 transgenic mice	Vanasse GJ et al., *Mol Cancer Res*, 2 (11): 620-631 (2004) **(from MSigDB)**
Suppl. Fig. 13a	Performed microarray and generated differential gene expression patterns in 10 androgen-independent (AI) vs 10 androgen-dependent (AD) patient tumors (Used the top 1,000 upregulated genes in AI vs. AD patient samples)	Best CJ et al., *Clin Cancer Res*, 11(19 Pt1): 6823-6824 (2005); Li X et al., *Oncotarget*, 5 (19): 9498-9513 (2014)
	Performed RNA-Seq analysis and compared gene expression patterns in androgen-dependent LNCaP vs. androgen-independent LNCaP-AI-F cells	Wang Y et al., *Cancer Lett*, 342 (1): 130-138 (2014)
Suppl. Fig. 13f	Performed microarray analysis to identify genes upregulated in human neural crest stem (NCS) cells	Lee G et al., *Nat Biotechnol*, 25 (12):1468-1475 (2007)
Suppl. Fig. 13f<br>Suppl. Fig. 18b	Performed microarray analysis and classified gene signatures of glioblastoma multiforme into Proneural, Neural, Classical and Mesenchymal subtypes	Verhaak RG et al., *Cancer Cell*, 17 (1): 98-110 (2010)
Suppl. Fig. 14a	Used RNA-Seq analysis to identify gene expression patterns in purified LNCaP $PSA^{-/lo}$ vs. $PSA^{+/hi}$ cells	Liu X, et al., *manuscript in preparation*
Suppl. Fig. 14b<br>Suppl. Fig. 18d	Established gene expression profiles in normal human prostate basal vs. luminal cells using RNA-Seq analysis	Zhang D, et al., *Nat. Commun*, 7:10798 (2016)
Suppl. Fig. 14e	Microarray analysis to identify genes upregulated in freshly purified stromal stem cells from adipose tissue ($CD31^{+}$) vs. non-stem cells ($CD31^{-}$)	Boquest AC et al., *Mol Biol Cell*, 16 (3):1131-1141 (2005) **(from MSigDB)**
	Compared gene expression signatures of 4 normal mammary subpopulations (mammary stem cells, committed luminal progenitors, mature luminal and stromal cells) isolated from mouse mammary gland and compared with their human counterparts, and identified genes consistently upregulated in mammary stem cells	Lim E et al., *Breast Cancer Res*, 12 (2):R21 (2010) **(from MSigDB)**
	Used previously reported expression data describing the subtypes in 344 primary breast patients who were all lymph-node negative and performed global test program, and identified genes downregulated in luminal B subtype of breast cancer	Smid M et al., *Cancer Res*, 68 (9):3108-3114 (2008) **(from MSigDB)**
	Used whole-genome oligonucleotide microarrays in 31 breast cancer cell lines, and uncovered genes down-regulated in luminal-like cancer cells vs. mesenchymal-like breast cancer cells	Charafe-Jauffret E et al., *Oncogene*, 25 (15):2273-2284 (2006) **(from MSigDB)**
	Introduced EWS-FLI-1 fusion gene to human mesenchymal stem cells (hMSC) and analyzed transcriptional changes via microarray, and identified genes upregulated in hMSC with EWS-FLI-1 fusion protein expression	Riggi N et al., *Cancer Res*, 68 (7):2176-2185 (2008) **(from MSigDB)**
Suppl. Fig. 14f	Curated gene sets specific for Wnt/beta-catenin pathway	Science's Signal Transduction KE **(from MSigDB)**
	Curated gene sets specific for Hedgehog signaling pathway	KEGG gene sets **(from MSigDB)**

	Collected gene sets from previous publications and analyzed the enrichment patterns of gene sets relevant to embryonic stem cell identity from different human tumor types, and found that poorly differentiated tumors show preferential repression of the Polycomb-regulated genes (PRC2 targets)	Ben-Porath I et al., *Nat Genet*, 40 (5): 499-507 (2008) **(from MSigDB)**
	Collected gene sets from previous publications and analyzed the enrichment patterns of gene sets relevant to embryonic stem cell identity from different human tumor types, and found that poorly differentiated tumors show preferential repression of the Polycomb-regulated genes (Suz12 targets)	Ben-Porath I et al., *Nat Genet*, 40 (5): 499-507 (2008) **(from MSigDB)**
	Used microarray to analyze H3K4 methylation patterns in Mll1(+/+) vs. Mll1(-)(/)(-) mouse embryonic fibroblasts, and found genes requiring MLL for H3K4me3 and expression in mouse embryonic fibroblast	Wang P et al., *Mol Cell Biol*, 29 (22): 6074-6085 (2009) **(from MSigDB)**
Suppl. Fig. 14h	Genes involved in phospholipase C mediated cascade provided by the Reactome pathway database	Reactome gene sets **(from MSigDB)**
Fig. 8b	Employed a computational analysis in microarray data obtained from 79 PCa patient samples (40 non-recurrent samples vs. 39 recurrent samples) and established gene profiles. We used the top 1,000 upregulated genes in this dataset for our paper.	Sun Y and Goodison S. *Prostate*, 69 (10): 1119-1127 (2009); Li X et al., *Oncotarget,* 5 (19): 9498-9513 (2014)
Suppl. Fig. 18c	Used microarray analysis to generate gene profiling in gastric mucosal tissue of mice on cholesterol-rich (2%) diet and infected with H. pylori vs. those on cholesterol free (0%) diet and infected with H. pylori. Genes upregulated in this setting were enriched in LAPC9 CRPC vs. AD in our RNA-Seq.	Wunder C et al., *Nat Med*, 12 (9):1030-1038 (2006) **(from MSigDB)**
	Genes involved in immune regulation between lymphoid cells vs. non-lymphoid cells provided by the Reactome gene sets	Reactome gene sets **(from MSigDB)**
	Curated gene sets specific for NK cell-mediated cell killing	KEGG gene sets **(from MSigDB)**
	Genes in endogenous TLR pathway from the Pathway Interaction Database (PID) created between US NCI and Nature Publishing Group	Schaefer CF et al., *Nucleic Acids Res*, 37 (Database issues): D674-679 (2009) **(from MSigDB)**
	Used Affymetrix GeneChips to identify expression levels of 12,435 genes in primary human keratinocytes and fibroblasts overexpressing p50 and p65 (components of NF-κB signaling complex). Genes upregulated in primary fibroblasts were enriched in our RNA-Seq of LAPC9 CRPC vs. AD.	Hinata K et al., *Oncogene*, 22 (13): 1955-1964 (2003) **(from MSigDB)**
	Challenged primary endothelial cells with cytokines eliciting distinct patterns of inflammatory and immune responses in immune cells and used microarray to establish gene profiling in the challenged cells vs. non-challenged cells. Genes upregulated in primary endothelial cells treated with TNF-α were enriched in LAPC9 CRPC vs. AD in our RNA-Seq.	Sana TR et al., *Cytokine*, 29 (6): 256-269 (2005) **(from MSigDB)**
	Curated gene sets specific for antigen processing and presentation	KEGG gene sets **(from MSigDB)**
	Genes involved in antigen processing and cross presentation provided by the Reactome gene sets	Reactome gene sets **(from MSigDB)**
	Used microarray and found the antigen processing and presentation genes to be downregulated in B lymphocytes after HDAC inhibition with trichostatin A (TSA)	Pellicciotta I et al., *Cancer Res*, 68 (19): 8085-8093 (2008) **(from MSigDB)**
	Genes involved in interferon signaling provided by the Reactome gene sets	Reactome gene sets **(from MSigDB)**
	Performed microarray and found gene expression patterns in primary human fibroblasts after human cytomegalovirus	Browne EP et al., *J Virol*, 75(24): 12319-12330 (2001)

	(HCMV) infection. Genes upregulated upon treatment with interferon α for 6 hours were highly enriched in LAPC9 CRPC vs. AD tumors in our RNA-Seq.	(**from MSigDB**)
	Genes involved in interferon α and interferon β signaling provided by the Reactome gene sets	Reactome gene sets (**from MSigDB**)
	Employed a Hu6800 GENECHIP array in HT1080 cells after stimulation for 6 hours with IFN-α, -β, or -γ to explore IFN type-specific changes in gene expression. Genes upregulated after IFN-α treatment were highly enriched in our RNA-Seq of LAPC9 CRPC vs AD.	Der SD et al., *Pro Natl Acad Sci USA*, 95 (26): 15623-15628 (1998) (**from MSigDB**)
	Performed a Hu6800 GENECHIP array in HT 1080 cells after stimulation for 6 hours with IFN-α, -β, or -γ to explore IFN type-specific changes in gene expression. Genes upregulated after IFN-β treatment were highly enriched in our RNA-Seq of LAPC9 CRPC vs. AD.	Der SD et al., *Pro Natl Acad Sci USA*, 95 (26): 15623-15628 (1998) (**from MSigDB**)
	Performed microarrays to examine gene expression patterns in peripheral blood mononuclear cells from multiple sclerosis patients after subcutaneous treatment of IFN-β-1α	Hecker M et al., *Mol Neurobiol*, 48 (3): 737-756 (2013) (**from MSigDB**)
	Genes involved in interferon γ signaling provided by the Reactome gene sets	Reactome gene sets (**from MSigDB**)
	Challenged primary endothelial cells with cytokines eliciting distinct patterns of inflammatory and immune responses in immune cells and used microarray to establish gene expression profile in the challenged cells vs. non-challenged cells. Genes upregulated in primary endothelial cells after IFN-γ treatment were enriched in LAPC9 CRPC vs. AD tumors in our RNA-Seq.	Sana TR et al., *Cytokine*, 29 (6): 256-269 (2005) (**from MSigDB**)
	Used a Hu6800 GENECHIP array in HT1080 cells after stimulation for 6 hours with IFN-α, -β, or -γ to explore IFN type-specific changes in gene expression. Genes upregulated after IFN-γ treatment were highly enriched in LAPC9 CRPC vs. AD tumors in our RNA-Seq.	Der SD et al., *Pro Natl Acad Sci USA*, 95 (26): 15623-15628 (1998) (**from MSigDB**)
