# Supplementary material for: Linking prostate cancer cell AR heterogeneity to distinct castration and enzalutamide responses
Source: Nat Commun. 2018 Sep 6;9:3600. doi: 10.1038/s41467-018-06067-7 (PMC6127155; doi:10.1038/s41467-018-06067-7)
Supplement: Supplementary file 2 — Description of Additional Supplementary Files [file 41467_2018_6067_MOESM2_ESM.pdf]

Description of Additional Supplementary Files:

Supplementary Data 1: Information available on patient samples in the 3 TMAs and 8 whole-mount sections.

Supplementary Data 2: RNA-Seq analysis reveals 2,451 differentially expressed genes (DEGs; FC≥2 and FDR<0.05) preferentially expressed in LNCaP primary CRPC compared to LNCaP AD tumors.

Supplementary Data 3: RNA-Seq analysis reveals 3,254 differentially expressed genes (DEGs; FC≥2 and FDR<0.05) preferentially expressed in LNCaP secondary CRPC compared to LNCaP AD tumors.

Supplementary Data 4: 601 DEGs (FC1.5 and FDR<0.05) preferentially expressed in LNCaP secondary CRPC compared to primary CRPC.

Supplementary Data 5: The 3,929 differentially expressed genes (DEGs) preferentially expressed in LAPC9 CRPC compared to LAPC9 AD tumors in the RNA-Seq analysis (FC≥2 and FDR<0.05).

Supplementary Data 6: The 906 DEGs shared by LAPC9 CRPC (vs. LAPC9 AD tumors) and Beltran CRPC-NE (vs. CRPC-Adeno) in the RNA-Seq analysis (FC≥2 and FDR<0.05).

Supplementary movie 1 legend: Movie 1 illustrates fast proliferative rate of a KO-16 LNCaP cell (labeled with GFP) in Enza (2 μM) containing media. The total recording time was ~140 h. Representative static images (up to 72 h) were shown in Fig. 6a.

Supplementary movie 2 legend: Movies 2 illustrates the slow proliferative rate of a AR-30 LNCaP cell (RFP+) in Enza (2 μM) containing media. The total recording time was ~140 h. Representative static images (up to 72 h) were shown in Fig. 6a.

Supplementary movie 3 legend: Movie 3 shows a single KO-03 cell (labeled with GFP) dividing multiple times in Enza (2 μM) medium.

Supplementary movie 4 legend: Movie 4 shows a single AR-30 cell dying in Enza-containing medium (related to Fig. 6d).
